# RAGE is a Critical Mediator of Pulmonary Oxidative Stress, Alveolar Macrophage Activation and Emphysema in Response to Cigarette Smoke

**DOI:** 10.1038/s41598-018-36163-z

**Published:** 2019-01-18

**Authors:** Karl A. Sanders, Don A. Delker, Tom Huecksteadt, Emily Beck, Tanna Wuren, Yuntian Chen, Yuxia Zhang, Mark W. Hazel, John R. Hoidal

**Affiliations:** 1 Division of Respiratory, Critical Care, and Occupational Pulmonary Medicine, University of Utah, Salt Lake City, Utah USA; 2 Division of Gastroenterology, Hepatology, and Nutrition, University of Utah, Salt Lake City, Utah USA; 3 Department of Internal Medicine, University of Utah, Salt Lake City, Utah USA; 4 George E. Wahlen Department of Veterans Affairs Medical Center, Salt Lake City, Utah USA; 5 Shanghai Ninth People’s Hospital, Shanghai Jiao Tong University School of Medicine, Shanghai, China; 6 Department of Pharmacology, Toxicology and Therapeutics, University of Kansas Medical Center, Kansas City, Kansas USA

## Abstract

The receptor for advanced glycation end products (RAGE), a cell membrane receptor, recognizes ligands produced by cigarette smoke (CS) and has been implicated in the pathogenesis of COPD. We demonstrate that deletion or pharmacologic inhibition of RAGE prevents development of CS-induced emphysema. To identify molecular pathways by which RAGE mediates smoking related lung injury we performed unbiased gene expression profiling of alveolar macrophages (AM) obtained from RAGE null and C57BL/6 WT mice exposed to CS for one week or four months. Pathway analysis of RNA expression identified a number of genes integral to the pathogenesis of COPD impacted by the absence of RAGE. Altered expression of antioxidant response genes and lung protein 4-HNE immunostaining suggest attenuated oxidative stress in the RAGE null mice despite comparable CS exposure and lung leukocyte burden as the WT mice. Reduced endoplasmic reticulum stress in response to CS exposure also was observed in the AM from RAGE null mice. These findings provide novel insight into the sources of oxidative stress, macrophage activation, and the pathogenesis of lung disease due to CS exposure.

## Introduction

By 2030, 8.3 million people each year will die from cigarette smoking-related diseases^1^. Long-term exposure to cigarette smoke (CS) leads to chronic obstructive pulmonary disease (COPD), a devastating disorder for which there is minimal effective treatment. COPD is characterized by irreversible airflow limitation that is usually progressive. The airflow limitation can be due to airway narrowing, inflammation, and excessive airway mucous production (chronic bronchitis); loss of lung elasticity due to alveolar destruction (emphysema); or a combination of the two conditions.

Cigarette smoking exposes the alveolar environment to a variety of particulate and gaseous toxins. Important components of CS are glycotoxins, precursors of advanced glycation end products (AGEs)^2^. AGEs are ligands for the receptor for advanced glycation end products (RAGE)^3^, and the burden of AGEs is increased in COPD subjects compared to smokers without airflow limitation^4,5^. Other RAGE ligands including HMGB1^6^ are increased in the lungs of smokers with COPD compared to non-COPD smokers or never-smokers^7^.

Under basal conditions, RAGE expression is low in all tissues except the lung^8^. It increases in the presence of ligands^9,10^. Consistent with the presence of increased ligands, lung tissue from COPD subjects reportedly has a higher level of RAGE expression^4,7^ than tissue obtained from smokers without COPD. Recent studies demonstrate the association of variants in genomic sequence in RAGE with lung function^11,12^. A consistently identified RAGE polymorphism results in enhanced ligand binding and a cellular inflammatory response^13^, and lower levels of soluble RAGE (sRAGE)^14^. Lower levels of sRAGE correlate with the severity of COPD suggesting it serves to protect against the development of COPD^14,15^, perhaps by acting as a decoy receptor. In population studies RAGE polymorphisms are associated with the diagnosis of COPD^16^.

Evidence suggests that alveolar macrophages (AM) are integral to COPD development^17,18^. Pigmented AM localize to the respiratory bronchioles of smokers early and persist through disease development^19^, suggesting their role in the genesis of COPD. Tasked with defending the alveolar compartment from incursions by the external environment, AM are functionally equipped to be both initiator and perpetuator of lung injury in response to cigarette smoking. Their numbers are inversely correlated with alveolar parenchymal tissue density^20^, and the number of airways occupied by AM increases with COPD progression^21^.

In the present investigation, we confirmed that mice with null mutations for the RAGE gene are protected from airspace enlargement in response to prolonged CS exposure^22,23^. Importantly, treatment of emphysema-susceptible AKR mice^24^ with the RAGE inhibitor FPS-ZM1^25^ also prevented airspace enlargement in response to CS exposure. Next-generation sequencing of RNA samples from AM of wild-type (WT) and RAGE null mice in the presence or absence of a 7 day or 4 month CS exposure revealed that RAGE mediates AM gene expression patterns reflecting oxidative stress, endoplasmic reticulum (ER) stress, and cytokine activity in response to CS. Attenuated expression of AM genes associated with antioxidant responses in RAGE null mice despite comparable CS exposure and lung leukocyte burden as WT mice suggests that RAGE is an important mediator of the oxidative stress that results from smoking. The findings provide novel insight into the central importance of RAGE, the sources of oxidative stress, macrophage activation and the pathogenesis of lung disease due to CS exposure, and offer possible therapeutic options for this devastating disorder.

## Results

### Absence or inhibition of RAGE prevents emphysema following prolonged CS exposure

To initially investigate the importance of RAGE in the smoking model of emphysema, WT and RAGE null mice were exposed to CS or room air for 4 months. Following unbiased sampling, mean linear intercept (L_m_) was determined^26,27^. The L_m_ of WT mice exposed to CS was greater than that of controls (WT + CS = 88.92 +/− 1.94 μm, WT + control = 67.5 +/− 5.8 μm; P < 0.001, Supplementary Fig. 1). The L_m_ of RAGE null mice exposed to CS was not significantly different from controls (RAGE null + CS = 89.7 +/− 1.64 μm, RAGE null + control = 81.05 +/− 4.55 μm). These results confirm earlier reports of enlarged alveolar dimensions in unchallenged RAGE null mice and protection from further airspace enlargement following prolonged CS exposure^22,23^.

**Figure 1:**
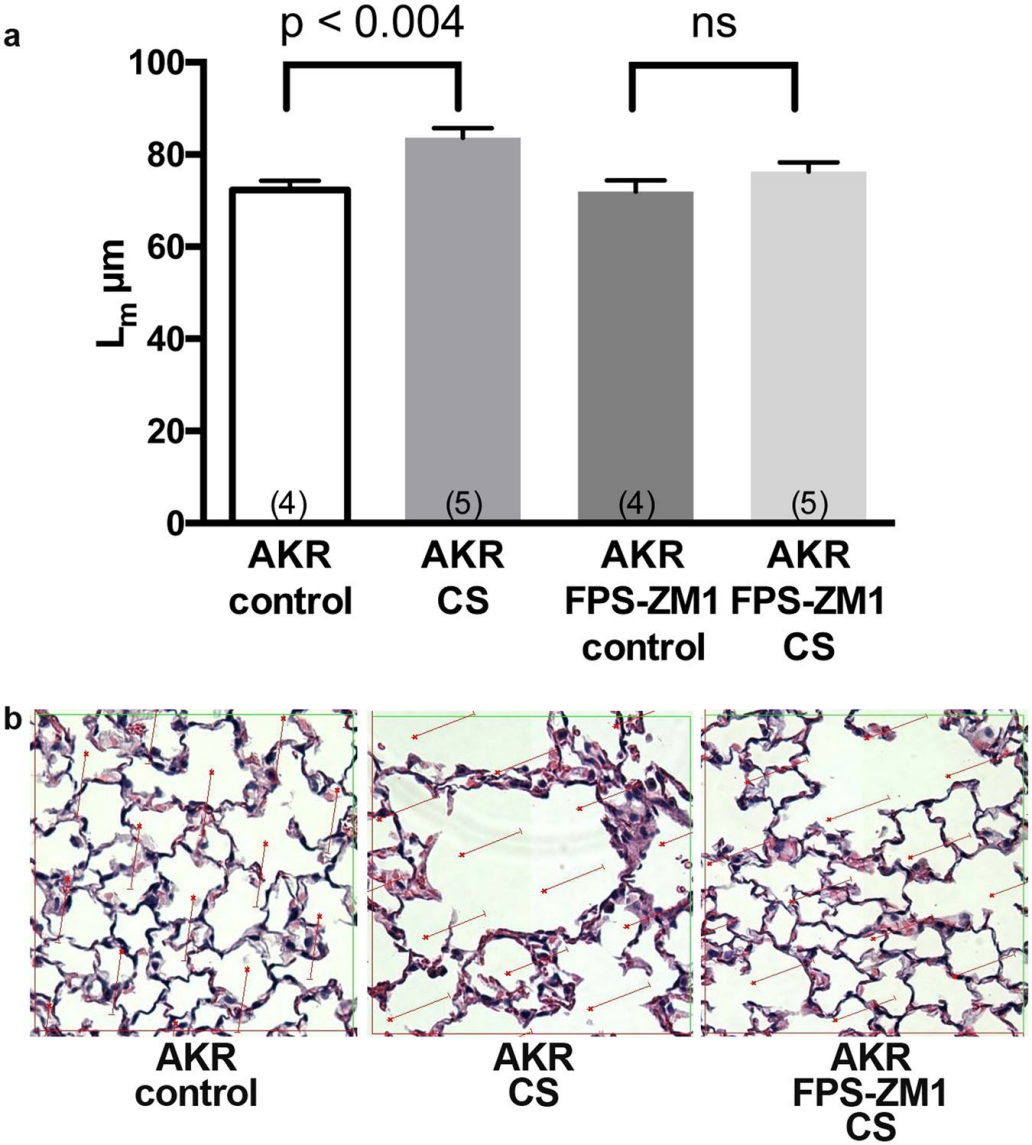
Inhibiting RAGE with FPS-ZM1 suppresses CS-induced airspace enlargement in AKR mice following 2 months of CS exposure. (**a**) L_m_ values demonstrate increased volume-to-surface area in AKR mice exposed to CS, and prevention of the latter with FPS-ZM1. Four or five mice were used in each group. Statistical significance determined by one-way ANOVA. (**b**) H&E stained lung sections suggest increased airspace sizes in CS exposed lungs and prevention with FPS-ZM1.

To more directly evaluate the role of RAGE in CS-induced emphysema in a model free of the potential influence of RAGE on alveolar development and to investigate the possible therapeutic potential of its pharmacologic inhibition we exposed AKR mice to CS in the presence or absence of the RAGE inhibitor FPS-ZM1^25^. We previously demonstrated that AKR are more susceptible than C57BL/6 mice to emphysema development upon exposure to CS^24^. In addition, AKR mice develop emphysema after only 2 months of CS exposure^28^ making them more suitable for initial inhibitor studies. In AKR mice, the L_m_ increased from 72.33 +/− 1.98 μm to 83.66 +/− 2.03 μm; (p < 0.003) following 2 months of CS exposure (Fig. 1a). In contrast, there was no significant increase in L_m_ following CS exposure in mice treated with FPS-ZM1 (L_m_ = 76.32 +/− 1.96 μm) as compared with treated controls (L_m_ = 71.95 ± 2.43 μm). The morphometric data was reflected on histological examination. (Fig. 1b) These findings demonstrate that RAGE mediates emphysema due to CS exposure in more than one strain of mice. As is the case with the RAGE null mutation, RAGE inhibition also prevents emphysema due to CS exposure.

### Absence or inhibition of RAGE does not reduce bronchoalveolar lavage (BAL) cell counts following acute or chronic CS exposure

Neither WT nor RAGE null mice had a statistically significant increase in BAL cell counts after 7 days of CS exposure, although clear trends were present in both groups. (Supplementary Fig. 2A). After 4 months of CS exposure, only exposed RAGE null mice had significantly increased numbers of cells in BAL (Supplementary Fig. 2B). Cells obtained by BAL from CS-exposed WT and RAGE null mice almost exclusively (>98%) displayed morphologies of macrophages at both time points.

After 2 months of CS exposure the AKR mice had significantly increased BAL cell counts as compared with controls. Administration of FPS-ZM1 to AKR mice for 2 months also significantly increased BAL cells counts as compared with untreated controls. However, there was no additional increase in BAL cell counts following two months of CS exposure in the FPS-ZM1 treated AKR mice (Supplementary Fig. 2C).

### Sequencing of AM RNA following 7 days of CS expsoure reveals important differences between WT and RAGE null mice

**Figure 2:**
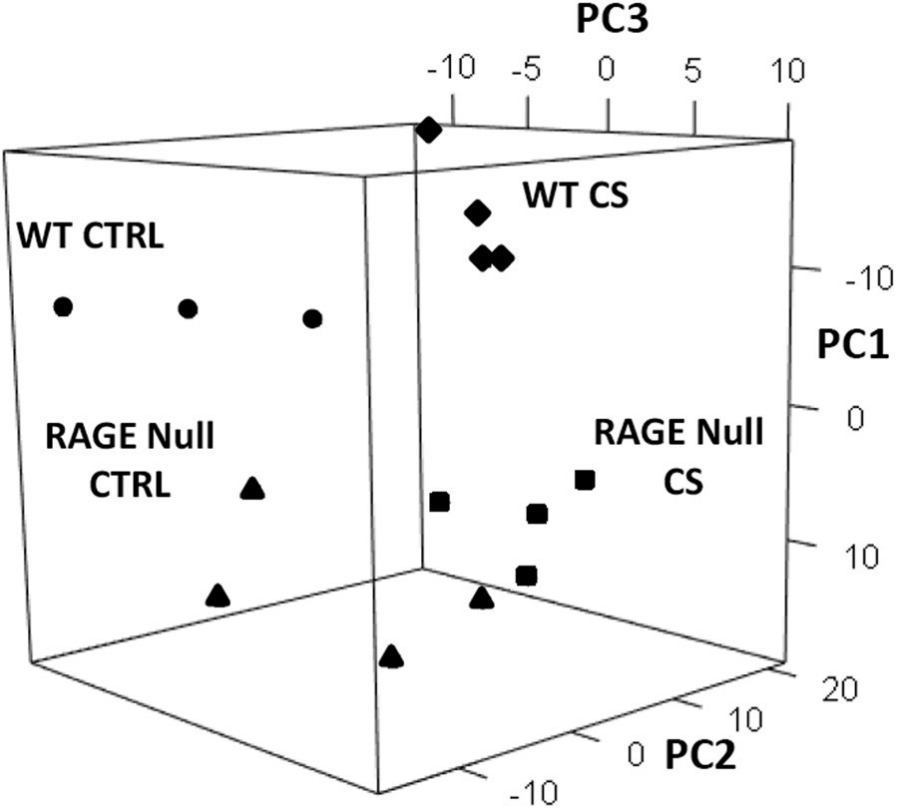
Principal component analysis (PCA) demonstrates that patterns of gene expression are significantly different in AM from WT and RAGE null mice exposed to CS for 7 days. 355 genes differentially expressed (Fold change ≥ 1.5, FDR < 0.05) by genotype and/or CS were used for PCA. The expression of each gene from each mouse sample was compared to its average expression across all samples to generate log_2_ ratios. Principal component (PC)1 accounted for 21% of the variation in the data and separated samples by genotype. PC2 and PC3 accounted for 15% and 13% of the variation in the data, respectively, and separated samples by CS exposure. Individual mouse samples are shown for WT-CTRL mice (n = 3, circles), WT-CS mice (n = 4, diamonds), RAGE null-CTRL mice (n = 4, triangles) and RAGE null-CS mice (n = 4, squares).

To pursue an unbiased investigation of mechanism(s) by which RAGE mediates emphysema development in CS-exposed mice, we investigated the effect of CS exposure on RNA transcript expression of AM from WT and RAGE null mice. AM recovered from WT and RAGE null mice after 7 days of exposure to CS or control conditions differentially expressed 355 genes using a fold change cutoff of 1.5 and FDR <0.05 (Supplementary Fig. 3 and Supplementary Table 1). Further assessment used principal component analysis (PCA) to identify the basis for patterns of genotype and CS-dependent gene expression changes (Fig. 2). PCA identifies gene signatures associated with each experimental variable. Principal component (PC)1 accounted for 21% of the variation in the data and identified genes differentially expressed by genotype. PC2 and PC3 accounted for 15% and 13% of the variation in the data, respectively, and identified genes differentially expressed by CS exposure. Each of the 12 remaining principal components accounted for less than 9% of the variation observed in the RNA sequencing data.

To identify biological mechanisms responsible for differences in gene expression in AM due to either genotype and/or CS exposure we used Ingenuity Pathway Analysis (IPA) software. Differential expression was initially assessed in BAL recovered AM from control RAGE null mice compared to WT mice (Supplementary Tables 2 and 3). Growth factor signaling including genes regulated by transforming growth factor beta 1 and connective tissue growth factor were increased in RAGE null mice compared to WT mice. Gene expression patterns also predicted activation by beta-estradiol, endothelin 1 and protein phosphatase 3 regulatory subunit B alpha in RAGE null mice compared to WT mice. In contrast, the sequencing data predicted that the absence of RAGE would reduce expression of genes regulated by nitric oxide signaling, transcription factor zinc finger E-box binding homeobox 1 and cytochrome P450 family 1 subfamily A member 1. These findings suggest that in the unchallenged state the null mutation of RAGE impacts AM expression of target genes regulated by upstream mediators with relevance to important biological mechanisms including tissue growth, signaling, vascular function, and chemical metabolism.

**Figure 3:**
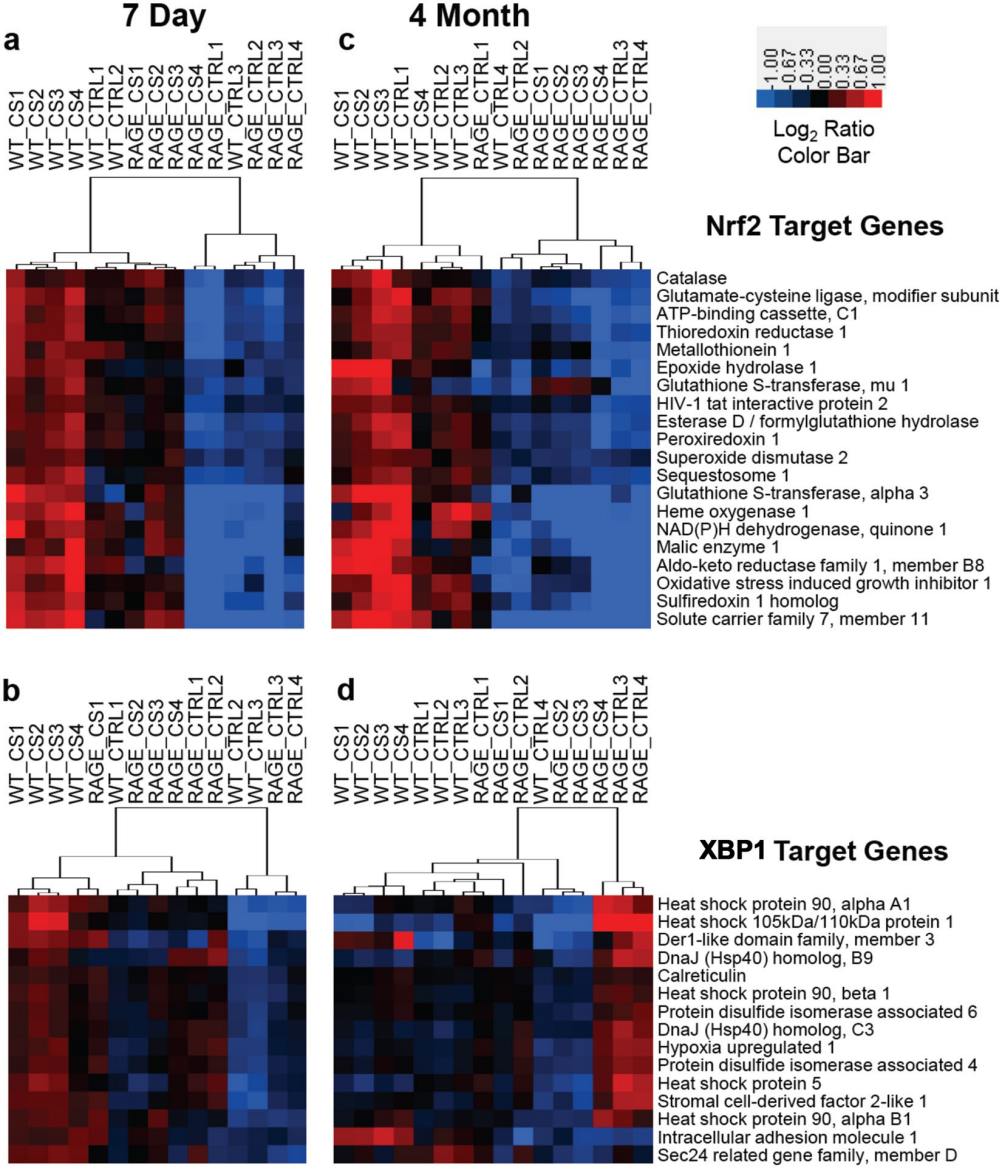
Expression of Nrf2 and XBP1 target genes by AM following 7 days or 4 months of CS exposure is impacted by the absence of RAGE. The relative expression of twenty Nrf2 and fifteen XBP1 target genes were evaluated by hierarchical clustering. (**a**) Nrf2 target genes at 7 days. (**b**) XBP1 target genes at 7 days. (**c**) Nrf2 target genes at 4 months. (**d**) XBP1 target genes at 4 months. Log_2_ ratios comparing each AM RNA sample to the average of all samples were used for Euclidian distance clustering. Red denotes increased expression, blue decreased expression and black no change.

**Figure 4:**
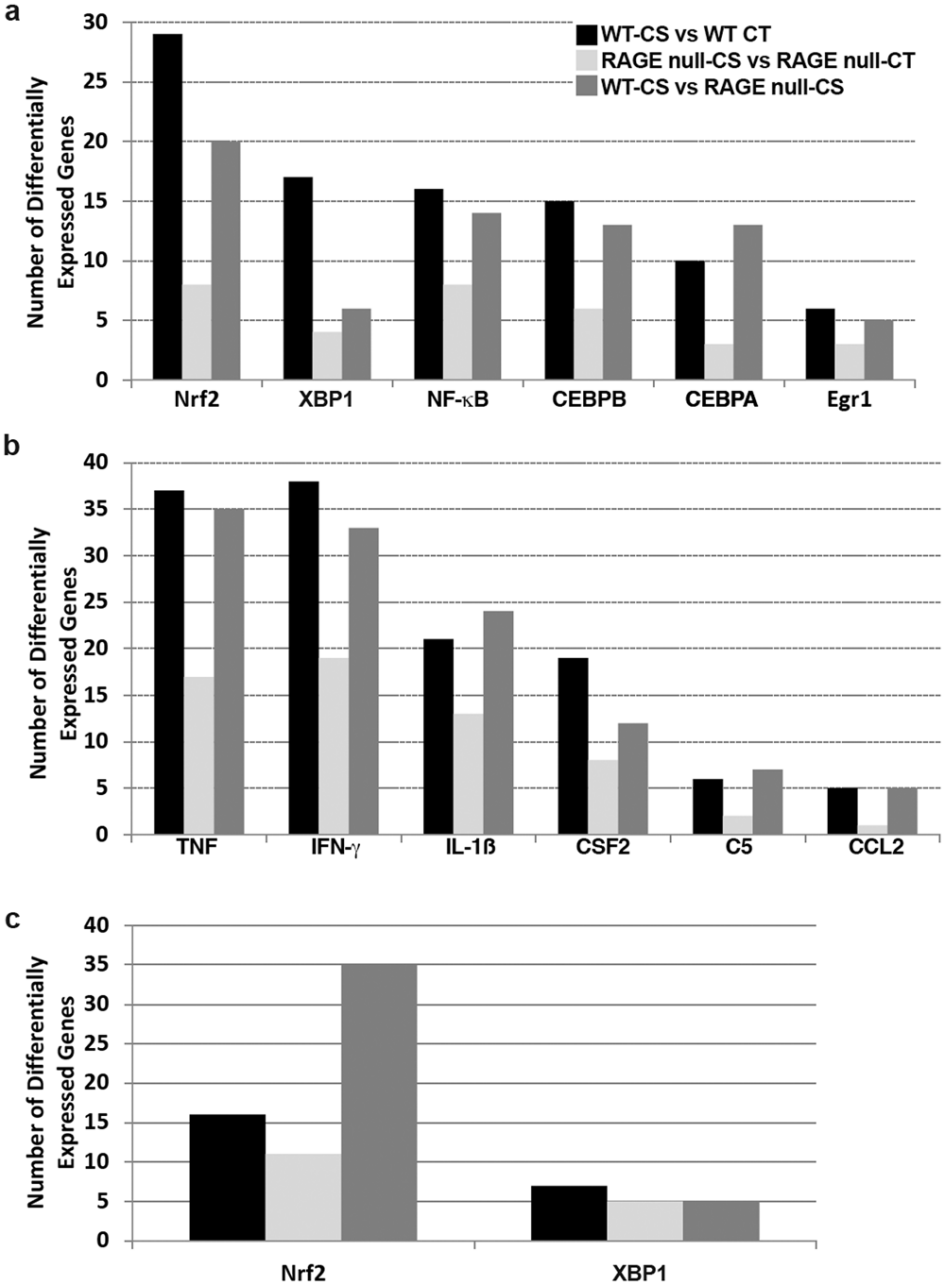
The absence of RAGE reduces expression of genes activated by key upstream regulators in AM following exposure to CS for 7 days or 4 months. (**a**) Number of differentially expressed genes activated by each transcription factor after 7 days CS exposure. (**b**) Number of differentially expressed genes activated by each cytokine after 7 days CS exposure. (**c**) Comparison of the total number of differentially expressed target genes for Nrf2 and XBP1 after a 4-month CS exposure. Differentially expressed genes had a fold change ≥ 1.5 and false discovery rate < 0.05. The number of differentially expressed genes from four comparisons are shown: WT-CS vs WT-CT (CS-exposed WT compared to control WT mice, *black bars*), RAGE-CS vs RAGE-CT (CS-exposed RAGE null compared to control RAGE null mice, *grey bars*) and WT-CS vs RAGE-CS (CS-exposed WT compared to CS-exposed RAGE null mice, *dark grey bars*). The name, fold change and prediction state based on direction of fold change for each gene is shown in Supplementary Tables 4 (transcription factors) and 5 (cytokines).

The top upstream regulators predicted to be responsible for the gene expression differences observed in WT mice exposed to CS for 7 days are shown in Table 1 and Supplementary Table 4. Nuclear factor, erythroid-derived 2, like 2 (Nrf2) and X-box binding protein 1 (XBP1) were among the transcription factors with the highest z-score and lowest p-value, suggesting increased oxidative and endoplasmic reticulum (ER) stress in CS-exposed compared to control WT mice [Fig. 3a (Nrf2) and Fig. 3b (XBP1)]. Among the Nrf2 target genes most highly overexpressed by CS were heme oxygenase 1, carbonyl reductase 2 and thioredoxin reductase 1 (*Txnrd1*) (Supplementary Table 4). Transcripts of heat shock protein genes *Hsph1*, *Hspa5* and *Hsp90aa1* were among the XBP1 target genes most highly expressed by CS. Genes regulated by tumor necrosis factor (TNF) and interleukin-1 beta (IL-1β) were also significantly overexpressed in response to CS (Table 1 and Supplementary Table 5). The relative number of target genes differentially expressed for each upstream regulator that suggests activation is shown in Fig. 4a (transcription factors) and Fig. 4b (cytokines).

**Table 1:** Upstream regulators predicted to be activated in AM from WT and RAGE null mice exposed to cigarette smoke for 7 days.

Upstream Regulator	Type	WT-CT/RAGE-CT		WT-CS/WT-CT		RAGE-CS/RAGE-CT		WT-CS/RAGE-CS	
		z-score	p-val	z-score	p-val	z-score	p-val	z-score	p-val
Nuclear factor, erythroid derived 2, like 2	TF	0.72	1.71E-05	**3**.**46**	**3**.**40E-17**	1.59	2.30E-05	**2**.**24**	**2**.**10E-12**
Nuclear factor kappa in B cells	TF	−0.02	1.42E-06	1.26	1.20E-07	0.04	9.90E-07	**2**.**66**	**1**.**60E-06**
CCAAT/enhancer binding protein, beta	TF	0.01	8.58E-05	1.69	3.30E-09	1.74	1.90E-07	**2**.**18**	**8**.**70E-08**
CCAAT/enhancer binding protein, alpha	TF	−0.12	3.92E-08	**2**.**18**	**2**.**60E-04**	1.36	1.30E-04	0.64	7.60E-08
X-box binding protein 1	TF	ns	ns	**3**.**36**	**1**.**00E-10**	ns	3.20E-03	1.41	2.10E-02
Early growth response 1	TF	−1.51	1.54E-07	**2**.**39**	**3**.**00E-04**	1.06	1.40E-03	**2**.**39**	**2**.**00E-04**
Tumor necrosis factor	Cytokine	0.02	1.32E-13	**2**.**96**	**9**.**60E-14**	0.52	2.80E-09	**2**.**75**	**4**.**80E-14**
Interferon gamma	Cytokine	0.44	8.39E-18	0.54	2.10E-18	−0.87	6.60E-17	**2**.**83**	**3**.**40E-15**
Interleukin 1, beta	Cytokine	−0.59	6.49E-12	**2**.**08**	**1**.**90E-08**	−0.25	1.30E-13	**2**.**45**	**2**.**80E-12**
Colony stimulating factor 2	Cytokine	−0.57	3.06E-06	1.42	6.20E-11	0.1	5.80E-05	1.57	3.70E-06
Complement component 5	Cytokine	−0.42	2.39E-03	**2**.**38**	**4**.**30E-05**	ns	4.60E-02	**2**.**58**	**2**.**10E-06**
Chemokine (C-C motif) ligand 2	Cytokine	ns	9.41E-03	**2**.**21**	**1**.**40E-05**	ns	1.20E-02	**2**.**23**	**9**.**50E-06**

Seven-day CS and genotype comparisons. Results from four comparisons are shown including genotype comparisons between control and CS-exposed mice. Table denotes name and type [transcription factor (TF) or cytokine] of upstream regulator together with Fisher exact overlap p-value and activation Z-score as output from Ingenuity Pathway Analysis (IPA). The overlap p-value calls likely upstream regulators based on significant overlap between differentially expressed genes and known gene targets. The activation Z-score infers activation or inhibition of upstream regulators based on the direction of fold change of differentially expressed target genes. Comparison values with z-score statistics >2 or <−2 are shown in bold.

When comparing AM from CS-exposed and control RAGE null mice, none of the genes regulated by those transcription factors or cytokines found overexpressed in AM from CS-exposed WT mice reached a significant z-score > 2 (Table 1). While there were several target genes associated with each upstream regulator found differentially expressed in AM from CS-exposed compared with control RAGE null mice, this number was dramatically reduced as compared with WT mice (Fig. 4a,b, Supplementary Tables 4 and 5). These results show that the absence of RAGE significantly reduces AM gene expression changes associated with oxidative stress, ER stress, and cytokine activity in response to CS exposure.

To identify smoking-related biological pathways and disease processes directly influenced by the absence of RAGE, we compared AM gene expression between CS-exposed WT and CS-exposed RAGE null mice (Table 1, Supplementary Tables 4 and 5). Genes predicted to be regulated by the same upstream regulators (Nrf2, TNF, IL-1β, Egr1, C5, CCL2) as reported for the WT CS-exposed versus WT control comparison were overexpressed in the WT CS-exposed versus RAGE null CS-exposed comparison. In addition, this comparison predicted activation of genes regulated by additional upstream regulators including nuclear factor kappa-light-chain-enhancer of activated B-cells (NF-κB), CCAAT/enhancer binding protein beta, and interferon gamma (IFN-γ). In some cases, the WT-CS vs RAGE-CS comparison resulted in increased statistical significance (Table 1, Supplementary Tables 4 and 5) and/or more differentially expressed target genes (Fig. 4a,b) as compared with the WT CS exposed versus WT control comparison providing additional evidence of the role of RAGE in overexpression of those pathways in response to CS exposure.

### Sequencing of AM RNA following 4 months of CS expsoure reveals important differences between WT and RAGE null mice and between acute and chronic CS exposure

The AM from WT and RAGE null mice exposed to either air or CS for 4 months also were analyzed by RNA sequencing. The AM from control RAGE null mice continue to express more genes regulated by upstream mediators with relevance to tissue growth and vascular function (Supplementary Table 3). XBP1 target gene expression was higher in AM from control RAGE null mice compared to WT mice after the 4-month period (Fig. 3d). Gene expression mediated by Nrf2 was greater in the AM from control WT mice than age-matched RAGE null mice. (Fig. 3c). After 4 months of CS exposure the AM from WT mice continued to express more Nrf2 target genes than RAGE null AM. (Table 2, Figs 3c and 4c). No increase in XBP1 target gene expression was observed after 4 months of CS exposure in AM of either genotype (Table 2, Figs 3d and 4c). When compared with AM from respective age-matched controls decreased expression of pathways mediated by NF-κB, TNF, and IL-1β was observed after 4 months CS exposure in both mouse genotypes, with greater reduction observed in the RAGE null AM (Table 2).

**Table 2:** Upstream regulators predicted to be activated in AM from WT and RAGE null mice exposed to cigarette smoke for 4 months.

Upstream Regulator	Type	WT-CT/RAGE-CT		WT-CS/WT-CT		RAGE-CS/RAGE-CT		WT-CS/RAGE-CS	
		z-score	p-val	z-score	p-val	z-score	p-val	z-score	p-val
Nuclear factor, erythroid derived 2, like 2	TF	1.36	8.62E-15	1.54	3.48E-05	−1.36	1.16E-06	**2**.**25**	**1**.**72E-22**
Nuclear factor kappa in B cells	TF	0.26	6.57E-08	**−2**.**16**	**8**.**87E-15**	**−2**.**80**	**8**.**75E-17**	**4**.**09**	**2**.**70E-16**
X-box binding protein 1	TF	−0.26	8.09E-03	0.17	6.86E-03	−1.64	3.35E-06	ns	ns
Tumor necrosis factor	Cytokine	−1.20	1.71E-20	**−2**.**10**	**4**.**84E-25**	**−5**.**26**	**1**.**82E-33**	**3**.**90**	**5**.**88E-22**
Interleukin 1, beta	Cytokine	**−2**.**54**	**6**.**16E-19**	−1.87	1.41E-25	**−4**.**42**	**4**.**21E-37**	**2**.**07**	**5**.**55E-22**

Results from four comparisons are shown. Table denotes name and type [transcription factor (TF) or cytokine] of upstream regulator together with Fisher exact overlap p-value and activation Z-score as explained in Table 1. Comparison values with z-score statistics >2 or <−2 are shown in bold.

### Quantification of gene products and nuclear proteins validates the RNA sequencing differences between WT and RAGE null mice AM following 7 days of CS exposure

**Figure 5:**
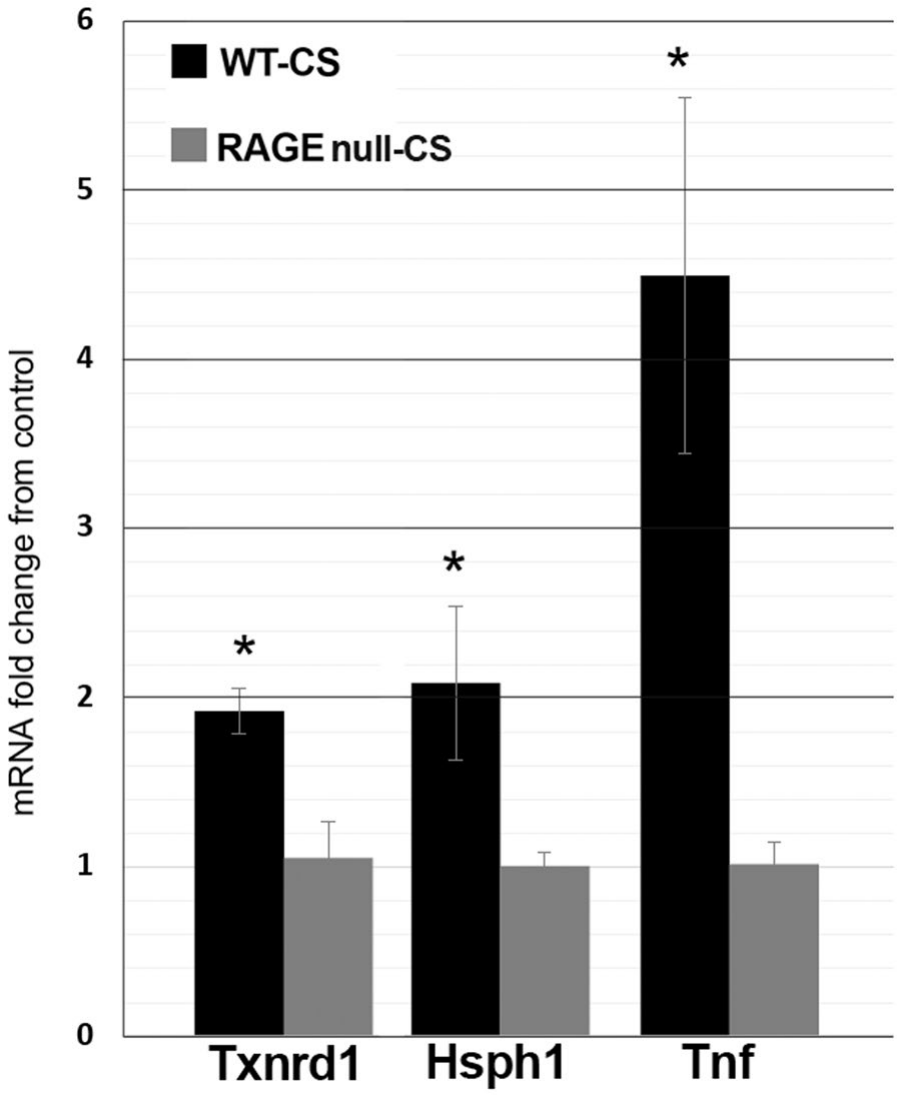
Quantitative PCR validates the RNA sequencing differences between WT and RAGE null mice AM following 7 days of CS exposure. Thioredoxin reductase 1 **(**Txnrd1*)* was used to verify RNA sequencing findings related to oxidative stress, heat shock 105 kDa/110 kDa protein 1 **(**Hsph1*)* for ER stress, and tumor necrosis factor **(**Tnf) for cytokine production. Values represent the mean and standard error of 3 mice per group. * Denotes statistical significance from CS-exposed RAGE null mice (p < 0.05) by Student’s t-test. Black and grey bars represent CS-exposed WT and CS-exposed RAGE null mice, respectively. The results are expressed as fold change of gene expression in CS-exposed over control AM values for each mouse genotype.

Quantitative PCR was used to confirm the RNA sequencing findings of 3 representative mRNAs from AM of CS-exposed WT versus RAGE null mice. Each denoted a target gene of a specific biological pathway influenced by CS exposure: Txnrd1 for oxidative stress, Hsph1 for ER stress and Tnf for cytokine production (Fig. 5). Each of these mRNAs was increased in CS-exposed AM from WT mice compared to RAGE null mice (*Fold* > *1*.*9*, *p* < *0*.*05*). Nuclear protein from AM underwent ELISA to assess the presence of upstream regulators identified as likely mediating the RNA sequencing findings. Nuclei from AM of RAGE null mice had 29% less NF-κB (p65) protein (Supplementary Fig. 4a) and 49% less Nrf2 protein (Supplementary Fig. 4b) than did WT mice following 7 days of CS exposure.

### Seven days of CS exposure does not increase markers of oxidative stress in lungs from RAGE null mice

**Figure 6:**
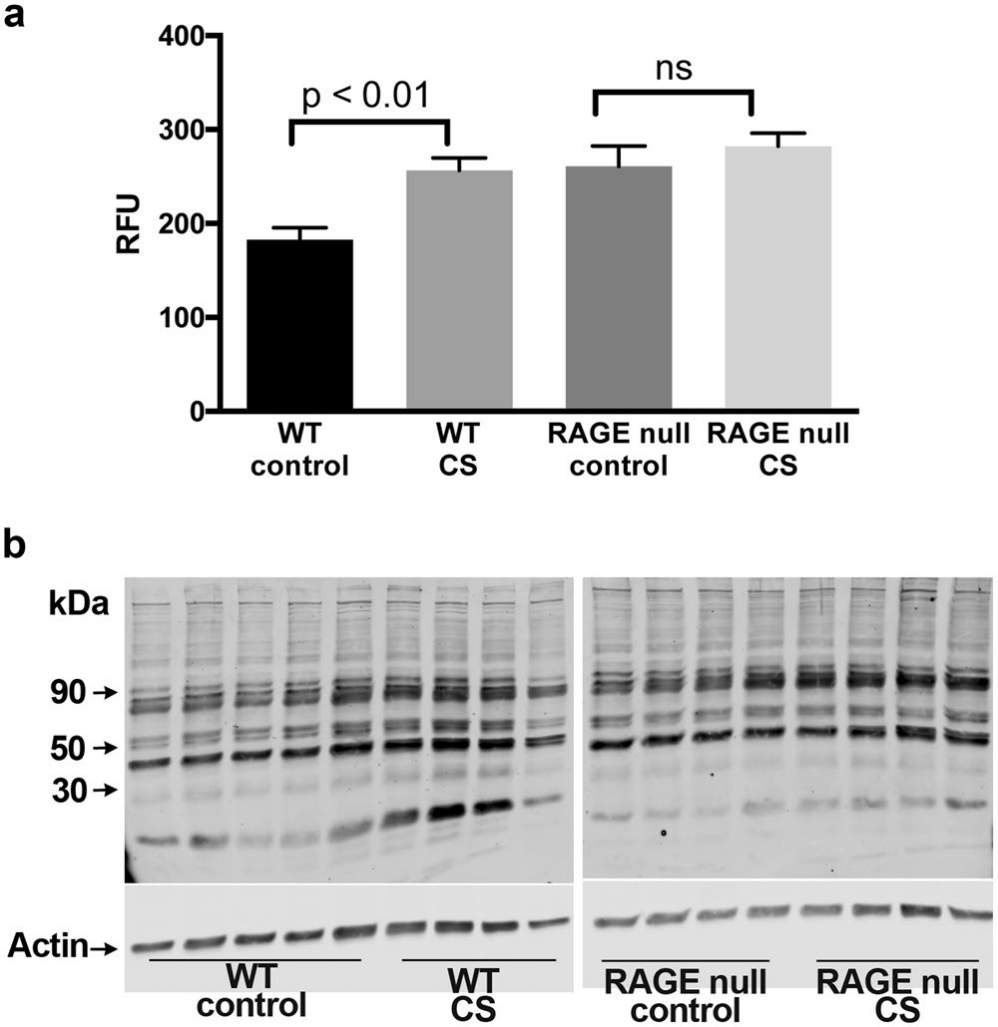
Seven days of CS exposure does not increase markers of oxidative stress in lungs from RAGE null mice. (**a**) Quanitification of western blot data. Statistical significance determined by Student’s t-test. Lungs from control WT mice (5) or all other groups (4) of male mice were analyzed. (**b**) Full-length gels of 4-HNE-modified proteins analyzed by western blotting using lung homogenates from control and CS-exposed mice. 4-HNE modified proteins were increased in WT lungs by CS exposure. Although increased in control RAGE null lungs as compared with WT lungs, 7 days of CS did not result in an additional increment in 4-HNE staining in lungs of RAGE null mice.

The greater expression of genes regulated by Nrf2 in AM obtained from CS-exposed WT mice compared to RAGE null mice suggested differences in oxidative stress. To assess oxidative stress, protein samples from mouse lung tissue underwent western blotting with anti-4-HNE antibodies. The lungs from WT mice demonstrated increased 4-HNE-modified proteins following 7 days of CS exposure (Fig. 6). Lungs from control RAGE null mice had increased 4-HNE-modified proteins as compared with WT mice. However, in RAGE null mice there was no further increase in 4-HNE-modified proteins following 7 days of CS exposure. As CS exposure was identical in both groups of mice and BAL cell counts did not show a reduction in AM in CS exposed RAGE null mice (Supplementary Fig. 2a) we questioned whether the findings reflected fewer neutrophils in the lung tissue of the RAGE null mice. Dissociated lung tissue analyzed by FACS demonstrated comparable percentages of neutrophils in WT and RAGE null lung tissue in the presence or absence of 7 days CS exposure (Supplementary Fig. 5).

### Seven days of CS exposure increases AM ER stress proteins by a RAGE-dependent mechanism

**Figure 7:**
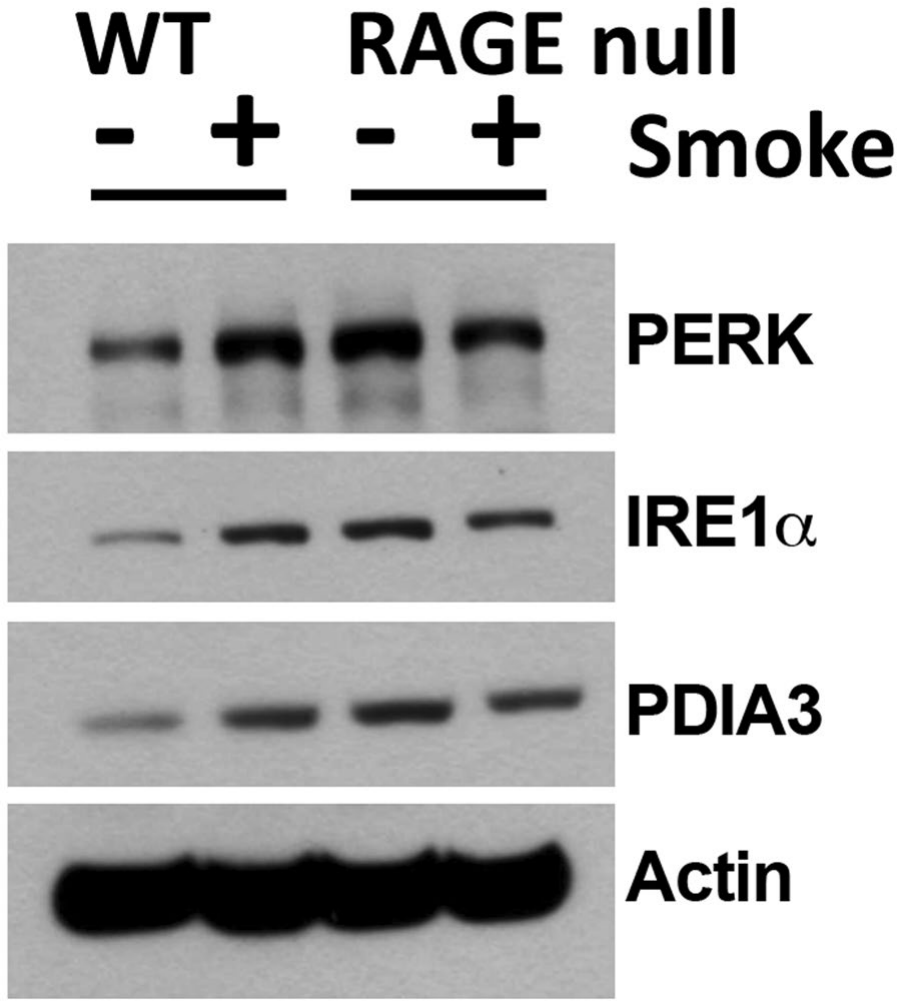
Seven days of CS exposure does not increase ER Stress proteins in AM from RAGE null mice. Western blotting was performed on AM proteins using antibodies targeting protein involved in the ER stress response including protein kinase R-like endoplasmic reticulum kinase (PERK), inositol requiring enzyme 1α (IRE1α) and protein disulfide isomerase family A member 3 (PDIA3). Cell lysates were pooled from 3–4 male mice per group. Immunoblots show the basal level of each ER stress protein was higher in control RAGE null compared to WT mice. However, CS exposure increased the expression of all three proteins in only WT mice. Full length gels are shown in Supplementary Fig. 7.

To evaluate the impact of differences in ER stress gene expression in AM from CS exposed WT and RAGE null mice, we assessed expression of selected ER stress proteins^29^. ER stress proteins including protein kinase R-like endoplasmic reticulum kinase (PERK or EIF2AK3), inositol requiring enzyme 1 (IRE1α, or ERN1), and protein disulfide isomerase family A member 3 (PDIA3) were increased in WT AM lysates following 7 days of CS exposure (Fig. 7). ER stress proteins in AM from RAGE null mice were higher than WT mice at baseline. However, there was no increment in ER stress protein expression due to CS in AM from RAGE null mice.

### Differences in AM gene expression between WT and RAGE null mice following 7 days CS exposure is not due to RAGE expressed in those cells

**Figure 8:**
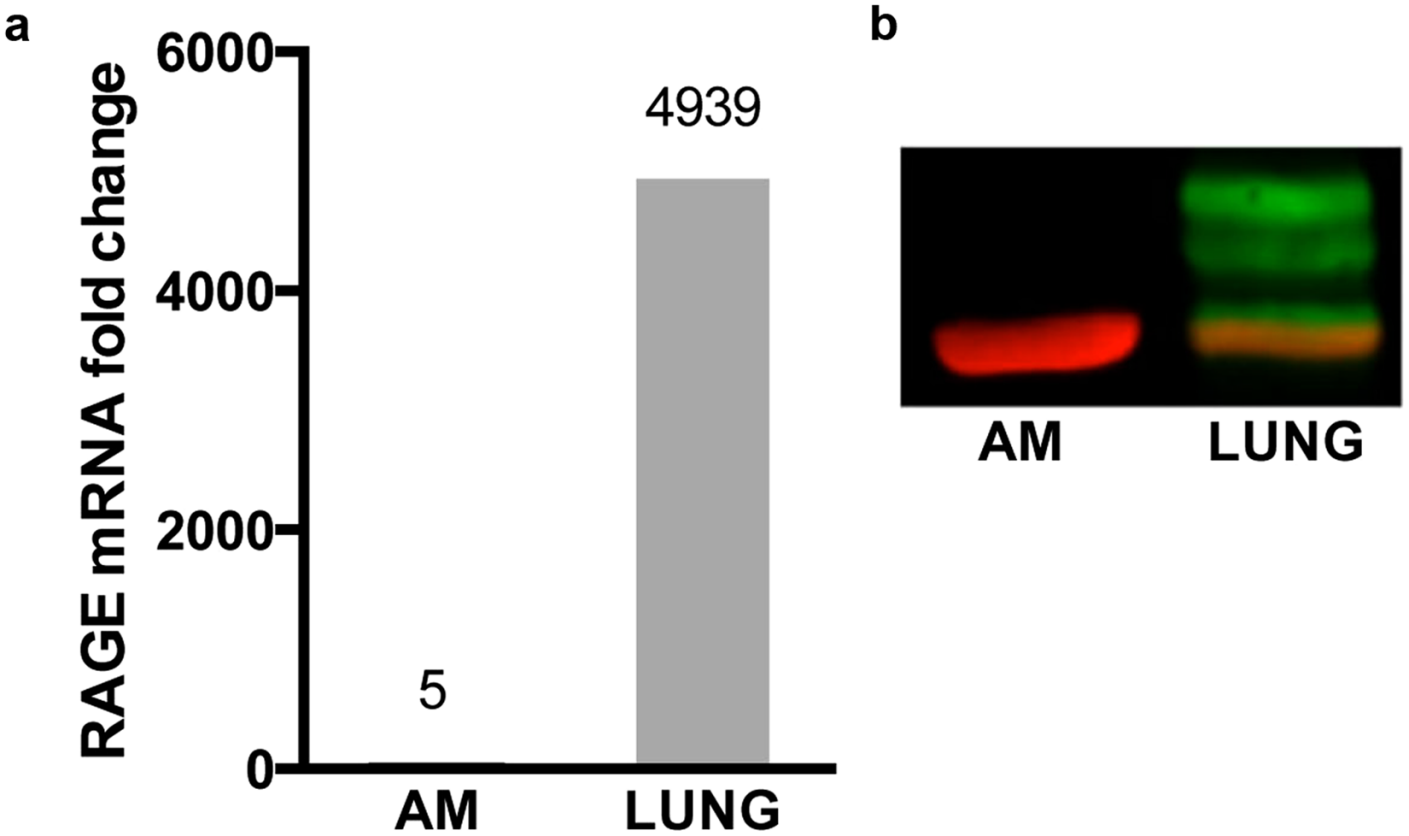
Differences in AM gene expression between WT and RAGE null mice following 7 days CS exposure is not due to RAGE expressed in those cells. (**a**) RAGE mRNA expression in AM and lung tissue. Equal quantities of mRNA from each tissue underwent reverse transcription and subsequent quantitative PCR. Data is expressed as fold increase over basal expression of RAGE mRNA in control WT AM. The findings are representative of numerous prior experiments. (**b**) RAGE protein is present in lung tissue but undetected in AM. Equal amounts of protein from each tissue underwent western blotting. RAGE bands are green and actin is red. Samples were run on protein from AM from 2 mice and lung tissue from 3 mice (males and females). Full length gels are shown in Supplementary Fig. 7.

To determine the source of RAGE responsible for the differences in gene expression in the experimental groups we performed immunohistochemistry on lung tissue from WT and RAGE null mice. RAGE immunostraining was absent in RAGE null tissue, demonstrating the specificity of the anti-RAGE antibody (Supplementary Fig. 6a). RAGE immunostaining was detected primarily in alveolar epithelium and with faint staining of AM in control lungs, without observable increase with 4 months of CS exposure (Supplementary Fig. 6b). In contrast, RNA-sequencing data demonstrated minimal RAGE expression in control or CS-exposed WT AM (Supplementary Fig. 6c). To further investigate RAGE expression in AM, we performed RT-qPCR on total mRNA obtained from WT AM and post-BAL lung tissue after 7 days CS exposure. Very minimal RAGE expression was detected in AM (Fig. 8a). In contrast, pronounced RAGE expression was detected in post-BAL lung tissue. Western blotting with anti-RAGE antibodies confirmed undetectable RAGE protein in AM and prominent RAGE protein in post-BAL lung tissue (Fig. 8b).

## Discussion

Animal and human evidence implicates RAGE in the pathogenesis of lung disease due to CS exposure^4,7,22,23,30,31^. We^32^ and others^22,23^ have observed that mice deficient in RAGE have reduced lung inflammatory mediators and are protected from injury due to CS exposure. We have confirmed that RAGE null mice are protected from CS-induced airspace enlargement in our smoking model. The RAGE blocker FPS-ZM1 also prevented emphysema due to CS in AKR mice. These results verify the role of RAGE in the pathogenesis of emphysema and for the first time indicate that pharmacologic RAGE inhibition may be a protective strategy in CS-induced lung injury.

Numerous lines of evidence suggest that AM play a pivotal role in the development of COPD/emphysema^17–21^. We questioned whether differences in AM gene expression might provide insight into how RAGE null animals or RAGE inhibition protects from the injurious effects of CS exposure. By interrogating cells prior to and early in the establishment of morphometric evidence of emphysema, we hoped to identify gene expression pathways integral to the initiation of emphysema. By employing a transcriptomic approach we obtained an unbiased assessment of AM gene expression. RNA sequencing and IPA analysis suggested that AM recovered from WT mice exposed to 7 days of CS are simultaneously contending with oxidative and ER stress, and expressing gene products with immediate relevance to the innate immune response. These patterns were not present in AM from RAGE null mice exposed to CS suggesting that RAGE is integral to AM activation in response to CS.

The most striking difference between AM from smoke-exposed WT and RAGE null mice was seen in gene expression mediated by Nrf2. Nrf2 regulates genes involved in antioxidant responses, xenobiotic detoxification, and proteome maintenance^33^. Oxidative stress is thought integral to the pathogenesis of lung destruction due to CS^34,35^. Evidence of this has been provided by the increased predisposition to emphysema of mice possessing null mutations for Nrf2 in response to CS exposure^36,37^. Proposed sources of oxidative stress due to smoking include macrophages, neutrophils, and CS^34,38^. Expression of Nrf2 target genes was strikingly increased in AM from CS exposed WT mice as compared with exposed RAGE null mice. The absence of both Nrf2-mediated gene expression in AM and increases in 4-HNE proteins in lung tissue suggests that RAGE null mice have less CS-induced pulmonary oxidative stress than WT mice. Exposure to oxidants contained within CS was identical in both strains of mice. As compared with WT mice, RAGE null mice had similar numbers of AM in BAL and percentages of CD45 positive cells with a neutrophil phenotype in lung tissue after 7 days of CS exposure and increased numbers of AM in BAL after 4 months CS exposure. This suggests that the source(s) of oxidative stress due to CS arise primarily from endogenous, non-leukocyte, RAGE-mediated mechanisms.

We also observed greater gene expression changes mediated by the transcription factor XBP1 in CS-exposed WT AM as compared with RAGE null AM. XBP1 mediates transcription of gene products that participate in the unfolded protein response (UPR) and consequent ER stress^39^. ER stress due to oxidative modifications of proteins is thought to contribute to the pathogenesis of CS-induced lung disease^40^. Proteins involved in the UPR are increased in the lungs of smokers as compared with non-smokers^41^ and findings of ER stress due to disturbed proteostasis correlate with degrees of lung dysfunction in patients with COPD^42^.

As expected, *in vivo* exposure of AM from WT mice to CS also increased expression of proteins with relevance to the UPR including PERK, IRE1α, and PDIA3. Although increased at baseline, UPR proteins were not further increased by CS exposure in AM from RAGE null mice. This may reflect attenuated oxidative stress and consequent protein modifications and/or other aspects of ER function in these mice compared to CS-exposed WT mice. The increased expression of ER stress proteins at baseline in RAGE null AM may reflect altered ER protein handling due to an as yet undefined abnormality in these cells. We note the similarity of findings between the AM ER stress proteins (Fig. 7) and the 4-HNE modified lung proteins (Fig. 6) in RAGE null mice: control levels are elevated as compared with WT animals without further change with CS exposure. This may reflect enhanced cellular stress in the unchallenged RAGE null lung, and the bidirectional relationship between oxidative stress and ER stress^43^. These findings also parallel the enlarged airspaces in RAGE null mice as compared with WT mice without further enlargement following CS exposure (Supplementary Fig. 1 and others^22,23^). While the basis for the enlarged airspaces in control RAGE null mice was not determined, our findings are consistent with the observation that lungs from RAGE null mice have increased dynamic lung compliance and lower mRNA and protein levels of elastin as compared with controls^44^.

Gene expression changes mediated by NF-κB in response to CS exposure were attenuated in AM from RAGE null mice. AGE engagement of RAGE results in oxidative stress and subsequent NF-κB activation^45^. Exposure of macrophages to oxidative stress activates NF-κB and consequent cytokine production^46^, suggesting that reduced oxidative stress and attenuated NF-κB signaling might reflect common mechanisms in AM from RAGE null mice. ER stress also increases NF-κB activity^47,48^. The inability of CS to further increase ER stress in RAGE null AM might also contribute to blunted NF-κB -mediated signaling in these cells. Cytokines including TNF-α and IL-1β increase NF-κB mediated transcription and, in turn, are increased by NF-κB -mediated mechanisms. These cytokines are crucial for CS-induced pulmonary emphysema^49^. CS-induced patterns of gene expression mediated by both cytokines were reduced in RAGE null AM as compared with those from WT mice. Greater IFN-γ mediated gene expression also was observed in CS-exposed WT AM as compared with those from RAGE null mice. IFN-γ production is thought integral to lymphocyte-mediated lung destruction in emphysema^50,51^. Collectively, these findings indicate that AM from RAGE null mice exposed to CS for 7 days were not activating patterns of gene expression associated with innate immune mediators with demonstrated relevance to the pathogenesis of CS-induced emphysema.

After 4 months of CS exposure AM from WT mice expressed more genes regulated by Nrf2 than those from RAGE nulls, implying persistent differences in oxidative stress between these groups of mice. In contrast, the expression of genes regulated by XBP1 was no longer increased in AM from chronically smoked WT mice. This suggests that these cells had either generated an adequate compensatory UPR or that the initial ER stress had resolved. The AM from control RAGE-null mice appear to express more genes mediated by XBP1 than their WT counterparts with attenuation by prolonged CS exposure (Fig. 3d). This suggests that the enhanced cellular stress in the RAGE null lung persists with aging and is distinct from those stresses caused by CS exposure.

Of note, AM from both WT and RAGE-null mice displayed lower expression of genes relevant to cytokine activity following prolonged CS exposure compared with age-matched controls (Table 2). Perhaps with prolonged exposure to CS the inflammatory effects of CS exposure are offset by the anti-inflammatory capabilities of nicotine^52,53^, carbon monoxide^54^, or other adaptive changes. Previous microarray studies evaluating AM expression in patients with COPD also show a reprograming of AM as a consequence of chronic smoke exposure with decreased inflammatory and immune response signaling in AM from smokers^55^, and further decrease in patients with COPD^55,56^. The decrement in inflammatory gene expression by chronic CS exposure was more pronounced in AM from RAGE null mice. One could speculate this reflects the absent pro-inflammatory RAGE-ligand signaling.

Expression of RAGE in the unchallenged lung is thought to be confined to the type I cells of the alveolar epithelium^57,58^. We, and others^23^, observe modest RAGE immunostaining of AM in murine tissue sections following prolonged CS exposure. The basis for a discrepancy between this observation and the undetectable RAGE expression of AM obtained by BAL is unclear. It is possible that RAGE expression in AM removed by BAL may differ from other populations of lung macrophages^59^. Alternatively, macrophages are able to bind sRAGE to their surface^60^, thus providing a potential explanation for RAGE immunostaining in the absence of cellular expression. Our findings suggest that AM gene expression patterns associated with accepted mechanisms of emphysema requires a RAGE-dependent signal originating from elsewhere. Possibilities include the alveolar epithelium, sRAGE, or other lung cells with induced RAGE expression. This is consistent with the observation that irradiated RAGE null mice rescued with WT bone marrow are protected from elastase-induced pulmonary emphysema^61^.

A strength of this paper is the comprehensive unbiased experimental approach. The transcriptome of AM exposed to CS *in vivo* has been interrogated using microarray^55,62^. To our knowledge, this is the first use of RNA sequencing to address AM transcriptome responses to *in vivo* CS exposure. The approach provides distinct information from that of microarray given its’ ability to detect RNA species not commonly covered by available arrays^63^ as well as the larger range of expression levels over which RNA species can be detected^64^. In addition, this is the first time that the effect of RAGE deletion on global gene expression has been assessed in any tissue. The results demonstrate that RAGE is central to patterns of AM gene expression likely relevant to the pathogenesis of lung disease due to CS exposure. A third strength is the first demonstration of RAGE inhibition preventing the development of emphysema, which presents an attractive therapeutic possibility for treatment of COPD progression.

The investigation has limitations. AM were sampled by whole lung lavage. Although this is an accepted means of sampling the cellular components of the alveolar compartment, we are uncertain whether the characteristics of macrophages removed by this manner reflect those of all populations of macrophages in the lung. AM are sessile and adherent to the alveolar epithelium, and the extent that BAL removes these macrophages is uncertain^59,65^. The basis for the increased numbers of AM in RAGE null mice exposed to CS for 4 months remains unclear. Perhaps this reflects reduced apoptosis in the cells^22^ due to attenuated oxidative stress^66^.

**Figure 9:**
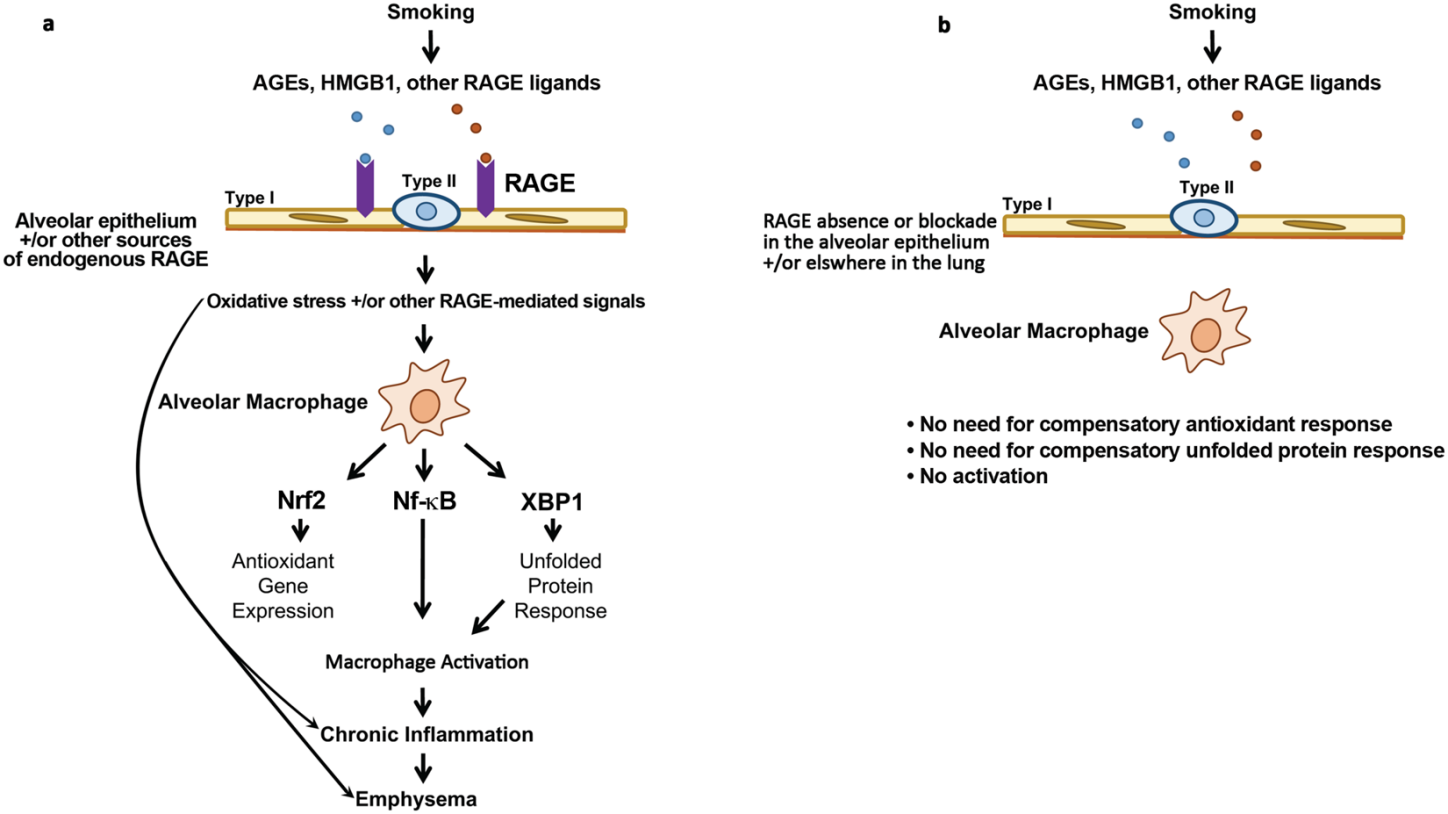
Proposed model for the role of RAGE in CS-mediated lung disease. (**a**) RAGE ligands contained in CS and generated *in vivo* as a result of CS exposure bind to RAGE constitutively expressed by the alveolar epithelium, sRAGE, and/or RAGE induced on lung cells other than AM. This results in oxidative stress. Oxidative stress and/or other RAGE-dependent signals prompt compensatory responses (gene expression patterns associated with Nrf2 and XBP1 activation) and AM activation (gene expression patterns mediated by NF-κB-and cytokines). RAGE mediated disturbances in lung parenchyma and/or AM in response to CS ultimately results in emphysema. (**b**) Attenuated oxidative stress and/or other RAGE-dependent signals in response to CS in the RAGE null mice obviates the need for gene expression patterns mediated by Nrf2 and XBP1 in AM. These cells also have attenuated activation of pathways mediated by NF-κB and cytokines relevant to the pathogenesis of emphysema. The absence of RAGE mediated disturbances in lung parenchyma and/or AM in response to CS prevents the development of emphysema.

In summary, we propose a paradigm shown in Fig. 9. RAGE ligands contained in CS and generated *in vivo* as a result of CS exposure bind to RAGE constitutively expressed by the alveolar epithelium, sRAGE, and/or RAGE induced on lung cells other than AM. This results in oxidative stress. Oxidative stress and/or other RAGE-dependent signals prompt compensatory responses (gene expression patterns associated with Nrf2 and XBP1 activation) and AM activation (gene expression patterns mediated by NF-κB-and cytokines). RAGE mediated disturbances in lung parenchyma and/or AM in response to CS ultimately result in emphysema. Attenuated oxidative stress and/or other RAGE-dependent signals in response to CS by RAGE mutation or inhibition obviate the need for gene expression patterns mediated by Nrf2 and XBP1 in AM. These cells also have attenuated activity of pathways mediated by NF-κB and cytokines relevant to the pathogenesis of emphysema as compared with WT AM. Given the evidence that RAGE plays a role in development of COPD in humans, future work should clarify the relevant cellular source(s) of RAGE, the relationship between RAGE and the source(s) of CS-induced oxidative stress, and the mechanism(s) whereby RAGE-expressing cells and/or sRAGE communicate with AM in response to CS. It will also be important to determine whether RAGE blockade attenuates oxidative stress, ER stress and AM activation in response to CS-exposure in human lungs. The attenuation of airspace enlargement by treatment of CS-exposed AKR mice with FPS-ZM1 suggests such a strategy could be therapeutically viable and might limit lung damage due to CS exposure in patients unable to quit smoking.

## Methods

### Statement on animal welfare

Animal use and husbandry followed protocols approved by the Salt Lake City Veterans Administration Institutional Animal Care and Use Committee.

### Animal exposures and sample collection

C57BL/6 (WT) mice were originally obtained from The Jackson Laboratory and maintained as a colony within our facility. Breeding pairs of RAGE null mice on a C57BL/6 background were initially a gift from Dr. Peter Nawroth^67^ and maintained as a colony within our facility. AKR mice were obtained from The Jackson Laboratory. AKR mice were only used for experiments assessing the effects of the RAGE inhibitor FPS-ZM1 on lung morphometry and BAL cell counts following 2 months of exposure to CS or control conditions. All other experiments were performed on C57BL/6 mice with or without homozygous null mutation of the gene expressing RAGE. All CS-exposed and control mice were age and gender matched. All mice were 8–12 weeks of age at the initiation of experiments. Female mice were used for all experiments in which emphysema was quantified by lung morphometry given the observation that female mice may be more prone to develop emphysema with CS exposure than male mice^68^. The effects of CS exposure on AM gene expression was compared between male WT and RAGE null mice. Mice were exposed to CS using a Teague Model TE-10 (Teague Enterprises, Woodland, CA) smoking machine, which produces a combination of side-stream and mainstream CS. A pump on the machine “puffs” each 3R4F University of Kentucky research cigarette for 2 seconds for a total of 9 puffs before ejection. The exposure was over 2.5 hours for 7 consecutive days (WT and RAGE null mice), 5 days/week for 4 months (WT and RAGE null mice), or 5 days/week for 2 months (AKR mice). The smoking chamber atmosphere was periodically sampled to confirm total particulate matter concentrations of approximately 150 mg/m^3^. Mice were weighed at the start of exposure and weekly throughout the duration of smoke exposure. FPS-ZM1 was administered by intraperitoneal injection to female AKR mice at a dose of 1.0 mg/kg on days of smoke exposure.

### Lung morphometry

At 2 (AKR/J) or 4 months (WT or RAGE null) of exposure, CS exposed or control female mice had measurement of lung volume and quantitation of emphysema. At the specified times, mice were administered an overdose of intraperitoneal tribromoethyl alcohol (Avertin, Aldrich Chemical Co.) at a minimum dose of 0.34 mg/g body weight, euthanized by exsanguination and the lungs were excised. The trachea was cannulated. The volume of lungs inflated to 25 cm pressure was measured by water displacement. After measurements the lungs were inflation fixed, processed and sectioned and stained with hematoxylin and eosin. Four nonconsecutive slides per mouse were coded and analyzed (magnification of x100). Airspace enlargement was quantified by measuring the mean linear intercept (L_m_) in 20 randomly selected fields per slide^26,27^.

### BAL

The chest was opened and the trachea isolated as described above. BAL of both lungs of mice was performed with four 0.8 ml aliquots of PBS/EDTA. Cells were recovered from the lavage fluid by centrifugation at 400 g for ten minutes and re-suspended in PBS. The total number of recovered cells was determined using a Scepter Cell Counter (Millipore). An aliquot of cells was cytospun onto glass microscope slides, air-dried and stained with Wright-Giemsa for determination of cell differentials. The remaining cells were combined for RNA and protein isolation. Post-BAL lung tissue also was used for RNA and protein isolation. A subset of animals had lungs removed immediately following euthanasia for fluorescence-activated cell sorting (FACS) analysis.

### RNA isolation

Total RNA was isolated from pelleted AM or lung tissue using a Qiagen RNeasy mini kit. After RNA extraction, total RNA was quantified by spectrophotometry and the quality of RNA determined using an Agilent bioanalyzer. AM samples with total RNA integrity values ≥ 7 were used for RNA sequencing.

### RNA sequencing

One μg total RNA from AM recovered by BAL from 3 WT and 4 RAGE null male control mice, and 4 WT and 4 RAGE null male mice exposed to CS for 7 days was used for Illumina TruSeq library preparation with oligo dT selection (15 samples). PCR amplified cDNA libraries were prepared and sequenced using single-end 50 base pair read chemistry on an Illumina HiSeq. 2000 instrument. At 4 months 1 μg total RNA from recovered AM of 4 WT and 4 RAGE null control mice, and 4 WT and 4 RAGE null mice exposed to CS was similarly used for library preparation and sequencing (16 samples).

After filtering out reads that align to more than one genomic location and reads with more than 4 base pair mismatches, there were between 11.7 and 20.0 million high quality reads in the 7 day samples (mean 14.8 million per sample) and between 12.8 and 20.0 million reads in the 4 month samples (mean 17.6 million per sample). The AM RNA-Seq datasets were deposited into the NCBI Gene Expression Omnibus (GEO) database under accession number GSE75513.

### Bioinformatics analysis

#### Differential gene expression

Illumina read files (fastq format) were aligned to the mouse Mm10 reference genome Novoalign software (Novocraft 2.08) and converted to binary BAM files using the Useq Sam Transcriptome Parser application (Useq. 8.7)^69^. Differentially expressed genes were determined using the Useq DefinedRegionsDifferentialSeq (DRDS) application that makes use of the DESeq package to measure the overdispersion between biological replicas and calculates a p-value based on a negative binomial distribution^70^. These p-values are controlled for multiple testing using the Benjamini-Hochberg false discovery method as previously described^71^.

#### Principal component and hierarchical clustering analysis

To identify patterns of genotype and CS-dependent changes in gene expression, differentially expressed genes (fold change ≥ 1.5, FDR < 0.05) as determined by DESeq were evaluated by principal component analysis and hierarchical clustering using Cluster 3.0 software^72^. Eigenvector values for the top three principal components were plotted using the “rgl” 3D application in the statistical programing language R. Heatmaps of differentially expressed genes were visualized using Java Treeview^73^.

#### Biological pathway analysis

Ingenuity^®^ pathway analysis (IPA) software was used to identify biological pathways and disease processes represented in differentially expressed genes. Four different comparisons were made: 1) control WT mice compared to control RAGE null mice, 2) CS-exposed WT mice to control WT mice, 3) CS-exposed RAGE null mice to control RAGE null mice, and 4) CS-exposed WT mice to CS-exposed RAGE null mice. Using IPA upstream regulator analysis we determined the statistically significant (Fisher exact test) overlap between differentially expressed genes and known transcription factor and/or cytokine target genes. A z-score that infers the activation state of an upstream regulator based on the direction and fold change of its target genes was also determined^74^. A z-score ≥ 2 or ≤ −2 was considered statistically significant.

### Quantitative PCR

RNA expression of thioredoxin reductase 1 (Txnrd1), heat shock 105 kDa/110 kDa protein 1 (Hsph1), tumor necrosis factor (Tnf), and RAGE (Ager) was evaluated by quantitative reverse transcription PCR (qPCR). First-strand cDNA was reverse-transcribed from total RNA using a High Capacity cDNA Reverse Transcription Kit (Life Technologies). The qPCR was performed using TaqMan Gene Expression Assays supplied by Life Technologies on an ABI 7500 Real-Time PCR System. The cDNA was mixed with TaqMan Gene Expression Master Mix and the appropriate primers for the genes of interest. The comparative cycle threshold (CT) method (2^−∆∆CT^) was used to calculate relative gene expression under experimental and control conditions normalized to beta-actin (*ACTB*).

### Western blotting

Recovered AM and post-BAL lung tissue from control or 7-day CS-exposed WT or RAGE null mice were lysed in buffer (50 mm Tris, pH 8.0, 1% Nonidet P-40, 150 mm NaCl, 0.5% sodium deoxycholate, 0.1% SDS) containing protease inhibitors (Thermo Fisher Scientific, #78410). Lysates were pooled from 3 to 4 mice per group, and 30 μg of protein was resolved by SDS-PAGE and transferred to nitrocellulose membranes according to standard procedures. Membranes were blocked, incubated with primary antibodies followed by horseradish peroxidase-conjugated corresponding secondary antibody or IRDye (LI-COR, #925-68023, #926-32214) incubation. Antibody binding was visualized with SuperSignal West Pico Chemiluminescent Substrate (Thermo Fisher Scientific, #34080) or Odyssey CL Imaging System (LI-COR), according to the manufacturer’s protocol. Antibodies against PERK (Cell Signaling, #3192), IRE1α (Cell Signaling, #3294), PDIA3 (Cell Signaling, #3501), RAGE (R&D Systems, #AF1179), and 4-hydroxynonenal (4-HNE) (EMD Millipore, #AB5605) were used for western blots. Equal loading of protein was verified with beta-actin (Santa Cruz Biotechnology, #SC1616R).

### Immunohistochemistry

Formalin-fixed and paraffin embedded lung slides (5 μm) were incubated with a polyclonal goat anti RAGE antibody (AF1179, R&D systems, Minneapolis, MN, USA) and visualized by an impact DAB visualizing kit (SK 4105, Vector lab, Burlingame, CA, USA).

### Quantification of nuclear transcription factors

Nuclear protein extracts were prepared following manufacturer’s protocol (FIVEphoton Biochemicals). In several cases, the AM from 2 mice of similar exposure were combined to provide adequate numbers of cells to allow nuclear for protein extraction. A total of 3 samples per genotype/treatment was prepared. Nuclear protein extracts were added to wells containing double-stranded DNA molecules with a NF-κB response element. Subsequent protein detection with a primary antibody directed against p65 was performed and quantified according to manufacturer’s protocol (NFkB p65 Transcription Factor Assay Kit, Abcam). Nuclear protein extracts were assessed for Nrf2 presence using a similar approach according to manufacturer’s protocol (RayBio® Mouse Nrf2 Transcription Factor Activity Assay Kit, RayBiotech).

### Fluorescence activated cell sorting

Lung cell suspensions from male and female WT and RAGE null mice exposed to either 7 days of CS or control conditions were obtained using the Miltenyi Biotec lung dissociation mouse kit (lot.5160801183) using gentle MACs dissociator (Milte)nyi Biotec Ltd. CD45^+^ cells were selected from other lung cells. These cells were labeled with fluorescence conjugated antibodies (BioLegend, cat.101212, cat.1276117) and samples were run using the BD FACSCanto II (BD Biosciences). Data were analyzed using FlowJo software (TreeStar, USA). Neutrophils subset were identified as CD11b^+^ Ly-6G^+^ ^59^.

### Statistical analysis

Unless otherwise stated in the figure legends, statistical differences between groups were determined by Student’s T test or one-way ANOVA using GraphPad PRISM. Error bars reflect the standard error of the mean.

## Electronic supplementary material

Supplementary Material

## Data Availability

The AM RNA-Seq datasets were deposited into the NCBI GEO as described above. The remainder of data generated and analyzed during this study are included in this article and accompanying supplementary files.
