# Supplementary material for: RAGE is a Critical Mediator of Pulmonary Oxidative Stress, Alveolar Macrophage Activation and Emphysema in Response to Cigarette Smoke
Source: Sci Rep. 2019 Jan 18;9:231. doi: 10.1038/s41598-018-36163-z (PMC6338799; doi:10.1038/s41598-018-36163-z)
Supplement: Supplementary file 1 — Supplementary Material [file 41598_2018_36163_MOESM1_ESM.pdf]

Supplementary Material

RAGE is a Critical Mediator of Pulmonary Oxidative Stress, Alveolar Macrophage Activation and Emphysema in Response to Cigarette Smoke

Karl A. Sanders[1,4], Don A. Delker[2], Tom Huecksteadt[4], Emily Beck[1,4], Tanna Wuren[4], Yuntian Chen[5], Yuxia Zhang[6], Mark W. Hazel[2] and John R. Hoidal[1,3,4]

[1]Division of Respiratory, Critical Care, and Occupational Pulmonary Medicine, University of Utah, Salt Lake City, Utah. [2]Division of Gastroenterology, Hepatology, and Nutrition, University of Utah, Salt Lake City, Utah. [3]Department of Internal Medicine, University of Utah, Salt Lake City, Utah. [4]George E. Wahlen Department of Veterans Affairs Medical Center, Salt Lake City, Utah. [5]Shanghai Ninth People's Hospital, Shanghai Jiao Tong University School of Medicine, Shanghai, China. [6]Department of Pharmacology, Toxicology and Therapeutics, University of Kansas Medical Center, Kansas City, Kansas.

Corresponding author J.R.H (email: John.Hoidal@hsc.utah.edu)

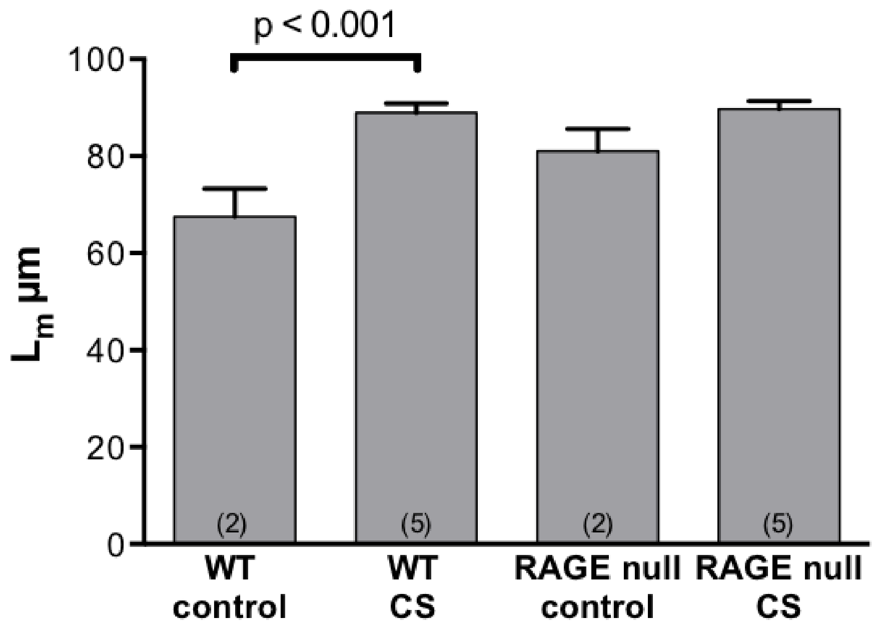

Supplementary Figure 1. RAGE null mice are protected from tissue destruction following 4 months CS exposure. L_m values demonstrate increased volume-to-surface ratios in RAGE null mice as compared with WT controls. Four months of CS exposure only increases L_m values in WT mice. Number of mice per group is indicated in parentheses. Statistical significance determined by one-way ANOVA.

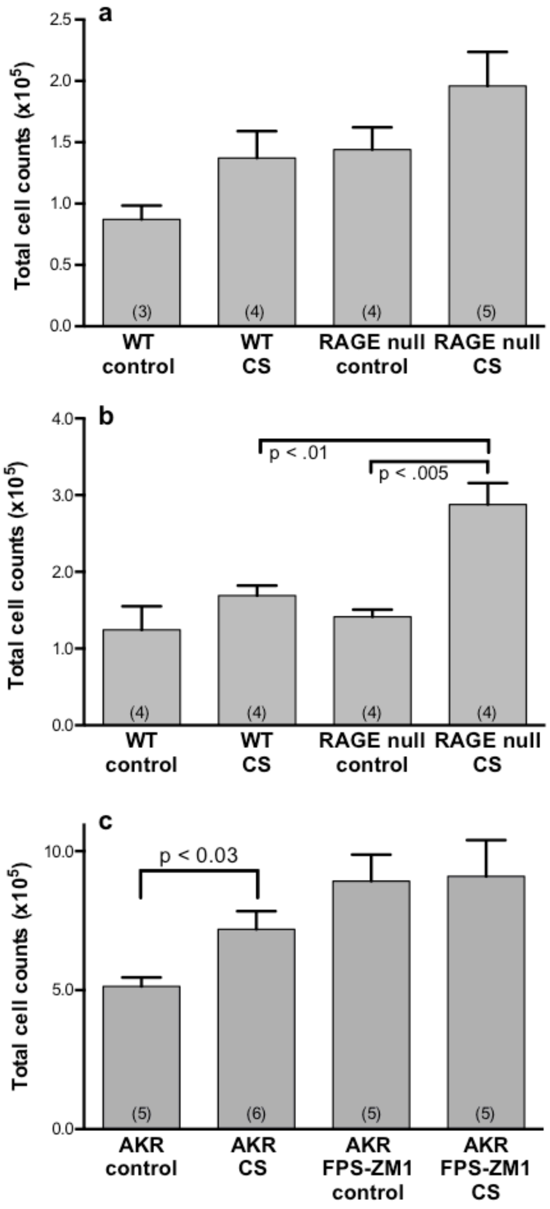

Supplementary Figure 2. Absence or inhibition of RAGE does not reduce bronchoalveolar lavage cell counts following acute or chronic CS exposure. (a) Neither WT nor RAGE null mice had a statistically significant increase in BAL cell counts after 7 days of CS exposure, although clear trends were present in both groups. **(b)** After 4 months of CS exposure, only smoked RAGE null mice had significantly increased numbers of cells in BAL. **(c)** After 2 months of CS exposure the AKR mice had significantly increased BAL cell counts as compared with controls. Administration of FPS-ZM1 to AKR mice significantly increased BAL cells counts as compared with untreated controls. However, there was no additional increase in BAL cell counts following two months of CS exposure in the FPS-ZM1 treated AKR mice as compared with FPS-ZM1 treated controls. Graphs show mean and standard error for each condition and/or genotype. Number of mice per group is indicated in parentheses. Statistical differences were determined by one-way ANOVA.

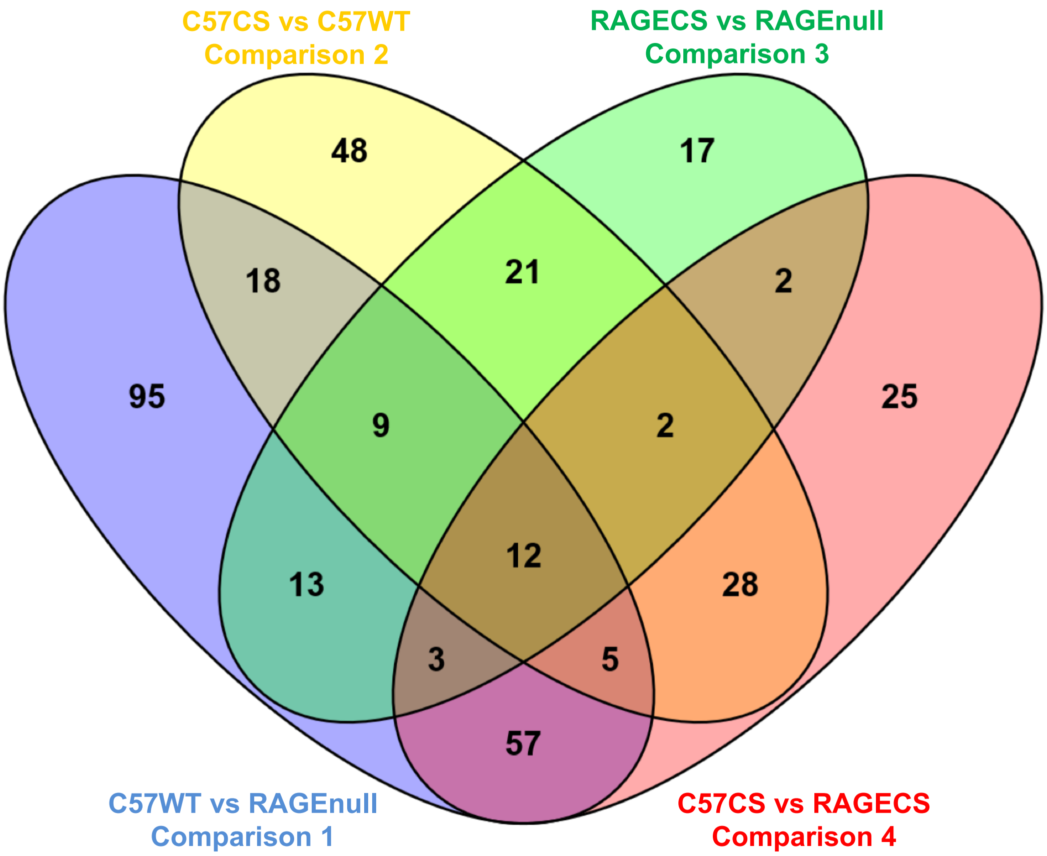

Supplementary Figure 3. RNA sequencing demonstrates that CS exposure and RAGE mutation impact AM gene expression. 212, 143, 79 and 134 genes were differentially expressed (Fold ≥ 1.5, FDR < 0.05) for control WT mice compared to control RAGE null mice (Comparison 1), WT mice exposed to 7 days CS compared to control WT mice (Comparison 2), RAGE null mice exposed to 7 days CS compared to control RAGE null mice (Comparison 3) and WT mice exposed to 7 days CS compared to RAGE null mice exposed to 7 days CS (Comparison 4), respectively.

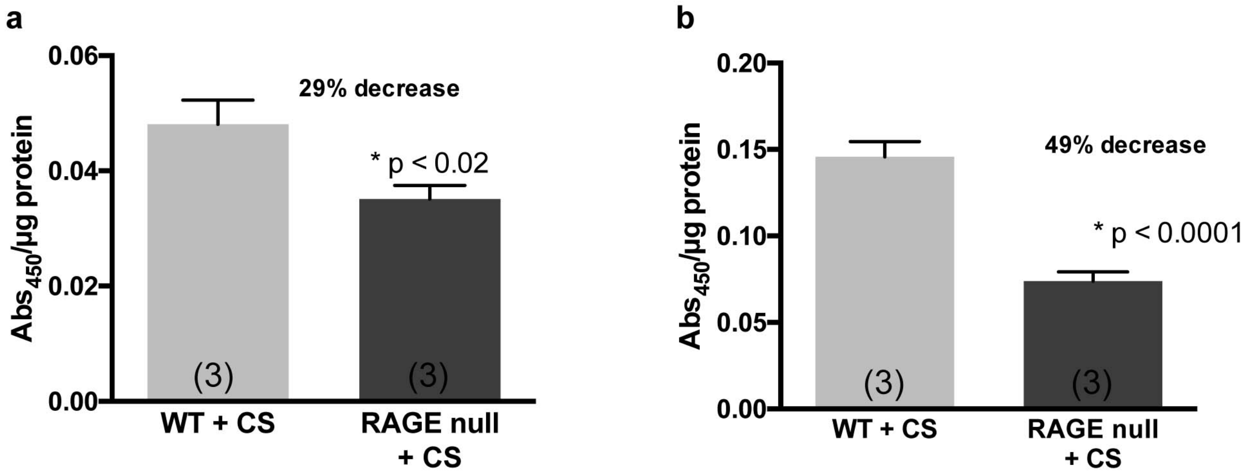

Supplementary Figure 4. Nuclei from AM of RAGE null mice have less NF-κB and Nrf2 protein than WT mice following 7 days of CS exposure. (a) The AM nuclei from RAGE null mice have 29% less NF–κB (p65) protein than WT mice after 7 days CS exposure ($p < 0.02$). **(b)** The AM nuclei from RAGE null mice have 49% less Nrf2 protein than WT mice after 7 days CS exposure ($p < 0.0001$). Number of mice per group is indicated in parentheses. Statistical differences were determined by Student's t-test.

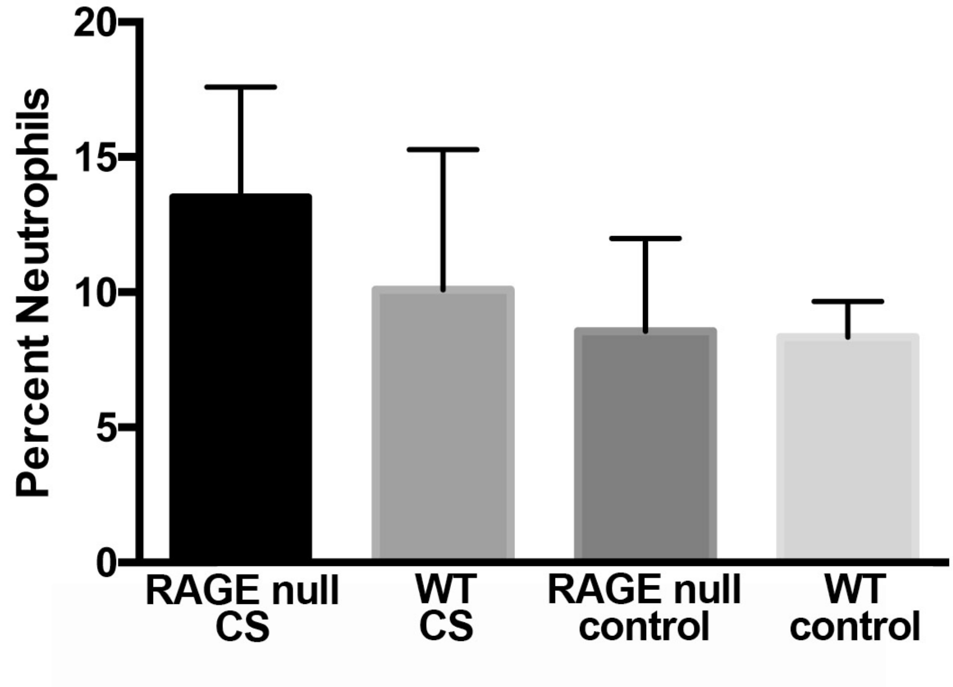

Supplementary Figure 5. Absence of CS-induced oxidative stress in the lungs of RAGE null mice as compared with WT lungs is not due to fewer neutrophils. The percentage of CD45 positive cells with a neutrophil phenotype as defined by cells that co-labeled with anti-Ly6G and anti-CD11b antibodies was measured by FACS. There was no difference in percentages of neutrophils between WT and RAGE null mice following 7 days of CS exposure. Three WT mice were exposed to CS, the rest of the groups consisted of 4 mice.

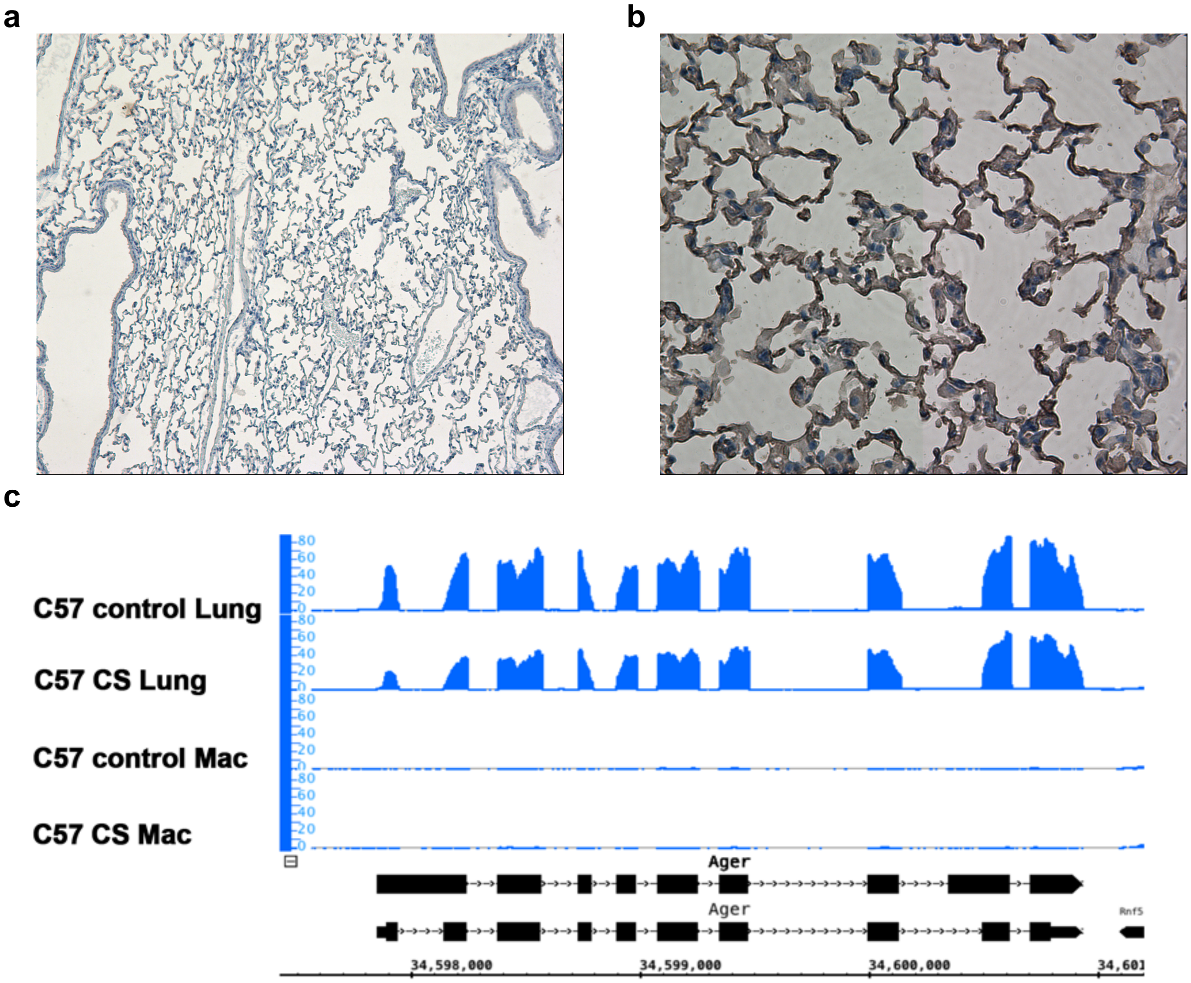

Supplementary Figure 6. RAGE is expressed in lung parenchyma and not AM following prolonged CS exposure. (a) Immunostaining of control RAGE null tissue with anti-RAGE antibodies demonstrates absence of stain and (therefore) the specificity of the antibody. **(b)** Lung tissue from WT mice exposed to CS for 4 months and stained with anti-RAGE antibodies demonstrates prominent RAGE expression in the alveolar septum. Cells within the alveoli with AM morphology also faintly immunostain for RAGE. **(c)** RNA sequencing data demonstrates minimal RAGE expression in AM exposed to 4 months CS. In contrast, RAGE is expressed in post-lavage lung tissue from these same mice.

Figure 7

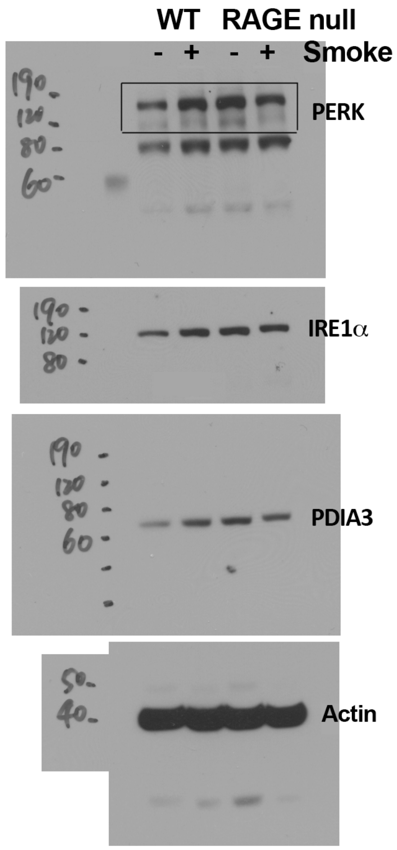

Figure 8

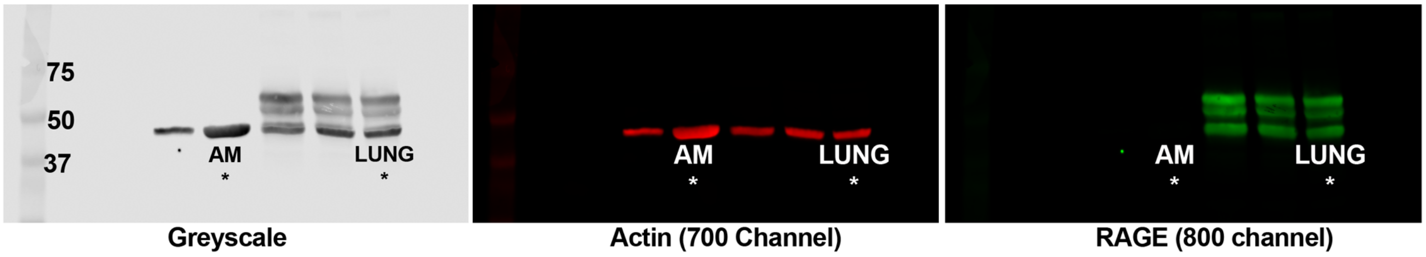

Supplementary Figure 7. Complete western blot gels. Western blot gels including protein loading references (β-actin) are shown for data presented in Figure 7 (PERK, IRE1α, and PDIA3), and Figure 8b (RAGE).

Supplementary Table 1.

	Description	Symbol	WT-CTRL/RAGE null-CTRL (Fold)	WT-CTRL/RAGE null-CTRL (FDR)	WT-CS/WT-CTRL (Fold)	WT-CS/WT-CTRL (FDR)	RAGE null-CS/RAGE null-CTRL (Fold)	RAGE null-CS/RAGE null-CTRL (FDR)	WT-CS/RAGE null-CS (Fold)	WT-CS/RAGE null-CS (FDR)
ENSMUSG00000024125	spermine binding protein-like [Sour	Sbpl	0.728	0.000	5.135	0.000	0.795	0.000	4.705	0.000
ENSMUSG00000047517	deleted in malignant brain tumors 1	Dmbt1	0.786	0.000	3.273	0.000	0.675	0.000	3.814	0.000
ENSMUSG00000024768	lipase, gastric [Source:MGI Symbol;	Lipf	0.935	0.266	3.502	0.000	0.943	0.552	3.472	0.000
ENSMUSG00000057417	demilune cell and parotid protein 3	Dcpp3	0.763	0.000	3.704	0.000	0.886	0.000	3.189	0.000
ENSMUSG00000047228	cDNA sequence BC048546 [Source:	BC048546	0.829	0.000	2.876	0.000	0.801	0.000	2.974	0.000
ENSMUSG00000032496	lactotransferrin [Source:MGI Symbo	Ltf	0.851	0.036	2.350	0.000	0.554	0.000	3.609	0.000
ENSMUSG00000052305	hemoglobin, beta adult major chain	Hbb-b1	1.763	0.000	0.197	0.000	0.372	0.000	0.935	0.000
ENSMUSG00000015519	RIKEN cDNA 2310057J18 gene [Sour	310057J18Ri	1.000	1.000	2.949	0.000	1.027	1.000	2.871	0.000
ENSMUSG00000069919	hemoglobin alpha, adult chain 1 [So	Hba-a1	1.546	0.000	0.272	0.000	0.494	0.000	0.851	0.000
ENSMUSG00000073940	hemoglobin, beta adult minor chain	Hbb-b2	1.486	0.000	0.342	0.000	0.606	0.000	0.838	0.000
ENSMUSG00000022389	thyrotroph embryonic factor [Sourc	Tef	1.420	0.000	3.855	0.000	5.874	0.000	0.932	0.765
ENSMUSG00000069917	hemoglobin alpha, adult chain 2 [So	Hba-a2	1.528	0.000	0.346	0.000	0.574	0.000	0.922	0.003
ENSMUSG00000059824	D site albumin promoter binding pr	Dbp	1.111	0.089	3.341	0.000	3.381	0.000	1.097	0.573
ENSMUSG00000067843			0.826	0.000	2.205	0.000	0.799	0.000	2.279	0.000
ENSMUSG00000031785	G protein-coupled receptor 56 [Sou	Gpr56	0.214	0.000	1.981	0.000	0.775	0.018	0.548	0.000
ENSMUSG00000090877	heat shock protein 1B [Source:MGI	Hspa1b	1.707	0.000	3.221	0.000	1.619	0.000	3.396	0.000
ENSMUSG00000000983	WAP four-disulfide core domain 18	Wfdc18	0.927	0.180	1.851	0.000	0.935	0.565	1.837	0.000
ENSMUSG00000029657	heat shock 105kDa/110kDa protein	Hsph1	0.762	0.001	2.731	0.000	1.297	0.020	1.604	0.000
ENSMUSG00000025270	aminolevulinic acid synthase 2, eryt	Alas2	1.328	0.000	0.488	0.000	0.682	0.000	0.950	0.065
ENSMUSG00000020889	nuclear receptor subfamily 1, group	Nr1d1	1.101	0.303	2.624	0.000	2.158	0.000	1.339	0.003
ENSMUSG00000091971	heat shock protein 1A [Source:MGI	Hspa1a	1.929	0.000	3.062	0.000	1.815	0.000	3.253	0.000
ENSMUSG00000030954	glycoprotein 2 (zymogen granule m	Gp2	0.826	0.000	1.997	0.000	0.987	0.955	1.670	0.000
ENSMUSG00000027737	solute carrier family 7 (cationic ami	Slc7a11	2.579	0.000	2.557	0.000	2.854	0.000	2.310	0.000
ENSMUSG00000021775	nuclear receptor subfamily 1, group	Nr1d2	1.191	0.029	2.399	0.000	2.451	0.000	1.166	0.216
ENSMUSG00000030142	C-type lectin domain family 4, mem	Clec4e	1.532	0.000	2.411	0.000	2.287	0.000	1.615	0.000
ENSMUSG00000060586	histocompatibility 2, class II antigen	H2-Eb1	1.767	0.000	0.441	0.000	0.567	0.000	1.373	0.000
ENSMUSG00000021187	tandem C2 domains, nuclear [Sourc	Tc2n	0.538	0.000	2.354	0.000	1.556	0.000	0.814	0.046
ENSMUSG00000015656	heat shock protein 8 [Source:MGI S	Hspa8	0.629	0.000	2.290	0.000	1.148	0.570	1.256	0.005

ENSMUSG00000064057	secretoglobin, family 3A, member 1	Scgb3a1	0.729	0.000	2.330	0.000	0.926	0.970	1.834	0.000
ENSMUSG00000055866	period homolog 2 (Drosophila) [Sou	Per2	0.971	0.823	2.101	0.000	3.039	0.000	0.671	0.000
ENSMUSG00000036594	histocompatibility 2, class II antigen	H2-Aa	1.795	0.000	0.454	0.000	0.575	0.000	1.417	0.000
ENSMUSG00000092609	predicted gene 20481 [Source:MGI	Gm20481	1.657	0.000	2.511	0.000	1.600	0.000	2.601	0.000
ENSMUSG00000038508	growth differentiation factor 15 [So	Gdf15	1.112	0.220	2.153	0.000	1.467	0.000	1.632	0.000
ENSMUSG00000028957	period homolog 3 (Drosophila) [Sou	Per3	1.368	0.000	2.170	0.000	3.003	0.000	0.988	1.000
ENSMUSG00000073421	histocompatibility 2, class II antigen	H2-Ab1	1.676	0.000	0.481	0.000	0.607	0.000	1.329	0.000
ENSMUSG00000047959	potassium voltage-gated channel, s	Kcna3	1.467	0.000	0.527	0.000	1.330	0.003	0.581	0.000
ENSMUSG00000056749	nuclear factor, interleukin 3, regulat	Nfil3	0.919	0.482	0.511	0.000	0.594	0.000	0.790	0.001
ENSMUSG00000030144	C-type lectin domain family 4, mem	Clec4d	1.710	0.000	2.124	0.000	1.968	0.000	1.846	0.000
ENSMUSG00000026390	macrophage receptor with collagen	Marco	2.137	0.000	2.045	0.000	1.885	0.000	2.318	0.000
ENSMUSG00000024610	CD74 antigen (invariant polypeptide	Cd74	1.613	0.000	0.493	0.000	0.568	0.000	1.400	0.000
ENSMUSG00000022096	hairless [Source:MGI Symbol;Acc:M	Hr	0.881	0.261	0.516	0.000	0.629	0.000	0.723	0.000
ENSMUSG00000005547	cytochrome P450, family 2, subfami	Cyp2a5	0.862	0.114	2.145	0.000	1.296	0.001	1.426	0.000
ENSMUSG00000015837	sequestosome 1 [Source:MGI Symb	Sqstm1	1.010	0.995	1.968	0.000	1.228	0.141	1.618	0.000
ENSMUSG00000026864	heat shock protein 5 [Source:MGI S	Hspa5	0.646	0.000	1.968	0.000	0.867	0.283	1.466	0.000
ENSMUSG00000063011	mesothelin [Source:MGI Symbol;Ac	Msln	0.821	0.027	1.607	0.000	0.728	0.000	1.814	0.000
ENSMUSG00000005413	heme oxygenase (decycling) 1 [Sour	Hmox1	2.033	0.000	2.036	0.000	1.582	0.000	2.617	0.000
ENSMUSG00000068735	transformation related protein 53 in	Trp53i11	0.755	0.003	0.566	0.000	0.797	0.007	0.535	0.000
ENSMUSG00000021270	heat shock protein 90, alpha (cytoso	Hsp90aa1	0.895	0.210	1.935	0.000	1.325	0.002	1.308	0.000
ENSMUSG00000025150	carbonyl reductase 2 [Source:MGI S	Cbr2	0.769	0.002	2.009	0.000	1.245	0.014	1.242	0.040
ENSMUSG00000093327	microRNA 5107 [Source:MGI Symbo	Mir5107	1.568	0.000	0.537	0.000	0.623	0.000	1.351	0.000
ENSMUSG00000030017	regenerating islet-derived 3 gamma	Reg3g	0.665	0.000	1.900	0.000	0.848	0.648	1.490	0.000
ENSMUSG00000073902	predicted gene 1966 [Source:MGI S	Gm1966	1.535	0.000	0.593	0.000	1.309	0.001	0.695	0.000
ENSMUSG00000022769	stromal cell-derived factor 2-like 1 [	Sdf2l1	0.696	0.000	1.843	0.000	1.035	0.975	1.238	0.017
ENSMUSG00000038791	secretoglobin, family 3A, member 2	Scgb3a2	0.824	0.022	1.925	0.000	1.086	0.615	1.461	0.000
ENSMUSG00000074280	predicted gene 6166 [Source:MGI S	Gm6166	1.139	0.844	1.854	0.000	0.555	0.000	3.803	0.000
ENSMUSG00000023272	cysteine-rich with EGF-like domains	Creld2	0.705	0.000	1.780	0.000	1.002	0.992	1.253	0.006
ENSMUSG00000020836	coronin 6 [Source:MGI Symbol;Acc:	Coro6	1.583	0.000	0.578	0.000	1.139	0.658	0.803	0.007
ENSMUSG00000018581	dynein, axonemal, heavy chain 11 [S	Dnahc11	1.639	0.000	0.568	0.000	0.912	0.675	1.020	0.966
ENSMUSG00000089901	predicted gene 8113 [Source:MGI S	Gm8113	1.334	0.001	0.561	0.000	1.066	0.938	0.703	0.000
ENSMUSG00000024190	dual specificity phosphatase 1 [Sour	Dusp1	1.933	0.000	0.582	0.000	0.942	0.989	1.195	0.267
ENSMUSG00000025351	CD63 antigen [Source:MGI Symbol;	Cd63	0.951	0.720	1.745	0.000	1.284	0.006	1.293	0.000
ENSMUSG00000037706	CD81 antigen [Source:MGI Symbol;	Cd81	1.055	0.812	1.721	0.000	1.515	0.000	1.199	0.013

ENSMUSG00000028378	prostaglandin reductase 1 [Source:	Ptgr1	1.441	0.000	1.755	0.000	1.434	0.000	1.764	0.000
ENSMUSG00000054422	fatty acid binding protein 1, liver [So	Fabp1	1.165	0.204	0.572	0.000	0.855	0.197	0.778	0.001
ENSMUSG00000022504	class II transactivator [Source:MGI S	Ciita	1.416	0.000	0.613	0.000	0.862	0.051	1.007	1.000
ENSMUSG00000023913	phospholipase A2, group VII (platele	Pla2g7	0.357	0.000	1.693	0.000	1.372	0.003	0.440	0.000
ENSMUSG00000063286	predicted gene 8995 [Source:MGI S	Gm8995	1.727	0.000	0.599	0.000	1.430	0.000	0.723	0.000
ENSMUSG00000026177	solute carrier family 11 (proton-cou	Slc11a1	1.976	0.000	1.787	0.000	2.029	0.000	1.741	0.000
ENSMUSG00000020572	nicotinamide phosphoribosyltransfe	Nampt	1.349	0.000	1.740	0.000	1.485	0.000	1.581	0.000
ENSMUSG00000001025	S100 calcium binding protein A6 (ca	S100a6	0.851	0.143	0.580	0.000	0.578	0.000	0.855	0.048
ENSMUSG00000032802	sulfiredoxin 1 homolog (S. cerevisia	Srxn1	1.736	0.000	1.773	0.000	1.632	0.000	1.886	0.000
ENSMUSG00000001020	S100 calcium binding protein A4 [So	S100a4	1.172	0.105	0.577	0.000	0.545	0.000	1.241	0.015
ENSMUSG00000030413	peptidoglycan recognition protein 1	Pglyrp1	0.871	0.154	1.553	0.000	0.870	0.519	1.555	0.000
ENSMUSG00000039013	sialic acid binding Ig-like lectin 5 [So	Siglec5	0.894	0.365	0.612	0.000	0.655	0.000	0.836	0.020
ENSMUSG00000024298	zinc finger protein 871 [Source:MGI	Zfp871	1.487	0.000	0.626	0.000	1.195	0.150	0.779	0.001
ENSMUSG00000037355	RIKEN cDNA 4933407H18 gene [Sou(	18Ri	1.462	0.000	0.622	0.000	1.108	0.723	0.821	0.020
ENSMUSG00000023944	heat shock protein 90 alpha (cytoso	Hsp90ab1	0.919	0.432	1.668	0.000	1.383	0.000	1.108	0.363
ENSMUSG00000024966	stress-induced phosphoprotein 1 [S	Stip1	0.806	0.006	1.664	0.000	1.204	0.112	1.115	0.362
ENSMUSG00000020250	thioredoxin reductase 1 [Source:MG	Txnrd1	1.399	0.000	1.681	0.000	1.470	0.000	1.599	0.000
ENSMUSG00000032575	mesencephalic astrocyte-derived ne	Manf	0.730	0.000	1.652	0.000	0.860	0.178	1.401	0.000
ENSMUSG00000022126	immunoresponsive gene 1 [Source:	Irg1	1.288	0.001	1.700	0.000	1.551	0.000	1.411	0.000
ENSMUSG00000032115	hypoxia up-regulated 1 [Source:MG	Hyou1	0.702	0.000	1.631	0.000	0.956	0.909	1.197	0.051
ENSMUSG00000026463	ATPase, Ca++ transporting, plasma	Atp2b4	0.733	0.000	0.641	0.000	0.716	0.000	0.656	0.000
ENSMUSG00000019577	pyruvate dehydrogenase kinase, iso	Pdk4	0.535	0.000	0.646	0.000	0.726	0.000	0.476	0.000
ENSMUSG00000025823	protein disulfide isomerase associat	Pdia4	0.755	0.000	1.633	0.000	1.086	0.765	1.134	0.300
ENSMUSG00000036585	fibroblast growth factor 1 [Source:M	Fgf1	1.386	0.000	0.634	0.000	1.000	0.992	0.878	0.070
ENSMUSG00000001774	cysteine and histidine-rich domain (	Chordc1	0.828	0.038	1.668	0.000	1.084	0.751	1.275	0.003
ENSMUSG00000017723	WAP four-disulfide core domain 2 [S	Wfdc2	0.623	0.000	1.679	0.000	0.903	0.833	1.160	0.296
ENSMUSG00000000275	tripartite motif-containing 25 [Sourc	Trim25	1.202	0.022	0.627	0.000	0.953	0.938	0.791	0.002
ENSMUSG00000019916	procollagen-proline, 2-oxoglutarate	P4ha1	0.827	0.017	1.626	0.000	1.181	0.181	1.138	0.221
ENSMUSG00000027435	CD93 antigen [Source:MGI Symbol;	Cd93	1.316	0.000	1.552	0.000	1.146	0.283	1.783	0.000
ENSMUSG00000092528	NLR family, pyrin domain containing	Nlrp1c	1.403	0.000	0.626	0.000	1.003	0.951	0.875	0.169
ENSMUSG00000070369	integrin, alpha D [Source:MGI Symb	Itgad	1.213	0.010	0.650	0.000	0.832	0.039	0.948	0.785
ENSMUSG00000053279	aldehyde dehydrogenase family 1, s	Aldh1a1	0.836	0.055	1.710	0.000	1.006	1.000	1.420	0.000
ENSMUSG00000000982	chemokine (C-C motif) ligand 3 [Sou	Ccl3	1.237	0.004	1.624	0.000	1.291	0.001	1.556	0.000
ENSMUSG00000024892	pyruvate carboxylase [Source:MGI S	Pcx	1.071	0.668	1.554	0.000	1.458	0.000	1.142	0.127

ENSMUSG00000045193	cold inducible RNA binding protein [	Cirbp	1.327	0.000	0.631	0.000	0.928	0.792	0.903	0.393
ENSMUSG00000058427	chemokine (C-X-C motif) ligand 2 [S	Cxcl2	2.039	0.000	1.686	0.000	1.697	0.000	2.025	0.000
ENSMUSG00000014905	DnaJ (Hsp40) homolog, subfamily B,	Dnajb9	0.672	0.000	1.573	0.000	0.871	0.437	1.214	0.122
ENSMUSG00000020048	heat shock protein 90, beta (Grp94)	Hsp90b1	0.839	0.039	1.580	0.000	1.186	0.129	1.118	0.345
ENSMUSG00000079227	chemokine (C-C motif) receptor 5 [S	Ccr5	0.705	0.000	0.632	0.000	0.568	0.000	0.785	0.000
ENSMUSG00000039646	vasorin [Source:MGI Symbol;Acc:M	Vasn	1.250	0.007	0.657	0.000	1.027	1.000	0.800	0.003
ENSMUSG00000047735	sterile alpha motif domain containin	Samd9l	1.674	0.000	0.638	0.000	1.228	0.088	0.870	0.255
ENSMUSG00000022512	claudin 1 [Source:MGI Symbol;Acc:	Cldn1	0.921	0.489	0.645	0.000	0.920	0.675	0.646	0.000
ENSMUSG00000030357	FK506 binding protein 4 [Source:MG	Fkbp4	0.750	0.000	1.580	0.000	1.228	0.048	0.964	0.936
ENSMUSG00000039218	serine/arginine repetitive matrix 2 [	Srrm2	1.522	0.000	0.654	0.000	1.193	0.180	0.834	0.052
ENSMUSG00000023088	ATP-binding cassette, sub-family C (	Abcc1	1.490	0.000	1.598	0.000	1.620	0.000	1.469	0.000
ENSMUSG00000033705	START domain containing 9 [Source	Stard9	1.672	0.000	0.655	0.000	1.146	0.420	0.955	0.883
ENSMUSG00000019122	chemokine (C-C motif) ligand 9 [Sou	Ccl9	0.565	0.000	1.545	0.000	0.971	0.008	0.899	0.312
ENSMUSG00000044217	aquaporin 5 [Source:MGI Symbol;Ac	Aqp5	0.429	0.000	1.428	0.000	0.840	0.344	0.729	0.002
ENSMUSG00000035545	leukocyte receptor cluster (LRC) me	Leng8	1.696	0.000	0.656	0.000	1.207	0.255	0.921	0.724
ENSMUSG00000070390	NLR family, pyrin domain containing	Nlrp1b	1.570	0.000	0.649	0.000	1.044	0.994	0.976	0.994
ENSMUSG00000028124	glutamate-cysteine ligase, modifier	Gclm	1.634	0.000	1.608	0.000	1.528	0.000	1.720	0.000
ENSMUSG00000025980	heat shock protein 1 (chaperonin) [	Hspd1	0.892	0.228	1.569	0.000	1.157	0.340	1.209	0.021
ENSMUSG00000085814			0.864	0.108	1.563	0.000	1.158	0.232	1.165	0.121
ENSMUSG00000051682	triggering receptor expressed on m	Treml4	1.380	0.000	1.577	0.000	1.606	0.000	1.355	0.000
ENSMUSG00000055897	protein phosphatase 4, regulatory s	Ppp4r1l-ps	1.526	0.000	0.637	0.000	1.054	0.965	0.922	0.709
ENSMUSG00000024401	tumor necrosis factor [Source:MGI	Tnf	1.348	0.000	1.598	0.000	1.418	0.000	1.519	0.000
ENSMUSG00000020571	protein disulfide isomerase associat	Pdia6	0.781	0.002	1.552	0.000	1.062	0.884	1.141	0.242
ENSMUSG00000040663	cardiotrophin-like cytokine factor 1	Clcf1	1.507	0.000	0.628	0.000	0.900	0.352	1.052	0.812
ENSMUSG00000001588	ArfGAP with coiled-coil, ankyrin rep	Acap1	1.102	0.382	0.662	0.000	0.920	0.594	0.793	0.004
ENSMUSG00000021256	vasohibin 1 [Source:MGI Symbol;Ac	Vash1	1.308	0.000	0.659	0.000	0.755	0.000	1.140	0.217
ENSMUSG00000024653	secretoglobin, family 1A, member 1	Scgb1a1	0.805	0.007	1.562	0.000	0.757	0.000	1.660	0.000
ENSMUSG00000052974	cytochrome P450, family 2, subfami	Cyp2f2	0.746	0.001	1.527	0.000	0.806	0.074	1.414	0.000
ENSMUSG00000092686		AC152453.1	0.825	0.008	1.501	0.000	1.269	0.007	0.975	0.981
ENSMUSG00000025934	glutathione S-transferase, alpha 3 [S	Gsta3	1.067	0.579	1.624	0.000	1.242	0.016	1.395	0.000
ENSMUSG00000055116	aryl hydrocarbon receptor nuclear t	Arntl	0.950	0.728	0.675	0.000	0.594	0.000	1.081	0.646
ENSMUSG00000027483	BPI fold containing family A, membe	Bpifa1	0.829	0.049	1.425	0.000	0.645	0.000	1.833	0.000
ENSMUSG00000022136	DnaJ (Hsp40) homolog, subfamily C,	Dnajc3	0.753	0.000	1.521	0.000	0.968	0.963	1.183	0.104
ENSMUSG00000028111	cathepsin K [Source:MGI Symbol;Ac	Ctsk	1.299	0.001	1.535	0.000	1.039	0.963	1.918	0.000

ENSMUSG00000044103	interleukin 1 family, member 9 [Sou	Il1f9	1.320	0.000	1.501	0.000	1.629	0.000	1.215	0.115
ENSMUSG00000000823	zinc finger protein 512B [Source:MG	Znf512b	1.359	0.000	0.665	0.000	1.039	0.989	0.870	0.225
ENSMUSG00000028410	DnaJ (Hsp40) homolog, subfamily A,	Dnaja1	0.841	0.100	1.567	0.000	0.998	1.000	1.321	0.000
ENSMUSG00000021701	polo-like kinase 2 [Source:MGI Sym	Plk2	1.214	0.004	1.562	0.000	1.360	0.000	1.395	0.000
ENSMUSG00000039234	Sec24 related gene family, member	Sec24d	0.995	0.796	1.500	0.000	1.142	0.676	1.307	0.000
ENSMUSG00000003814	calreticulin [Source:MGI Symbol;Acc	Calr	0.819	0.015	1.510	0.000	1.143	0.395	1.083	0.637
ENSMUSG00000003545	FBJ osteosarcoma oncogene B [Sour	Fosb	1.535	0.000	0.681	0.000	0.975	0.925	1.073	0.588
ENSMUSG00000040466	biliverdin reductase B (flavin reduct	Blvrb	1.505	0.000	1.536	0.000	1.381	0.000	1.675	0.000
ENSMUSG00000084956	predicted gene 16194 [Source:MGI	Gm16194	1.547	0.000	0.686	0.000	1.352	0.000	0.785	0.002
ENSMUSG00000037944	chemokine (C-C motif) receptor 7 [S	Ccr7	1.219	0.051	0.654	0.000	0.579	0.000	1.378	0.000
ENSMUSG00000044783	Holliday junction recognition protei	Hjurp	1.755	0.000	0.671	0.000	1.505	0.000	0.783	0.009
ENSMUSG00000029455	aldehyde dehydrogenase 2, mitoch	Aldh2	1.188	0.047	1.510	0.000	1.357	0.000	1.322	0.000
ENSMUSG00000058135	glutathione S-transferase, mu 1 [So	Gstm1	1.294	0.000	1.505	0.000	1.336	0.001	1.458	0.000
ENSMUSG00000015120	ubiquitin-conjugating enzyme E2I [S	Ube2i	1.544	0.000	0.677	0.000	1.222	0.100	0.855	0.164
ENSMUSG00000030218	matrix Gla protein [Source:MGI Sym	Mgp	1.068	0.873	1.517	0.000	1.717	0.000	0.944	1.000
ENSMUSG00000025019	ligand dependent nuclear receptor	Lcor	1.543	0.000	0.692	0.000	1.293	0.001	0.826	0.028
ENSMUSG00000024299	a disintegrin-like and metallopeptid	Adamts10	1.554	0.000	0.681	0.000	1.117	0.762	0.947	0.875
ENSMUSG00000054986	SEC14-like 3 (S. cerevisiae) [Source:	Sec14l3	0.781	0.007	1.576	0.000	1.114	0.613	1.105	0.680
ENSMUSG00000000204	schlafen 4 [Source:MGI Symbol;Acc:	Slfn4	1.551	0.000	0.657	0.000	0.948	0.871	1.075	0.759
ENSMUSG00000050075	G protein-coupled receptor 171 [So	Gpr171	0.761	0.000	0.714	0.000	0.617	0.000	0.881	0.136
ENSMUSG00000000244	tetraspanin 32 [Source:MGI Symbol	Tspan32	1.809	0.000	1.485	0.000	1.652	0.000	1.626	0.000
ENSMUSG00000029379	chemokine (C-X-C motif) ligand 3 [S	Cxcl3	1.576	0.000	1.549	0.000	1.357	0.001	1.798	0.000
ENSMUSG00000047036	zinc finger protein 445 [Source:MGI	Zfp445	1.511	0.000	0.697	0.000	1.223	0.056	0.861	0.159
ENSMUSG00000003352	calcium channel, voltage-dependen	Cacnb3	1.311	0.009	0.640	0.000	0.626	0.000	1.339	0.000
ENSMUSG00000068742	cryptochrome 2 (photolyase-like) [S	Cry2	1.093	0.306	1.456	0.000	1.835	0.000	0.867	0.296
ENSMUSG00000029762	aldo-keto reductase family 1, memb	Akr1b8	1.425	0.000	1.519	0.000	1.191	0.094	1.818	0.000
ENSMUSG00000059937			1.897	0.000	0.693	0.000	1.396	0.000	0.941	0.816
ENSMUSG00000073418	complement component 4B (Chido	C4b	0.601	0.000	0.762	0.000	0.876	0.273	0.523	0.000
ENSMUSG00000043953	chemokine (C-C motif) receptor-like	Ccrl2	1.361	0.000	1.445	0.000	1.548	0.000	1.271	0.001
ENSMUSG00000049866	ADP-ribosylation factor-like 4C [Sou	Arl4c	0.854	0.105	0.703	0.000	0.601	0.000	1.000	0.797
ENSMUSG00000021996	esterase D/formylglutathione hydro	Esd	1.401	0.000	1.478	0.000	1.322	0.001	1.566	0.000
ENSMUSG00000078942	NLR family, apoptosis inhibitory pro	Naip6	1.578	0.000	0.702	0.000	1.188	0.122	0.933	0.701
ENSMUSG00000028691	peroxiredoxin 1 [Source:MGI Symbo	Prdx1	1.364	0.000	1.456	0.000	1.255	0.027	1.582	0.000
ENSMUSG00000037411	serine (or cysteine) peptidase inhibi	Serpine1	0.891	0.155	1.403	0.000	1.503	0.000	0.832	0.064

ENSMUSG00000024074	cysteine rich transmembrane BMP r	Crim1	0.353	0.000	1.308	0.000	1.045	0.943	0.442	0.000
ENSMUSG00000043017	prostaglandin I receptor (IP) [Source	Ptgir	1.531	0.000	1.429	0.000	1.304	0.001	1.678	0.000
ENSMUSG00000029581	fascin homolog 1, actin bundling pro	Fscn1	1.228	0.048	0.684	0.000	0.564	0.000	1.489	0.000
ENSMUSG00000020893	period homolog 1 (Drosophila) [Sou	Per1	1.724	0.000	0.731	0.000	1.538	0.000	0.819	0.013
ENSMUSG00000036875	DNA replication helicase 2 homolog	Dna2	1.521	0.000	0.719	0.000	1.123	0.452	0.975	0.941
ENSMUSG00000038776	epoxide hydrolase 1, microsomal [S	Ephx1	1.210	0.013	1.383	0.000	1.111	0.585	1.506	0.000
ENSMUSG00000031762	metallothionein 2 [Source:MGI Sym	Mt2	1.723	0.000	1.485	0.000	1.288	0.033	1.987	0.000
ENSMUSG00000074604	microsomal glutathione S-transfera	Mgst2	1.451	0.000	1.410	0.000	1.308	0.001	1.565	0.000
ENSMUSG00000024597	solute carrier family 12, member 2 [	Slc12a2	0.619	0.000	1.251	0.000	0.877	0.492	0.884	0.898
ENSMUSG00000045394	epithelial cell adhesion molecule [So	Epcam	0.482	0.000	0.691	0.000	0.718	0.000	0.463	0.000
ENSMUSG00000027533	fatty acid binding protein 5, epiderm	Fabp5	1.402	0.002	0.693	0.000	1.546	0.000	0.629	0.000
ENSMUSG00000064147	RAB44, member RAS oncogene fam	Rab44	1.522	0.000	0.745	0.000	1.143	0.417	0.993	1.000
ENSMUSG00000028341	nuclear receptor subfamily 4, group	Nr4a3	1.203	0.051	0.720	0.000	0.638	0.000	1.358	0.000
ENSMUSG00000003849	NAD(P)H dehydrogenase, quinone 1	Nqo1	1.523	0.000	1.407	0.000	1.398	0.000	1.532	0.000
ENSMUSG00000029380	chemokine (C-X-C motif) ligand 1 [S	Cxcl1	1.279	0.000	1.438	0.000	1.159	0.109	1.586	0.000
ENSMUSG00000078122	RIKEN cDNA F630028O10 gene [Sou€	0028O10Ri	1.572	0.000	0.744	0.000	1.028	1.000	1.138	0.278
ENSMUSG00000021360	glucosaminyl (N-acetyl) transferase	Gcnt2	0.626	0.000	0.748	0.000	0.759	0.000	0.617	0.000
ENSMUSG00000027994	coiled-coil domain containing 109B	Ccdc109b	0.691	0.000	0.719	0.000	0.982	0.906	0.506	0.000
ENSMUSG00000020340	cytoplasmic FMR1 interacting prote	Cyfip2	1.388	0.000	1.352	0.000	1.514	0.000	1.239	0.003
ENSMUSG00000050359	small proline-rich protein 1A [Sourc	Sprr1a	0.540	0.000	0.809	0.000	0.663	0.000	0.659	0.000
ENSMUSG00000031779	chemokine (C-C motif) ligand 22 [So	Ccl22	1.147	0.206	0.731	0.000	0.600	0.000	1.396	0.000
ENSMUSG00000024140	endothelial PAS domain protein 1 [S	Epas1	1.817	0.000	1.326	0.000	1.131	0.225	2.131	0.000
ENSMUSG00000027187	catalase [Source:MGI Symbol;Acc:M	Cat	1.461	0.000	1.333	0.001	1.563	0.000	1.246	0.005
ENSMUSG00000035299	midline 1 [Source:MGI Symbol;Acc:	Mid1	0.512	0.000	0.806	0.001	1.303	0.007	0.317	0.000
ENSMUSG00000020101	RIKEN cDNA 4632428N05 gene [Sou€	05Ri	0.648	0.000	1.306	0.001	1.008	0.985	0.839	0.033
ENSMUSG00000031765	metallothionein 1 [Source:MGI Sym	Mt1	1.558	0.000	1.385	0.001	1.284	0.018	1.680	0.000
ENSMUSG00000026193	fibronectin 1 [Source:MGI Symbol;A	Fn1	0.637	0.000	0.768	0.001	0.653	0.000	0.750	0.000
ENSMUSG00000035783	actin, alpha 2, smooth muscle, aort	Acta2	1.251	0.003	1.359	0.001	1.127	0.489	1.509	0.000
ENSMUSG00000085148	Mir22 host gene (non-protein codin	Mir22hg	1.541	0.000	0.768	0.001	1.053	0.955	1.125	0.287
ENSMUSG00000020044	tissue inhibitor of metalloproteinas	Timp3	0.559	0.000	1.277	0.001	0.877	0.624	0.815	0.046
ENSMUSG00000010651	acetyl-Coenzyme A acyltransferase	Acaa1b	0.782	0.003	0.767	0.001	1.147	0.496	0.524	0.000
ENSMUSG00000020334	solute carrier family 22 (organic cati	Slc22a4	1.511	0.000	1.332	0.001	1.314	0.000	1.533	0.000
ENSMUSG00000044037	ALS2 C-terminal like [Source:MGI Sy	Als2cl	1.693	0.000	0.762	0.001	1.110	0.706	1.162	0.095
ENSMUSG00000038740	family with sequence similarity 125,	Fam125b	0.710	0.000	1.230	0.002	1.366	0.000	0.639	0.000

ENSMUSG00000021579	leucine rich repeat containing 14B [	Lrrc14b	1.574	0.000	1.317	0.002	1.294	0.000	1.602	0.000
ENSMUSG00000021508	chemokine (C-X-C motif) ligand 14 [	Cxcl14	0.783	0.006	0.783	0.002	0.925	0.780	0.662	0.000
ENSMUSG00000028255	chloride channel calcium activated	Clca3	0.652	0.000	1.146	0.002	0.651	0.000	1.147	0.007
ENSMUSG00000021789	surfactant associated protein A1 [So	Sftpa1	0.362	0.000	1.268	0.003	0.831	0.270	0.552	0.000
ENSMUSG00000024053	elastin microfibril interfacer 2 [Sour	Emilin2	0.357	0.000	0.789	0.004	0.869	0.355	0.324	0.000
ENSMUSG00000090307	RIKEN cDNA 1700071M16 gene [So 7		0.649	0.000	1.214	0.005	1.310	0.026	0.601	0.000
ENSMUSG00000030483	cytochrome P450, family 2, subfami	Cyp2b10	0.603	0.000	1.239	0.005	0.947	0.987	0.789	0.009
ENSMUSG00000017754	phospholipid transfer protein [Sour	Pltp	0.579	0.000	0.779	0.006	0.739	0.000	0.611	0.000
ENSMUSG00000024507	hydroxysteroid (17-beta) dehydroge	Hsd17b4	1.139	0.787	1.290	0.006	0.974	0.377	1.508	0.000
ENSMUSG00000026358	regulator of G-protein signaling 1 [S	Rgs1	1.814	0.000	0.805	0.006	1.150	0.031	1.270	0.017
ENSMUSG00000026417	polymeric immunoglobulin receptor	Pigr	0.771	0.011	1.186	0.009	0.622	0.000	1.472	0.000
ENSMUSG00000014846	tubulin polymerization-promoting p	Tppp3	0.653	0.000	0.782	0.009	0.779	0.003	0.655	0.000
ENSMUSG00000020658	EFR3 homolog B (S. cerevisiae) [Sou	Efr3b	1.441	0.000	1.338	0.012	1.271	0.203	1.518	0.000
ENSMUSG00000033590	myosin VC [Source:MGI Symbol;Acc	Myo5c	0.665	0.000	1.176	0.016	0.882	0.681	0.886	0.727
ENSMUSG00000092274	nuclear paraspeckle assembly trans	Neat1	1.559	0.000	0.817	0.017	1.162	0.659	1.096	0.438
ENSMUSG00000031075	anoctamin 1, calcium activated chlo	Ano1	0.660	0.000	1.238	0.017	0.955	0.984	0.855	0.202
ENSMUSG00000029822	oxysterol binding protein-like 3 [Sou	Osbpl3	1.551	0.000	1.243	0.018	1.429	0.000	1.349	0.000
ENSMUSG00000028480	GLI pathogenesis-related 2 [Source:	Glipr2	0.721	0.000	0.809	0.021	0.663	0.000	0.879	0.240
ENSMUSG00000041921	methionyl aminopeptidase type 1D	Metap1d	1.502	0.000	0.810	0.023	1.186	0.212	1.026	0.923
ENSMUSG00000026576	ATPase, Na+/K+ transporting, beta 1	Atp1b1	0.581	0.000	1.214	0.025	0.828	0.232	0.852	0.149
ENSMUSG00000033295	protein tyrosine phosphatase, recep	Ptprf	0.575	0.000	1.212	0.027	0.869	0.639	0.802	0.009
ENSMUSG00000085882	RIKEN cDNA 2610507I01 gene [Sour	610507I01Ri	3.204	0.000	0.793	0.032	1.796	0.000	1.414	0.000
ENSMUSG00000038060	deleted in lung and esophageal canc	Dlec1	0.864	0.110	0.819	0.035	1.116	0.734	0.634	0.000
ENSMUSG00000023959	chloride intracellular channel 5 [Sou	Clic5	0.432	0.000	1.181	0.042	0.853	0.561	0.599	0.000
ENSMUSG00000037095	leucine-rich alpha-2-glycoprotein 1	Lrg1	0.476	0.000	1.116	0.043	0.814	0.125	0.653	0.000
ENSMUSG00000049130	complement component 5a recepto	C5ar1	0.665	0.000	0.823	0.045	0.937	0.806	0.584	0.000
ENSMUSG00000062148	eosinophil-associated, ribonuclease	Ear6	1.201	0.152	1.215	0.046	0.953	0.702	1.530	0.000
ENSMUSG00000032842	ATP-binding cassette, sub-family C (	Abcc10	0.761	0.001	0.849	0.054	0.983	0.989	0.657	0.000
ENSMUSG00000021573	tubulin polymerization promoting p	Tppp	0.562	0.000	1.186	0.055	0.896	0.576	0.744	0.000
ENSMUSG00000087263	predicted gene 15726 [Source:MGI	Gm15726	0.575	0.000	0.870	0.060	1.167	0.447	0.429	0.000
ENSMUSG00000054932	alpha fetoprotein [Source:MGI Sym	Afp	1.468	0.000	1.221	0.067	1.172	0.365	1.529	0.000
ENSMUSG00000030159	C-type lectin domain family 1, mem	Clec1b	1.685	0.000	0.841	0.069	0.831	0.026	1.705	0.000
ENSMUSG00000030739	myosin, heavy polypeptide 14 [Sour	Myh14	0.507	0.000	1.180	0.075	0.865	0.466	0.692	0.000
ENSMUSG00000093077	microRNA 5105 [Source:MGI Symbo	Mir5105	0.628	0.000	0.745	0.075	0.568	0.000	0.824	0.423

ENSMUSG00000018849	WW, C2 and coiled-coil domain con	Wwc1	0.635	0.000	1.193	0.079	0.835	0.374	0.906	0.481
ENSMUSG00000062515	fatty acid binding protein 4, adipocy	Fabp4	1.511	0.000	0.830	0.084	1.674	0.000	0.750	0.001
ENSMUSG00000022097	surfactant associated protein C [Sou	Sftpc	0.297	0.000	1.219	0.087	0.934	0.780	0.387	0.000
ENSMUSG00000050370	cholesterol 25-hydroxylase [Source:	Ch25h	1.321	0.000	1.192	0.088	0.865	0.123	1.822	0.000
ENSMUSG00000032216	neural precursor cell expressed, dev	Nedd4	0.639	0.000	1.139	0.100	0.835	0.303	0.872	0.456
ENSMUSG00000023951	vascular endothelial growth factor A	Vegfa	0.528	0.000	1.179	0.109	0.855	0.396	0.729	0.000
ENSMUSG00000036523	gene regulated by estrogen in breas	Greb1	2.162	0.000	0.835	0.120	1.699	0.000	1.062	0.278
ENSMUSG00000056370	surfactant associated protein B [Sou	Sftpb	0.356	0.000	1.149	0.132	0.831	0.239	0.492	0.000
ENSMUSG00000041247	lysosomal-associated membrane pr	Lamp3	0.592	0.000	1.157	0.138	0.820	0.282	0.835	0.033
ENSMUSG00000022797	transferrin receptor [Source:MGI Sy	Tfrc	1.655	0.000	1.188	0.147	1.290	0.001	1.524	0.000
ENSMUSG00000024462	gamma-aminobutyric acid (GABA) B	Gabbr1	1.019	0.904	0.869	0.158	1.484	0.000	0.596	0.000
ENSMUSG00000036896	complement component 1, q subco	C1qc	0.672	0.000	0.846	0.158	0.663	0.000	0.857	0.148
ENSMUSG00000058624	guanine deaminase [Source:MGI Sy	Gda	0.659	0.000	1.153	0.160	0.979	0.953	0.776	0.001
ENSMUSG00000028195	cysteine rich protein 61 [Source:MG	Cyr61	0.602	0.000	1.153	0.172	0.814	0.167	0.852	0.115
ENSMUSG00000036887	complement component 1, q subco	C1qa	0.684	0.000	0.820	0.174	0.597	0.000	0.939	0.923
ENSMUSG00000024479	mal, T cell differentiation protein 2	Mal2	0.616	0.000	1.148	0.186	0.836	0.386	0.845	0.064
ENSMUSG00000021182	coiled-coil domain containing 88C [S	Ccdc88c	1.087	0.907	1.141	0.195	1.507	0.000	0.823	0.061
ENSMUSG00000029570	LFNG O-fucosylpeptide 3-beta-N-ac	Lfng	0.638	0.000	0.859	0.198	0.747	0.000	0.733	0.000
ENSMUSG00000049608	G protein-coupled receptor 55 [Sou	Gpr55	1.529	0.000	1.156	0.217	1.587	0.000	1.114	0.061
ENSMUSG00000062006	ribosomal protein L34 [Source:MGI	Rpl34	0.637	0.005	0.816	0.226	1.398	0.000	0.372	0.000
ENSMUSG00000022037	clusterin [Source:MGI Symbol;Acc:M	Clu	0.387	0.000	1.023	0.236	0.673	0.000	0.589	0.000
ENSMUSG00000014686	carcinoembryonic antigen-related c	Ceacam16	1.579	0.000	0.888	0.240	1.341	0.000	1.046	0.854
ENSMUSG00000059463	sperm associated antigen 11B [Sour	Spag11b	1.625	0.000	1.145	0.249	1.191	0.071	1.562	0.000
ENSMUSG00000016206	histocompatibility 2, M region locus	H2-M3	1.750	0.000	0.874	0.271	0.951	0.900	1.609	0.000
ENSMUSG00000059606	eosinophil-associated, ribonuclease	Ear5	1.518	0.000	1.149	0.307	1.136	0.511	1.535	0.000
ENSMUSG00000027070	low density lipoprotein receptor-rel	Lrp2	0.648	0.000	1.105	0.326	0.838	0.351	0.854	0.355
ENSMUSG00000092267	predicted gene 20417 [Source:MGI	Gm20417	1.511	0.000	0.898	0.328	1.831	0.000	0.741	0.000
ENSMUSG00000079491	histocompatibility 2, T region locus	H2-T10	0.462	0.000	0.906	0.334	0.931	0.853	0.449	0.000
ENSMUSG00000025355	matrix metallopeptidase 19 [Source	Mmp19	0.567	0.000	1.117	0.352	0.914	0.365	0.693	0.000
ENSMUSG00000085299			1.927	0.000	0.866	0.355	1.054	0.963	1.584	0.000
ENSMUSG00000020592	syndecan 1 [Source:MGI Symbol;Ac	Sdc1	0.634	0.000	1.108	0.362	0.883	0.736	0.796	0.006
ENSMUSG00000032420	5' nucleotidase, ecto [Source:MGI S	Nt5e	0.630	0.000	0.911	0.384	0.853	0.052	0.673	0.000
ENSMUSG00000029188	solute carrier family 34 (sodium pho	Slc34a2	0.657	0.000	1.113	0.396	0.876	0.800	0.835	0.039
ENSMUSG00000090231	complement factor B [Source:MGI S	Cfb	1.536	0.000	1.113	0.405	1.235	0.060	1.384	0.000

ENSMUSG00000041548	heat shock protein 8 [Source:MGI S	Hspb8	0.551	0.000	1.090	0.412	0.808	0.102	0.745	0.000
ENSMUSG00000029192	TBC1 domain family, member 14 [S	Tbc1d14	0.588	0.000	1.097	0.415	0.787	0.000	0.820	0.007
ENSMUSG00000042349	inhibitor of kappaB kinase epsilon [S	Ikbke	1.424	0.000	1.146	0.420	1.561	0.000	1.045	0.846
ENSMUSG00000092341	metastasis associated lung adenoca	Malat1	1.575	0.000	0.916	0.424	2.180	0.000	0.662	0.000
ENSMUSG00000092511	predicted gene 20547 [Source:MGI	Gm20547	1.543	0.000	1.106	0.447	1.226	0.078	1.392	0.000
ENSMUSG00000022661	CD200 antigen [Source:MGI Symbol	Cd200	1.637	0.000	1.102	0.452	1.206	0.087	1.496	0.000
ENSMUSG00000067212	histocompatibility 2, T region locus	H2-T23	1.952	0.000	1.103	0.462	0.984	0.979	2.187	0.000
ENSMUSG00000015478	ring finger protein 5 [Source:MGI Sy	Rnf5	0.603	0.000	1.077	0.467	1.007	1.000	0.645	0.000
ENSMUSG00000039953	calsyntenin 1 [Source:MGI Symbol;A	Clstn1	1.277	0.000	1.121	0.494	0.937	0.762	1.527	0.000
ENSMUSG00000026380	transcription factor CP2-like 1 [Sour	Tfcp2l1	0.659	0.000	1.073	0.513	0.942	0.943	0.751	0.001
ENSMUSG00000023043	keratin 18 [Source:MGI Symbol;Acc:	Krt18	0.555	0.000	1.064	0.517	0.815	0.250	0.725	0.000
ENSMUSG00000031089	solute carrier family 6 (neurotransm	Slc6a14	0.627	0.000	1.076	0.540	0.838	0.394	0.804	0.014
ENSMUSG00000059325	HOP homeobox [Source:MGI Symbo	Hopx	0.633	0.000	1.086	0.544	0.937	0.889	0.734	0.000
ENSMUSG00000018340	annexin A6 [Source:MGI Symbol;Ac	Anxa6	0.501	0.000	1.076	0.565	0.769	0.017	0.702	0.000
ENSMUSG00000029552	testis derived transcript [Source:MG	Tes	0.659	0.000	1.077	0.566	0.695	0.000	1.021	0.983
ENSMUSG00000059089	Fc receptor, IgG, low affinity IV [Sou	Fcgr4	0.596	0.000	1.076	0.585	0.835	0.093	0.768	0.000
ENSMUSG00000053113	suppressor of cytokine signaling 3 [S	Socs3	0.644	0.000	0.940	0.585	0.709	0.000	0.854	0.048
ENSMUSG00000031502	collagen, type IV, alpha 1 [Source:M	Col4a1	0.566	0.000	1.064	0.585	0.884	0.720	0.682	0.000
ENSMUSG00000086695	predicted gene 15247 [Source:MGI	Gm15247	0.738	0.001	0.933	0.585	1.117	0.772	0.616	0.000
ENSMUSG00000016283	histocompatibility 2, M region locus	H2-M2	1.742	0.000	0.915	0.587	0.995	1.000	1.602	0.000
ENSMUSG00000049382	keratin 8 [Source:MGI Symbol;Acc:	Krt8	0.622	0.000	1.074	0.592	0.868	0.609	0.768	0.003
ENSMUSG00000070473	claudin 3 [Source:MGI Symbol;Acc:	Cldn3	0.598	0.000	1.089	0.594	0.854	0.429	0.762	0.002
ENSMUSG00000091549	predicted gene 6548 [Source:MGI S	Gm6548	1.711	0.000	0.906	0.601	0.986	0.987	1.572	0.000
ENSMUSG00000046318	collagen and calcium binding EGF do	Ccbe1	1.669	0.000	0.946	0.613	1.202	0.082	1.314	0.009
ENSMUSG00000035202	leucyl-tRNA synthetase, mitochondr	Lars2	0.567	0.000	1.039	0.633	0.685	0.000	0.860	0.132
ENSMUSG00000026479	laminin, gamma 2 [Source:MGI Sym	Lamc2	0.474	0.000	0.956	0.646	0.799	0.084	0.567	0.000
ENSMUSG00000030516	tight junction protein 1 [Source:MG	Tjp1	0.653	0.000	1.066	0.654	0.852	0.438	0.818	0.026
ENSMUSG00000006445	Eph receptor A2 [Source:MGI Symb	Epha2	0.550	0.000	0.935	0.657	0.779	0.035	0.661	0.000
ENSMUSG00000047821	tripartite motif-containing 16 [Sourc	Trim16	1.552	0.000	0.941	0.674	1.078	0.826	1.355	0.000
ENSMUSG00000089672	glycoprotein 49 A [Source:MGI Sym	Gp49a	1.544	0.000	0.950	0.675	1.120	0.590	1.308	0.000
ENSMUSG00000024026	glyoxalase 1 [Source:MGI Symbol;A	Glo1	0.524	0.000	1.060	0.690	0.975	0.991	0.570	0.000
ENSMUSG00000032911	chondroitin sulfate proteoglycan 4 [	Cspg4	0.367	0.000	0.924	0.694	0.815	0.140	0.416	0.000
ENSMUSG00000028173	wntless homolog (Drosophila) [Sour	Wls	0.648	0.000	0.940	0.696	0.846	0.042	0.720	0.000
ENSMUSG00000031503	collagen, type IV, alpha 2 [Source:M	Col4a2	0.641	0.000	0.956	0.712	0.864	0.391	0.709	0.000

ENSMUSG00000023036	protocadherin gamma subfamily A,	Pcdhga3	0.648	0.000	0.940	0.713	0.871	0.247	0.699	0.000
ENSMUSG00000092395	predicted gene 20463 [Source:MGI	Gm20463	0.638	0.000	1.030	0.713	0.928	0.898	0.708	0.000
ENSMUSG00000032473	claudin 18 [Source:MGI Symbol;Acc	Cldn18	0.412	0.000	1.074	0.722	0.822	0.225	0.538	0.000
ENSMUSG00000039457	periplakin [Source:MGI Symbol;Acc:	Ppl	0.665	0.000	1.045	0.727	0.889	0.694	0.781	0.003
ENSMUSG00000025504	EPS8-like 2 [Source:MGI Symbol;Acc	Eps8l2	0.656	0.000	1.056	0.737	0.854	0.483	0.812	0.022
ENSMUSG00000040152	thrombospondin 1 [Source:MGI Sym	Thbs1	0.573	0.000	1.088	0.742	0.762	0.009	0.819	0.003
ENSMUSG00000054951	RIKEN cDNA 9130008F23 gene [Sou	130008F23Ri	1.592	0.000	0.932	0.756	0.975	1.000	1.522	0.000
ENSMUSG00000006360	cysteine-rich protein 1 (intestinal) [S	Crip1	0.595	0.000	1.044	0.768	0.589	0.000	1.054	0.971
ENSMUSG00000023039	keratin 7 [Source:MGI Symbol;Acc:	Krt7	0.506	0.000	1.055	0.773	0.777	0.068	0.687	0.000
ENSMUSG00000032076	cell adhesion molecule 1 [Source:M	Cadm1	0.657	0.000	1.054	0.774	0.791	0.078	0.876	0.123
ENSMUSG00000092283	predicted gene 20412 [Source:MGI	Gm20412	0.655	0.000	1.041	0.799	0.900	0.364	0.758	0.001
ENSMUSG00000086324	predicted gene 15564 [Source:MGI	Gm15564	0.578	0.000	1.021	0.821	0.676	0.000	0.873	0.125
ENSMUSG00000024059	CAP-GLY domain containing linker p	Clip4	0.615	0.000	1.040	0.822	0.878	0.606	0.728	0.000
ENSMUSG00000028024	glutamyl aminopeptidase [Source:M	Enpep	0.600	0.000	1.033	0.829	0.845	0.442	0.734	0.000
ENSMUSG00000027030	serine/threonine kinase 39 [Source:	Stk39	0.757	0.000	0.955	0.830	1.090	0.676	0.663	0.000
ENSMUSG00000023979	guanylate cyclase activator 1B [Sour	Guca1b	1.452	0.000	1.048	0.842	0.975	1.000	1.560	0.000
ENSMUSG00000050953	gap junction protein, alpha 1 [Sourc	Gja1	0.569	0.000	0.966	0.855	0.807	0.119	0.681	0.000
ENSMUSG00000015647	laminin, alpha 5 [Source:MGI Symbo	Lama5	0.628	0.000	0.966	0.858	0.871	0.449	0.697	0.000
ENSMUSG00000024486	heparin-binding EGF-like growth fac	Hbegf	0.631	0.000	0.963	0.863	0.913	0.836	0.666	0.000
ENSMUSG00000018593	secreted acidic cysteine rich glycopr	Sparc	0.535	0.000	1.001	0.864	1.003	0.993	0.534	0.000
ENSMUSG00000057530	endothelin converting enzyme 1 [So	Ece1	0.517	0.000	0.975	0.874	0.715	0.000	0.705	0.000
ENSMUSG00000022505	epithelial membrane protein 2 [Sou	Emp2	0.455	0.000	1.037	0.875	0.865	0.542	0.546	0.000
ENSMUSG00000022899	solute carrier family 15 (H+/peptide	Slc15a2	1.511	0.000	0.978	0.883	1.321	0.001	1.119	0.399
ENSMUSG00000092254	RIKEN cDNA A930015D03 gene [Sou	930015D03Ri	0.645	0.000	1.027	0.896	0.996	1.000	0.666	0.000
ENSMUSG00000036905	complement component 1, q subco	C1qb	0.636	0.000	0.982	0.896	0.675	0.000	0.926	0.904
ENSMUSG00000060969	Iroquois related homeobox 1 (Droso	Irx1	0.652	0.000	1.034	0.905	0.867	0.736	0.777	0.002
ENSMUSG00000001473	tubulin, beta 6 class V [Source:MGI	Tubb6	0.659	0.000	1.023	0.907	0.870	0.257	0.775	0.001
ENSMUSG00000076258		AC087541.1	0.622	0.000	0.952	0.915	0.658	0.000	0.900	0.117
ENSMUSG00000035441	myosin ID [Source:MGI Symbol;Acc:	Myo1d	0.666	0.000	1.006	0.926	0.876	0.523	0.765	0.000
ENSMUSG00000032280	transducin-like enhancer of split 3,	Tle3	0.683	0.000	1.008	0.926	1.039	0.924	0.662	0.000
ENSMUSG00000021795	surfactant associated protein D [Sou	Sftpd	0.489	0.000	1.033	0.926	0.836	0.300	0.604	0.000
ENSMUSG00000046711	high mobility group AT-hook 1 [Sour	Hmga1	2.151	0.000	1.017	0.928	1.141	0.238	1.916	0.000
ENSMUSG00000026604	protein tyrosine phosphatase, non-r	Ptpn14	0.667	0.000	1.021	0.928	0.933	0.921	0.729	0.000
ENSMUSG00000072812	AHNAK nucleoprotein 2 [Source:MG	Ahnak2	1.456	0.000	1.039	0.932	0.957	0.829	1.581	0.000

ENSMUSG00000029375	chemokine (C-X-C motif) ligand 15 [	Cxcl15	0.597	0.000	0.994	0.934	0.821	0.139	0.722	0.000
ENSMUSG00000089637			0.418	0.000	1.030	0.937	0.877	0.716	0.490	0.000
ENSMUSG00000078249	high mobility group AT-hook I, relat	Hmga1-rs1	0.418	0.000	1.030	0.937	0.877	0.716	0.490	0.000
ENSMUSG00000051177	phospholipase C, beta 1 [Source:MG	Plcb1	0.646	0.000	0.980	0.939	1.009	1.000	0.627	0.000
ENSMUSG00000076441	argininosuccinate synthetase 1 [Sou	Ass1	1.389	0.000	0.999	0.950	0.847	0.336	1.638	0.000
ENSMUSG00000075391	predicted gene 13443 [Source:MGI	Gm13443	0.642	0.000	0.987	0.952	0.928	0.889	0.683	0.000
ENSMUSG00000033149	pleckstrin homology-like domain, fa	Phldb2	0.661	0.000	0.987	0.959	0.893	0.726	0.732	0.000
ENSMUSG00000028128	coagulation factor III [Source:MGI S	F3	0.629	0.000	1.021	0.961	0.798	0.080	0.804	0.009
ENSMUSG00000037820	transglutaminase 2, C polypeptide [	Tgm2	0.660	0.000	0.987	0.966	0.906	0.451	0.720	0.000
ENSMUSG00000056492	G protein-coupled receptor 116 [So	Gpr116	0.587	0.000	1.017	0.968	0.849	0.554	0.703	0.000
ENSMUSG00000063458	RIKEN cDNA 1700112E06 gene [Sou	700112E06Ri	0.529	0.000	0.979	0.970	0.776	0.088	0.668	0.000
ENSMUSG00000092984	microRNA 5115 [Source:MGI Symbo	Mir5115	0.656	0.000	0.986	0.990	0.661	0.000	0.979	0.938
ENSMUSG00000028327	RIKEN cDNA 1300002K09 gene [Sou	300002K09Ri	1.838	0.000	1.005	0.994	1.136	0.432	1.625	0.000
ENSMUSG00000029378	amphiregulin [Source:MGI Symbol;A	Areg	0.581	0.000	1.022	1.000	0.870	0.393	0.683	0.000
ENSMUSG00000059040	predicted gene 5506 [Source:MGI S	Gm5506	1.874	0.000	1.040	1.000	0.973	1.000	2.002	0.000
ENSMUSG00000046733	G protein-coupled receptor, family	Gprc5a	0.448	0.000	1.007	1.000	0.803	0.152	0.563	0.000
ENSMUSG00000047090	transmembrane protein 198b [Sour	Tmem198b	0.645	0.000	0.996	1.000	0.852	0.081	0.754	0.000
ENSMUSG00000049001	neuron-derived neurotrophic factor	Ndnf	0.442	0.000	1.000	1.000	0.772	0.075	0.572	0.000
ENSMUSG00000032366	tropomyosin 1, alpha [Source:MGI S	Tpm1	0.643	0.000	1.010	1.000	0.862	0.351	0.754	0.000
ENSMUSG00000033060	LIM domain only 7 [Source:MGI Sym	Lmo7	0.417	0.000	1.009	1.000	0.816	0.171	0.516	0.000

Supplementary Table 2. Upstream regulators predicted to be activated in AM from RAGE null mice compared to wild-type (WT) mice.

Upstream Regulator	Type	RAGE-CT / WT-CT		RAGE-CS / WT-CS	
		z-score	p-val	z-score	p-val
Beta-estradiol	Chemical	2.641	3.78E-20	0.717	1.06E-11
Transforming growth factor beta 1	Growth factor	3.312	3.85E-15	-0.056	3.40E-11
Endothelin 1	Cytokine	3.040	4.88E-12	ns	ns
Protein phosphatase 3 regulatory subunit B, alpha	Phosphatase	2.580	7.29E-10	1.970	8.05E-06
Nitric oxide	Chemical	-2.613	5.80E-08	-2.577	1.51E-07
Zinc finger E-box binding homeobox 1	TF	-2.433	9.27E-07	-2.00	6.66E-05
Cytochrome P450 1a1	Enzyme	-2.400	1.30E-06	-2.183	1.77E-04
Connective tissue growth factor	Growth factor	2.623	1.68E-05	ns	1.62E-02

Both control (CT) and cigarette smoke (CS) exposed genotype comparisons are shown. Table denotes description and type (transcription factor (TF), growth factor, cytokine etc.) of upstream regulator together with Fisher exact overlap p-value and activation Z-score as output from Ingenuity Pathway Analysis (IPA). The overlap p-value calls likely upstream regulators based on significant overlap between differentially expressed genes and known gene targets. The activation Z-score infers activation or inhibition of upstream regulators based on the direction of fold change of differentially expressed target genes. Comparison values with z-score statistics > 2 or < -2 are highlighted.

Supplementary Table 3. Predicted Upstream Regulators

				RAGE null vs C57 WT control	
				7-Day[1]	4-Month
Growth factors	*Symbol*	RefSeq ID	Prediction	Fold	Fold
Transforming growth factor beta 1 (TGFB1)					
Chondroitin sulfate proteoglycan 4	*Cspg4*	NM_139001	Activated	**2.7**	**2.8**
Clusterin	*Clu*	NM_013492	Activated	**2.6**	**2.2**
Lammin, gamma 2	*Lamc2*	NM_008485	Activated	**2.1**	**1.8**
Keratin 7	*Krt7*	NM_033073	Activated	**2.0**	**1.5**
Vascular endothelial growth factor A	*Vegfa*	NM_001025250*	Activated	**1.9**	**1.5**
Secreted acidic cysteine rich glycoprotein	*Sparc*	NM_009242*	Activated	**1.9**	**1.6**
Keratin 18	*Krt18*	NM_010664	Activated	**1.8**	**1.6**
Tissue inhibitor of metalloproteinase 3	*Timp3*	NM_011595	Activated	**1.8**	**1.6**
Collage, type IV, alpha 1	*Col4a1*	NM_009931	Activated	**1.8**	1.2
Gap junction protein, alpha 1	*Gja1*	NM_010288	Activated	**1.8**	**1.5**
Thrombospondin 1	*Thbs1*	NM_011580*	Activated	**1.7**	**1.9**
Amphiregulin	*Areg*	NM_009704	Activated	**1.7**	ns
Fc receptor, IgG, low affinity IV	*Fcgr4*	NM_144559	Activated	**1.7**	1.4
Cysteine rich protein 61	*Cyr61*	NM_010516	Activated	**1.7**	1.4
Coagulation factor III	*F3*	NM_010171	Activated	**1.6**	**1.5**
5' nucleotidase, ecto	*Nt5e*	NM_011851	Activated	**1.6**	**1.9**
Heparin-binding EGF-like growth factor	*Hbegf*	NM_010415	Activated	**1.6**	1.4
Syndecan 1	*Sdc1*	NM_011519	Activated	**1.6**	1.4
Complement component 1, q subcomponent, beta polyp	*C1qb*	NM_009777	Activated	**1.6**	ns
Fibronectin 1	*Fn1*	NM_010233*	Activated	**1.6**	**1.8**
Collage, type IV, alpha 2	*Col4a2*	NM_009932	Activated	**1.6**	ns
Tropomyosin 1	*Tpm1*	NM_024427*	Activated	**1.6**	ns
Suppressor of cytokine signaling 3	*Socs3*	NM_007707	Activated	**1.6**	**2.2**
Heat shock protein 5	*Hspa5*	NM_001163434*	Activated	**1.6**	**1.5**
Transglutaminase 2, C polypeptide	*Tgm2*	NM_009373	Activated	**1.5**	ns
Complement component 5a receptor 1	*C5ar1*	NM_007577*	Activated	**1.5**	**1.5**
Heat shock protein 1A	*Hspa1a*	NM_010479	Activated	**-1.9**	-1.4
Chemokine (C-X-C motif) ligand 3	*Cxcl3*	NM_203320	Activated	**-1.6**	-1.4
Aryl-hydrocarbon receptor	*Ahr*	NM_013464*	Activated	ns	**1.7**
Chemokine (C-C motif) receptor 1	*Ccr1*	NM_009912	Activated	1.4	**1.8**

	CD14 antigen	*Cd14*	NM_009841	Activated	1.2	**4.0**
	Carboxylesterase 2C	*Ces2c*	NM_145603	Activated	-1.2	**-1.7**
	Collagen, type VI, alpha 3	*Col6a3*	NM_001243008*	Activated	ns	**1.6**
	Disabled 2	*Dab2*	NM_023118*	Activated	1.4	**1.9**
	Death associated protein kinase 1	*Dapk1*	NM_029653*	Activated	ns	**1.6**
	DNA methyltransferase (cytosine-5) 1	*Dnmt1*	NM_001199431*	Activated	-1.2	**-1.6**
	Elastin microfibril interfacer 1	*Emilin1*	NM_133918	Activated	1.4	**2.0**
	Epithelial cell adhesion molecule	*Epcam*	NM_008532	Activated	**2.1**	**1.8**
	FBJ osteosarcoma oncogene	*Fos*	NM_010234	Activated	ns	**1.7**
	Insulin-like growth factor 1	*Igf1*	NM_010512*	Activated	-1.4	**-2.5**
	Interleukin 4 receptor, alpha	*Il4ra*	NM_001008700	Activated	1.4	**1.8**
	Interleukin 6 receptor, alpha	*Il6ra*	NM_010559	Activated	1.4	**1.6**
	Jun B proto-oncogene	*Junb*	NM_008416	Activated	ns	**1.5**
	Killer cell lectin-like receptor subfamily B member 1	*Klrb1*	NM_001099918*	Activated	-1.4	**-2.2**
	Lipoprotein lipase	*Lpl*	NM_008509	Activated	ns	**-1.9**
	Minichromosome maintenance deficient 2 mitotin	*Mcm2*	NM_008564	Activated	ns	**-1.6**
	Purinergic receptor P2Y, G-protein coupled,14	*P2ry14*	NM_133200*	Activated	1.3	**1.6**
	Plasminogen activator, urokinase receptor	*Plaur*	NM_011113	Activated	ns	**1.6**
	Runt related transcription factor 1	*Runx1*	NM_001111021*	Activated	ns	**1.6**
	Vitamin D receptor	*Vdr*	NM_009504	Activated	1.3	**2.4**
	X-box binding protein 1	*Xbp1*	NM_013842*	Activated	1.2	**1.7**
Endothelin 1 (EDN1)						
	Vascular endothelial growth factor A	*Vegfa*	NM_001025250*	Activated	**1.9**	**1.5**
	Tissue inhibitor of metalloproteinase 3	*Timp3*	NM_011595	Activated	**1.8**	**1.6**
	Thrombospondin 1	*Thbs1*	NM_011580*	Activated	**1.7**	**1.9**
	Transglutaminase 2, C polypeptide	*Tgm2*	NM_009373	Activated	**1.5**	ns
	Suppressor of cytokine signaling 3	*Socs3*	NM_007707	Activated	**1.6**	**2.2**
	Syndecan 1	*Sdc1*	NM_011519	Activated	**1.6**	1.4
	Gap junction protein, alpha 1	*Gja1*	NM_010288	Activated	**1.8**	**1.5**
	Fibronectin 1	*Fn1*	NM_010233*	Activated	**1.6**	**1.8**
	Collage, type IV, alpha 1	*Col4a1*	NM_009931	Activated	**1.8**	1.2
	Plasminogen activator, urokinase receptor	*Plaur*	NM_011113	Activated	ns	**1.6**
	Leptin receptor	*Lepr*	NM_146146*	Activated	**1.5**	**2.1**

Jun B proto-oncogene	*Junb*	NM_008416	Activated	ns	**1.5**
FBJ osteosarcoma oncogene	*Fos*	NM_010234	Activated	ns	**1.7**
<u>Connective tissue growth factor (CTGF)</u>					
Vascular endothelial growth factor A	*Vegfa*	NM_001025250*	Activated	**1.9**	**1.5**
Tissue inhibitor of metalloproteinase 3	*Timp3*	NM_011595	Activated	**1.8**	**1.6**
Tropomyosin 1	*Tpm1*	NM_024427*	Activated	**1.6**	ns
Secreted acidic cysteine rich glycoprotein	*Sparc*	NM_009242*	Activated	**1.9**	**1.6**
Fibronectin 1	*Fn1*	NM_010233*	Activated	**1.6**	**1.8**
Collage, type IV, alpha 2	*Col4a2*	NM_009932	Activated	**1.6**	ns
Insulin-like growth factor 1	*Igf1*	NM_010512*	Activated	-1.4	**-2.5**
Elastin microfibril interfacer 1	*Emilin1*	NM_133918	Activated	1.4	**2.0**

1 - NOTES

Red font = statistically significant (Fold ≥ 1.5 and FDR < 0.05)

ns = not significant (FDR > 0.05)

* Gene has more than one isoform

Supplementary Table 4. Predicted Upstream Regulators

Transcription factors	*Symbol*	RefSeq ID	Prediction	7-Day Fold[1] C57	7-Day Fold[1] RAGE	7-Day Fold[1] CS	4-Month Fold C57	4-Month Fold RAGE	4-Month Fold CS
Nuclear factor, erythroid derived 2, like 2 (NFE2L2, NRF2)									
Solute carrier family 7, member 11	*Slc7a11*	NM_011990	Activated	**2.6**	**2.8**	**2.3**	**2.1**	-1.2	**6.2**
Cytochrome P450, family 2, subfamily a, polypeptide 5	*Cyp2a5*	NM_007812	Activated	**2.1**	1.3	1.4	ns	-1.2	ns
Carbonyl reductase 2	*Cbr2*	NM_007621	Activated	**2**	1.2	1.2	ns	-1.4	ns
Heme oxygenase 1	*Hmox1*	NM_010442	Activated	**2**	**1.6**	**2.6**	-1.3	-2.3	**3.9**
Sequestosome 1	*Sqstm1*	NM_011018*	Activated	**2**	ns	**1.6**	ns	ns	**1.6**
Heat shock protein 90, alpha, class A member 1	*Hsp90aa1*	NM_010480	Activated	**1.9**	1.3	1.3	ns	-1.2	ns
Sulfiredoxin 1 homolog	*Srxn1*	NM_029688	Activated	**1.8**	**1.6**	**1.9**	ns	ns	**2.4**
Cysteine and histidine-rich domain-containing, zinc binding protein 1	*Chordc1*	NM_025844	Activated	**1.7**	ns	1.3	ns	-1.6	ns
Heat shock protein 90, alpha, class B member 1	*Hsp90ab1*	NM_008302	Activated	**1.7**	1.4	ns	1.3	ns	ns
Stress-induced phosphoprotein 1	*Stip1*	NM_016737	Activated	**1.7**	ns	ns	ns	-1.3	ns
Thioredoxin reductase 1	*Txnrd1*	NM_001042523*	Activated	**1.7**	**1.5**	**1.6**	1.2	ns	**1.9**
Aldehyde dehydrogenase family 1, subfamily A1	*Aldh1a1*	NM_013467	Activated	**1.7**	ns	1.4	ns	-1.2	ns
ATP-binding cassette, subfamily C, member 1	*Abcc1*	NM_008576	Activated	**1.6**	**1.6**	**1.5**	1.2	ns	**2.2**
Glutamate-cysteine ligase, modifier subunit	*Gclm*	NM_008129	Activated	**1.6**	**1.5**	**1.7**	ns	-1.3	**2.2**
Glutathione S-transferase, alpha 3	*Gsta3*	NM_001077353*	Activated	**1.6**	1.2	1.4	1.3	-1.3	**1.8**
Heat shock protein 90, beta, member 1	*Hsp90b1*	NM_011631	Activated	**1.6**	ns	ns	1.2	ns	ns
Protein disulfide isomerase associated 4	*Pdia4*	NM_009787	Activated	**1.6**	ns	ns	ns	-1.3	ns
Protein disulfide isomerase associated 6	*Pdia6*	NM_027959	Activated	**1.6**	ns	ns	ns	-1.2	ns
Aldo-keto reductase family 1, member B8	*Akr1b8*	NM_008012	Activated	**1.5**	ns	**1.8**	**1.6**	1.4	**2**
DnaJ (Hsp40) homolog, subfamily C, member 3	*Dnajc3*	NM_008929	Activated	**1.5**	ns	ns	ns	-1.3	ns
Esterase D/formylglutathione hydrolase	*Esd*	NM_001285423*	Activated	**1.5**	1.3	**1.6**	1.2	ns	**1.9**
Glutathione S-transferase, mu 1	*Gstm1*	NM_010358	Activated	**1.5**	1.3	**1.5**	**2**	**2.2**	1.3
Peroxiredoxin 1	*Prdx1*	NM_011034	Activated	**1.5**	1.3	**1.6**	ns	ns	**1.9**
Carboxylesterase 1D	*Ces1D*	NM_053200	Activated	**1.5**	ns	ns	ns	ns	ns
Epoxide hydrolase 1	*Ephx1*	NM_010145	Activated	1.4	ns	**1.5**	**1.8**	1.4	**2.4**
Metallothionein 1	*Mt1*	NM_013602	Activated	1.4	1.3	**1.7**	ns	1.3	1.4
NAD(P)H dehydrogenase, quinone 1	*Nqo1*	NM_008706	Activated	1.4	1.4	**1.5**	ns	ns	**2.1**
Endothelial PAS domain protein 1	*Epas1*	NM_010137	Activated	1.3	ns	**2.1**	**2.7**	**1.8**	**5.9**
Fatty acid binding protein 1	*Fabp1*	NM_0179399	Activated	**-1.7**	ns	-1.3	**-2**	**-1.8**	-1.2
Aminolevulinic acid synthase 2, erythroid	*Alas2*	NM_009653*	Activated	**-2**	**-1.5**	ns	1.2	**-1.6**	1.2
Gamma-aminobutyric acid (GABA) B receptor 1	*Gabbr1*	NM_019439	Affected	ns	1.5	**-1.7**	1.2	1.3	**-1.6**
Chemokine (C-X-C motif) ligand 2	*Cxcl2*	NM_009140	Inhibited	**1.7**	**1.7**	**2**	-1.3	-2.1	**2.2**
Tumor necrosis factor	*Tnf*	NM_013693*	Inhibited	**1.6**	1.4	**1.5**	1.3	-1.3	**2.1**
Cathepsin K	*Ctsk*	NM_007802	Inhibited	**1.5**	ns	**1.9**	**1.6**	**1.5**	**1.6**
Chemokine (C-X-C motif) ligand 1	*Cxcl1*	NM_008176	Inhibited	1.4	ns	**1.6**	ns	**-1.5**	1.2
Acid phosphatase 5, tartrate resistant	*Acp5*	NM_001102405*	Inhibited	**1.5**	1.3	1.2	**2**	**1.7**	ns
Cathepsin D	*Ctsd*	NM_009983	Activated	1.4	ns	ns	**1.6**	**1.6**	ns
Malic enzyme 1, NADP(+)-dependent, cytosolic	*Me1*	NM_008615	Activated	1.4	1.2	1.4	**1.6**	ns	**2**

Catalase	*Cat*	NM_009804	Activated	1.3	**1.6**	1.2	**1.6**	ns	**1.8**
Chemokine (C-C motif) ligand 5	*Ccl5*	NM_013653	Activated	ns	-1.4	ns	**-1.6**	-1.3	ns
Profilin 2	*Pfn2*	NM_019410	Activated	1.4	1.3	1.4	ns	-1.3	**1.6**
Oxidative stress induced growth inhibitor 1	*Osgin1*	NM_027950	Activated	1.4	1.2	1.4	ns	1.2	**1.6**
Interleukin 1 alpha	*Il1a*	NM_010554	Activated	ns	ns	1.2	1.2	ns	**1.6**
HIV-1 tat interactive protein 2	*Htatip2*	NM_016865*	Activated	1.2	ns	1.4	1.2	ns	**1.5**
Aminolevulinic acid synthase 1	*Alas1*	NM_001291835*	Activated	1.4	1.3	ns	1.3	-1.4	**3.1**
ATP-binding cassette, subfamily C, member 3	*Abcc3*	NM_029600	Activated	ns	ns	ns	1.3	ns	**1.6**
ATP-binding cassette, subfamily G, member 2	*Abcg2*	NM_011920	Affected	ns	ns	ns	**1.6**	**1.5**	1.2
Superoxide dismutase 2	*Sod2*	NM_013671	Affected	1.4	1.3	1.3	1.2	-1.2	**1.7**
Vascular cell adhesion molecule 1	*Vcam1*	NM_011693	Inhibited	ns	1.1	ns	**1.5**	**2.6**	**-1.5**
Fibronectin 1	*Fn1*	NM_010233*	Inhibited	-1.3	**-1.5**	-1.3	**-2**	**-2.2**	**-1.6**
UDP glucuronosyltransferase 1 family, polypeptide A1	*Ugt1a1*	NM_201645	Inhibited	1.2	ns	ns	ns	1.3	**-1.8**
Solute carrier family 30, member 4	*Slc30a4*	NM_011774*	Inhibited	ns	ns	-1.2	ns	1.5	**-1.6**
Phorbol-12-myristate-13-acetate-induced protein 1	*Pmaip1*	NM_021451	Inhibited	1.2	ns	1.4	ns	ns	**1.9**
Myocyte enhancer factor 2C	*Mef2c*	NM_001170537*	Inhibited	ns	ns	ns	-1.2	1.3	**-1.6**
Lipoprotein lipase	*Lpl*	NM_008509	Inhibited	-1.3	-1.4	1.2	-1.5	-1.4	**1.8**
Aryl-hydrocarbon receptor	*Ahr*	NM_013464	Inhibited	ns	ns	ns	**-1.5**	ns	**-2.5**

<u>Nuclear factor of kappa light polypeptide gene enhancer in B cells 1 (NFKB1)</u>

D site albumin promoter binding protein	*Dbp*	NM_016974	Activated	**3.3**	**3.4**	ns	**2.9**	**1.6**	**2**
Deleted in malignant brain tumors	*Dmbt1*	NM_007769	Activated	**3.3**	-1.5	**3.8**	ns	ns	ns
Nuclear receptor subfamily 1, group D, member 1	*Nr1d1*	NM_145434	Activated	**2.6**	**2.2**	1.3	1.2	-3	**2.5**
Nicotinamide phosphoribosyltransferase	*Nampt*	NM_021524	Activated	**1.7**	**1.5**	**1.6**	1.3	ns	**1.5**
Chemokine (C-X-C motif) ligand 2	*Cxcl2*	NM_009140	Activated	**1.7**	**1.7**	**2**	-1.3	-2.1	**2.2**
Tumor necrosis factor	*Tnf*	NM_013693*	Activated	**1.6**	1.4	**1.5**	1.3	-1.3	**2.1**
Polo-like kinase 2	*Plk2*	NM_152804	Activated	**1.6**	1.4	1.4	ns	-1.5	**1.7**
Chemokine (C-C motif) ligand 3	*Ccl3*	NM_011337	Activated	**1.6**	1.3	**1.6**	**1.7**	ns	**1.7**
Chemokine (C-X-C motif) ligand 3	*Cxcl3*	NM_203320	Activated	**1.5**	1.4	**1.8**	ns	-1.8	**2.7**
Chemokine (C-X-C motif) ligand 1	*Cxcl1*	NM_008176	Activated	1.4	ns	**1.6**	ns	-1.4	1.2
Solute carrier family 22, member 4	*Slc22a4*	NM_019687	Activated	1.3	1.3	**1.5**	1.4	ns	**2**
Transferrin receptor	*Tfrc*	NM_011638	Activated	ns	1.3	**1.5**	1.3	**1.7**	1.4
Growth differentiation factor 15	*Gdf15*	NM_011819	Affected	**2.2**	**1.5**	**1.6**	**1.9**	**2.4**	ns
BPI fold containing family A, member 1	*Bpifa1*	NM_011126	Affected	1.4	-1.6	**1.8**	-1.8	-2.4	ns
Surfactant associated protein A1	*Sftpa1*	NM_023134	Affected	1.3	ns	**-1.8**	-1.1	**-2**	-1.4
Epithelial cell adhesion molecule	*Epcam*	NM_008532	Affected	-1.4	-1.4	**-2.2**	**-3.9**	**-2.1**	**-3.3**
Fibroblast growth factor 1	*Fgf1*	NM_010197	Affected	**-1.6**	ns	ns	**-1.8**	1.2	-1.3
Heme oxygenase 1	*Hmox1*	NM_010442	Activated	**2**	**1.6**	**2.6**	-1.3	-2.3	**3.9**
Chemokine (C-C motif) receptor 7	*Ccr7*	NM_007719	Inhibited	**-1.5**	**-1.7**	1.4	**-3**	**-2.1**	1.1
Cardiotrophin-like cytokine factor 1	*Clcf1*	NM_019952	Inhibited	**-1.6**	ns	ns	**-1.6**	-1.4	ns
Class II transactivator	*Ciita*	NM_001243760*	Inhibited	**-1.6**	ns	ns	**-1.9**	-1.3	1.4
CD74 antigen	*Cd74*	NM_001042605*	Inhibited	**-2**	**-1.8**	1.4	**-2.9**	**-1.7**	1.4

TNFAIP3 interacting protein 1	*Tnip1*	NM_021327*	Activated	1.4	ns	1.2	1.4	ns	**1.6**
Tumor necrosis factor, alpha-induced protein 3	*Tnfaip3*	NM_009397*	Activated	ns	ns	1.3	ns	-1.9	**1.6**
Solute carrier family 2, member 6	*Slc2a6*	NM_172659*	Activated	1.4	1.3	ns	1.3	ns	**2**
Radical S-adenosyl methionine domain containing 2	*Rsad2*	NM_021384	Activated	ns	ns	1.2	ns	-1.2	**1.7**
Avian reticuloendotheliosis viral oncogene related B	*Relb*	NM_009046*	Activated	1.3	ns	1.2	1.2	ns	**1.7**
Plasminogen activator, urokinase	*Plau*	NM_008873	Activated	1.4	ns	1.3	1.2	-1.2	**1.5**
Nuclear factor of kappa light enhancer in B cells inhibitor, epsilon	*Nfkbie*	NM_008690	Activated	1.4	1.2	1.4	1.3	-1.3	**2**
Nuclear factor of kappa light enhancer in B cells inhibitor, alpha	*Nfkbia*	NM_010907	Activated	1.4	ns	1.3	1.3	-1.5	**1.5**
Nuclear factor of kappa light enhancer in B cells 2	*Nfkb2*	NM_019408*	Activated	1.2	ns	ns	1.3	-1.4	**1.7**
Interleukin 1 alpha	*Il1a*	NM_010554	Activated	ns	ns	1.2	1.2	ns	**1.6**
Inhibitor of kappaB kinase epsilon	*Ikbke*	NM_019777	Activated	ns	**1.6**	ns	1.3	-1.3	**1.9**
Icos ligand	*Icosl*	NM_015790	Activated	ns	1.2	ns	1.3	-1.3	**1.5**
Fatty acid binding protein 5	*Fabp5*	NM_010634*	Activated	-1.4	**1.5**	-1.6	1.4	-1.1	**2.9**
Elongation factor RNA polymerase II 2	*Ell2*	NM_138953	Activated	ns	1.2	1.3	ns	ns	**1.5**
Chemokine (C-X-C motif) ligand 10	*Cxcl10*	NM_021274	Activated	1.4	ns	1.3	ns	-1.2	**1.6**
Chemokine (C-X3-C motif) ligand 1	*Cx3cl1*	NM_009142	Activated	ns	ns	-1.3	ns	ns	**1.9**
Complement factor B	*Cfb*	NM_008198*	Activated	ns	ns	1.4	ns	-1.7	**1.9**
Chemokine (C-C motif) ligand 22	*Ccl22*	NM_009137	Activated	-1.4	-1.7	1.4	-1.7	-1.7	**2.4**
Chemokine (C-C motif) ligand 17	*Ccl17*	NM_011332	Activated	-1.2	-1.4	ns	-1.5	-1.6	**1.6**
Superoxide dismutase 2	*Sod2*	NM_013671	Affected	1.4	1.3	1.3	1.2	-1.2	**1.7**
Intracellular adhesion molecule 1	*Icam1*	NM_010493	Inhibited	**1.5**	1.3	ns	**1.7**	ns	**2.1**
Growth arrest and DNA damage inducible 45 alpha	*Gadd45a*	NM_007836	Inhibited	1.4	1.2	1.2	1.4	ns	**1.5**
FBJ osteosarcoma oncogene	*Fos*	NM_010234	Inhibited	**-1.5**	ns	ns	**-2.1**	**-1.8**	**-1.9**
Fibronectin 1	*Fn1*	NM_010233*	Inhibited	-1.3	**-1.5**	-1.3	**-2**	**-2.2**	**-1.6**
Apolipoprotein E	*Apoe*	NM_009696	Inhibited	ns	-1.4	ns	-1.9	-3.9	**2.7**

<u>CCAAT/enhancer binding protein, beta (CEBPB)</u>

C-type lectin domain family 4, member e	*Clec4e*	NM_019948	Activated	**2.4**	**2.3**	**1.6**	1.4	-1.4	**2.8**
Cytochrome P450, family 2, subfamily a, polypeptide 5	*Cyp2a5*	NM_007812	Activated	**2.1**	1.3	1.4	ns	-1.2	ns
Heat shock protein 90, alpha, class A member 1	*Hsp90aa1*	NM_010480	Activated	**1.9**	1.3	1.3	ns	-1.2	ns
WAP four-disulfide core domain 18	*Wfdc18*	NM_007969	Activated	**1.9**	ns	**1.8**	ns	ns	ns
Chemokine (C-X-C motif) ligand 2	*Cxcl2*	NM_009140	Activated	**1.7**	**1.7**	**2**	-1.3	-2.1	**2.2**
Aldehyde dehydrogenase family 1, subfamily A1	*Aldh1a1*	NM_013467	Activated	**1.7**	ns	1.4	ns	-1.2	ns
Tumor necrosis factor	*Tnf*	NM_013693*	Activated	**1.6**	1.4	**1.5**	1.3	-1.3	**2.1**
Chemokine (C-C motif) ligand 3	*Ccl3*	NM_011337	Activated	**1.6**	1.3	**1.6**	**1.7**	ns	**1.7**
Aldo-keto reductase family 1, member B8	*Akr1b8*	NM_008012	Activated	**1.5**	ns	**1.8**	**1.6**	1.4	**2**
Chemokine (C-X-C motif) ligand 1	*Cxcl1*	NM_008176	Activated	1.4	ns	**1.6**	ns	-1.4	1.2
Actin, alpha2, smooth muscle	*Acta2*	NM_007392	Activated	1.4	ns	**1.5**	ns	-1.2	1.4
Hydroxysteroid (17-beta) dehydrogenase 4	*Hsd17b4*	NM_008292	Activated	1.3	ns	**1.5**	ns	ns	**2.9**
Small proline-rich protein 1A	*Sprr1a*	NM_009264	Activated	-1.2	**-1.5**	**-1.5**	ns	-1.3	ns
High mobility group AT-hook 1	*Hmga1*	NM_016660*	Activated	ns	ns	**1.9**	ns	-1.2	**2.1**
Heat shock protein 1	*Hspd1*	NM_010477	Affected	**1.6**	ns	1.2	ns	ns	ns

Matrix Gla protein	*Mgp*	NM_008597	Affected	**1.5**	**1.7**	ns	ns	ns	ns
Hemoglobin, beta adult major chain	*Hbb-b1*	NM_001278161	Affected	**-5.1**	**-2.7**	-1.1	1.4	**-3.1**	1.6
Surfactant associated protein D	*Sftpd*	NM_009160	Inhibited	ns	ns	**-1.7**	ns	**-1.6**	-1.2
Pyruvate dehydrogenase kinase, isoenzyme 4	*Pdk4*	NM_013743	Inhibited	**-1.5**	-1.4	**-2.1**	-1.4	ns	ns
Chemokine (C-C motif) receptor 5	*Ccr5*	NM_009917	Inhibited	**-1.6**	**-1.8**	-1.3	**-2.2**	**-2.4**	ns
Cold inducible RNA binding protein	*Cirbp*	NM_007705	Inhibited	**-1.6**	ns	ns	ns	1.3	-1.3
Stearoyl-coenzyme A desaturase 1	*Scd1*	NM_009127	Activated	ns	-1.4	ns	-1.5	-1.9	**3.6**
Fatty acid binding protein 4, adipocyte	*Fabp4*	NM_024406	Activated	ns	**1.7**	-1.3	**1.7**	**1.6**	**2.2**
Aldo-keto reductase family 1, member B8	*Akr1b8*	NM_008012	Activated	**1.5**	ns	**1.8**	**1.6**	1.4	**2**
Inhibitor of kappaB kinase epsilon	*Ikbke*	NM_019777	Activated	ns	**1.6**	ns	1.3	-1.3	**1.9**
Growth arrest and DNA damage inducible 45 alpha	*Gadd45a*	NM_007836	Activated	1.4	1.2	1.2	1.4	ns	**1.5**
Insulin-like growth factor 1	*Igf1*	NM_010512*	Activated	ns	ns	1.2	**1.5**	**2.3**	**1.6**
Xanthine dehydrogenase	*Xdh*	NM_011723	Activated	1.2	1.3	ns	**1.8**	**1.5**	ns
Nucelar receptor subfamily 1, group H, member 3	*Nr1h3*	NM_013839*	Activated	1.3	ns	ns	**1.6**	ns	1.3
Myelocytomatosis oncogene	*Myc*	NM_001177352*	Activated	-1.2	-1.3	-1.3	**-1.6**	**-1.9**	-1.2
Jun oncogene	*Jun*	NM_010591	Activated	ns	ns	1.2	**-1.6**	**-1.6**	-1.2
Intracellular adhesion molecule 1	*Icam1*	NM_010493	Affected	**1.5**	1.3	ns	**1.7**	ns	**2.1**
Colony stimulating factor 3 receptor	*Csf3r*	NM_007782*	Affected	**-1.5**	ns	-1.2	**-1.7**	**-1.5**	**-2**
Chemokine (C-C motif) ligand 5	*Ccl5*	NM_013653	Affected	ns	-1.4	ns	**-1.6**	-1.3	ns
Adenomatous polyposis coli down-regulated 1	*Apcdd1*	NM_133237	Inhibited	ns	ns	-1.4	-1.2	-1.2	**-1.5**
Diacylglycerol o-acyltransferase 2	*Dgat2*	NM_026384	Inhibited	ns	-1.3	-1.2	**-1.7**	**-1.9**	**-1.6**
UDP glucuronosyltransferase 1 family, polypeptide A6B	*Ugt1a6b*	NM_201410	Inhibited	1.2	ns	ns	ns	1.3	**-1.8**
FBJ osteosarcoma oncogene	*Fos*	NM_010234	Inhibited	**-1.5**	ns	ns	**-2.1**	**-1.8**	**-1.9**
Cd14 antigen	*Cd14*	NM_009841	Inhibited	-1.3	-1.4	ns	-1.2	**-2.4**	**-2.1**
Cyclin D2	*Ccnd2*	NM_009829	Inhibited	ns	1.3	ns	**1.8**	**1.5**	1.4
Transmembrane protein 176B	*Tmem176b*	NM_023056*	Inhibited	ns	-1.4	ns	**-1.6**	**-1.5**	ns
Interleukin 12b	*Il12b*	NM_008352	Inhibited	ns	-1.3	ns	**-1.6**	-1.3	ns
CCAAT/enhancer binding protein, alpha (CEBPA)									
Lactotransferrin	*Ltf*	NM_008522	Activated	**2.4**	-1.8	**3.6**	ns	-1.2	ns
Cytochrome P450, family 2, subfamily a, polypeptide 5	*Cyp2a5*	NM_007812	Activated	**2.1**	1.3	1.4	ns	-1.2	ns
Heme oxygenase 1	*Hmox1*	NM_010442	Activated	**2**	**1.6**	**2.6**	-1.3	-2.3	**3.9**
Secretoglobin, family 3A, member 2	*Scgb3a2*	NM_001289643*	Activated	**1.9**	ns	**1.5**	-1.2	-1.4	ns
Secretoglobin, family 1A, member 1	*Scgb1a1*	NM_011681	Activated	**1.6**	-1.3	**1.7**	-2.3	-2.1	-1.2
Cathepsin K	*Ctsk*	NM_007802	Activated	**1.5**	ns	**1.9**	**1.6**	**1.5**	**1.6**
Aldo-keto reductase family 1, member B8	*Akr1b8*	NM_008012	Activated	**1.5**	ns	**1.8**	**1.6**	1.4	**2**
Carboxylesterase 1D	*Ces1D*	NM_053200	Activated	**1.5**	ns	ns	ns	ns	ns
Epoxide hydrolase 1	*Ephx1*	NM_010145	Activated	1.4	ns	**1.5**	**1.8**	1.4	**2.4**
Small proline-rich protein 1A	*Sprr1a*	NM_009264	Activated	-1.2	**-1.5**	**-1.5**	ns	-1.3	ns
Heat shock protein 5	*Hspa5*	NM_001163434*	Activated	**2**	ns	**1.5**	ns	-1.7	ns
Argininosuccinate synthetase 1	*Ass1*	NM_007494	Affected	ns	ns	**1.6**	ns	ns	ns
Surfactant associated protein A1	*Sftpa1*	NM_023134	Affected	1.3	ns	**-1.8**	-1.1	**-2**	-1.4

Surfactant associated protein D	*Sftpd*	NM_009160	Inhibited	ns	ns	**-1.7**	ns	**-1.6**	-1.2
Surfactant associated protein B	*Sftpb*	NM_147779*	Inhibited	ns	ns	**-2**	-1.1	**-2**	-1.4
Surfactant associated protein C	*Sftpc*	NM_011359	Inhibited	ns	ns	**-2.6**	ns	**-2.4**	**-1.7**
Nuclear factor, interleukin 3, regulated	*Nfil3*	NM_017373	Inhibited	**-2**	**-1.7**	-1.3	**-2.7**	**-2**	**-1.9**
Stearoyl-coenzyme A desaturase 1	*Scd1*	NM_009127	Activated	ns	-1.4	ns	-1.5	-1.9	**3.6**
Immunoresponsive gene 1	*Irg1*	NM_008392	Activated	**1.7**	**1.6**	1.4	**1.5**	-1.3	**2.8**
Fatty acid binding protein 4, adipocyte	*Fabp4*	NM_024406	Activated	ns	**1.7**	-1.3	**1.7**	**1.6**	**2.2**
Lipoprotein lipase	*Lpl*	NM_008509	Activated	-1.3	-1.4	1.2	-1.5	-1.4	**1.8**
Superoxide dismutase 2	*Sod2*	NM_013671	Activated	1.4	1.3	1.3	1.2	-1.2	**1.7**
Profilin 2	*Pfn2*	NM_019410	Activated	1.4	1.3	1.4	ns	-1.3	**1.6**
Insulin-like growth factor 1	*Igf1*	NM_010512*	Activated	ns	ns	1.2	**1.5**	**2.3**	**1.6**
Growth arrest and DNA damage inducible 45 alpha	*Gadd45a*	NM_007836	Activated	1.4	1.2	1.2	1.4	ns	**1.5**
Transforming growth factor, beta 2	*Tgfb2*	NM_009367	Activated	ns	ns	ns	ns	ns	**-1.6**
ADP-ribosylation factor-like 4C	*Arl4c*	NM_177305	Activated	-1.4	**-1.7**	ns	**-2.5**	**-1.5**	**-1.7**
Platelet-activating factor receptor	*Ptafr*	NM_001081211	Activated	1.3	ns	1.3	1.3	ns	**1.7**
Myelocytomatosis oncogene	*Myc*	NM_001177352*	Activated	-1.2	-1.3	-1.3	**-1.6**	**-1.9**	-1.2
Jun oncogene	*Jun*	NM_010591	Activated	ns	ns	1.2	**-1.6**	**-1.6**	-1.2
Intracellular adhesion molecule 1	*Icam1*	NM_010493	Affected	**1.5**	1.3	ns	**1.7**	ns	**2.1**
Diacylglycerol o-acyltransferase 2	*Dgat2*	NM_026384	Affected	ns	-1.3	-1.2	**-1.7**	**-1.9**	**-1.6**
Alanyl aminopeptidase	*Anpep*	NM_008486	Affected	ns	ns	ns	**1.9**	**2.1**	ns
Galanin	*Gal*	NM_010253	Inhibited	ns	ns	1.2	ns	-1.6	**1.5**
Interleukin 6 receptor, alpha	*Il6ra*	NM_010559	Inhibited	ns	ns	-1.3	-1.2	-1.3	**-1.5**
Adenomatous polyposis coli down-regulated 1	*Apcdd1*	NM_133237	Inhibited	ns	ns	-1.4	-1.2	-1.2	**-1.5**
FBJ osteosarcoma oncogene	*Fos*	NM_010234	Inhibited	**-1.5**	ns	ns	**-2.1**	**-1.8**	**-1.9**
Colony stimulating factor 3 receptor	*Csf3r*	NM_007782*	Inhibited	**-1.5**	ns	-1.2	**-1.7**	**-1.5**	**-2**
Cd14 antigen	*Cd14*	NM_009841	Inhibited	-1.3	-1.4	ns	-1.2	**-2.4**	**-2.1**
High mobility group AT-hook 1, related sequence 1	*Hmga1-rs1*	NM_001166476*	Inhibited	ns	ns	**-2**	ns	ns	**-2.4**
MAP kinase-activated protein kinase 2	*Mapkapk2*	NM_008551	Inhibited	ns	ns	1.3	1.3	ns	**1.6**
Cyclin D2	*Ccnd2*	NM_009829	Inhibited	ns	1.3	ns	**1.8**	**1.5**	1.4
Interleukin 12b	*Il12b*	NM_008352	Inhibited	ns	-1.3	ns	**-1.6**	-1.3	ns
B cell translocation gene 2	*Btg2*	NM_007570	Inhibited	-1.3	ns	ns	**-1.8**	**-1.6**	-1.2
Hydroxyprostaglandin dehydrogenase 15	*Hpgd*	NM_008278	Inhibited	-1.3	-1.3	ns	**-1.8**	**-1.8**	-1.3
X-box binding protein 1 (XBP1)									
Tumor necrosis factor	*Tnf*	NM_013693*	Activated	**1.6**	1.4	**1.5**	1.3	-1.3	**2.1**
Sec24 related gene family, member D	*Sec24d*	NM_027135	Activated	**1.5**	ns	1.3	ns	-1.3	1.3
Stromal cell-derived factor 2-like 1	*Sdf2l1*	NM_022324	Activated	**1.8**	ns	1.2	1.2	-1.4	ns
Protein disulfide isomerase associated 6	*Pdia6*	NM_027959	Activated	**1.6**	ns	ns	ns	-1.2	ns
Protein disulfide isomerase associated 4	*Pdia4*	NM_009787	Activated	**1.6**	ns	ns	ns	-1.3	ns
Hypoxia upregulated 1	*Hyou1*	NM_021395	Activated	**1.6**	ns	ns	ns	-1.4	ns
Heat shock protein 5	*Hspa5*	NM_001163434*	Activated	**2**	ns	**1.5**	ns	-1.7	ns
Heat shock protein 90, beta, member 1	*Hsp90b1*	NM_011631	Activated	**1.6**	ns	ns	1.2	ns	ns

Heat shock protein 90, alpha, class A member 1	*Hsp90aa1*	NM_010480	Activated	**1.9**	1.3	1.3	ns	-1.2	ns
Heat shock protein 90, alpha, class B member 1	*Hsp90ab1*	NM_008302	Activated	**1.7**	1.4	ns	1.3	ns	ns
Heat shock 105kDa/110kDa protein 1	*Hsph1*	NM_013559	Activated	**2.7**	1.3	**1.6**			
Heme oxygenase 1	*Hmox1*	NM_010442	Activated	**2**	**1.6**	**2.6**	-1.3	-2.3	**3.9**
Alpha fetoprotein	*Afp*	NM_007423	Activated	ns	ns	**1.5**	ns	-1.2	1.3
DnaJ (Hsp40) homolog, subfamily C, member 3	*Dnajc3*	NM_008929	Activated	**1.5**	ns	ns	ns	-1.3	ns
DnaJ (Hsp40) homolog, subfamily B, member 9	*Dnajb9*	NM_013760	Activated	**1.6**	ns	ns	ns	-1.6	ns
Calreticulin	*Calr*	NM_007591	Activated	**1.5**	ns	ns	1.2	ns	ns
S100 calcium binding protein A6	*S100a6*	NM_011313	Affected	**-1.7**	**-1.7**	-1.2	**-2.8**	**-2.7**	-1.2
Chemokine (C-X-C motif) ligand 1	*Cxcl1*	NM_008176	Inhibited	1.4	ns	**1.6**	ns	-1.5	1.2
Catalase	*Cat*	NM_009804	Activated	1.3	**1.6**	1.2	**1.6**	ns	**1.8**
Fibronectin 1	*Fn1*	NM_010233*	Activated	-1.3	**-1.5**	-1.3	**-2**	**-2.2**	**-1.6**
Der1-like domain family, member 3	*Derl3*	NM_024440	Affected	1.3	ns	ns	1.3	ns	1.2
Homocysteine and ER stress inducible member 1	*Herpud1*	NM_022331	Affected	1.3	-1.4	1.4	-1.4	**-1.7**	-1.2
Vascular cell adhesion molecule 1	*Vcam1*	NM_011693	Affected	ns	1.1	ns	**1.5**	**2.6**	-1.5
Myelocytomatosis oncogene	*Myc*	NM_001177352*	Affected	-1.2	-1.3	-1.3	**-1.6**	**-1.9**	-1.2
Intracellular adhesion molecule 1	*Icam1*	NM_010493	Inhibited	**1.5**	1.3	ns	**1.7**	ns	**2.1**
E26 avian leukemia oncogene 1	*Ets1*	NM_011808*	Inhibited	-1.2	ns	ns	**-1.8**	-1.4	ns

<u>Early growth response 1 (EGR1)</u>

Growth differentiation factor 15	*Gdf15*	NM_011819	Activated	**2.2**	**1.5**	**1.6**	**1.9**	**2.4**	ns
Heme oxygenase 1	*Hmox1*	NM_010442	Activated	**2**	**1.6**	**2.6**	-1.3	**-2.3**	**3.9**
Chemokine (C-X-C motif) ligand 2	*Cxcl2*	NM_009140	Activated	**1.7**	**1.7**	**2**	-1.3	**-2.1**	**2.2**
Tumor necrosis factor	*Tnf*	NM_013693*	Activated	**1.6**	1.4	**1.5**	1.3	-1.3	**2.1**
Chemokine (C-C motif) ligand 3	*Ccl3*	NM_011337	Activated	**1.6**	1.3	**1.6**	**1.7**	ns	**1.7**
Chemokine (C-C motif) ligand 9	*Ccl9*	NM_011338	Activated	**1.5**	ns	ns	**2**	**-1.8**	**1.6**
Cyclin D2	*Ccnd2*	NM_009829	Activated	ns	1.3	ns	**1.8**	**1.5**	1.4
Intracellular adhesion molecule 1	*Icam1*	NM_010493	Activated	**1.5**	1.3	ns	**1.7**	ns	**2.1**
Heparanase	*Hpse*	NM_152803	Activated	1.3	ns	1.3	**1.7**	**1.5**	ns
Chemokine (C-C motif) ligand 6	*Ccl6*	NM_009139	Activated	1.4	ns	ns	**1.6**	1.2	ns
Malic enzyme 1, NADP(+)-dependent, cytosolic	*Me1*	NM_008615	Activated	1.4	1.2	1.4	**1.6**	ns	**2**
Vascular cell adhesion molecule 1	*Vcam1*	NM_011693	Activated	ns	1.1	ns	**1.5**	**2.6**	**-1.5**
Myelocytomatosis oncogene	*Myc*	NM_001177352*	Activated	-1.2	-1.3	-1.3	**-1.6**	**-1.9**	-1.2
MAX dimerization protein 1	*Mxd1*	NM_010751	Affected	-1.3	ns	ns	**-1.6**	**-1.5**	**-1.5**
Early growth response 1	*Egr1*	NM_007913	Affected	ns	ns	1.4	**-1.8**	**-1.6**	-1.4
Superoxide dismutase 2	*Sod2*	NM_013671	Affected	1.4	1.3	1.3	1.2	-1.2	**1.7**
Growth arrest and DNA damage inducible 45 alpha	*Gadd45a*	NM_007836	Affected	1.4	1.2	1.2	1.4	ns	**1.5**
Jun oncogene	*Jun*	NM_010591	Inhibited	ns	ns	1.2	**-1.6**	**-1.6**	-1.2
Fibronectin 1	*Fn1*	NM_010233*	Inhibited	-1.3	**-1.5**	-1.3	**-2**	**-2.2**	**-1.6**
Chemokine (C-C motif) receptor 2	*Ccr2*	NM_009915	Inhibited	**-1.5**	**-1.5**	ns	**-2**	**-1.6**	ns
Plasminogen activator, urokinase	*Plau*	NM_008873	Inhibited	1.4	ns	1.3	1.2	-1.2	**1.5**

1 - NOTES
C57 = Smoked C57 mice compared to control C57 mice
RAGE = Smoked RAGE mice compared to control RAGE mice
CS = C57 smoked mice compared to RAGE smoked mice
Red font = statistically significant (Fold ≥ 1.5 and FDR < 0.05)
ns = not significant (FDR > 0.05)
* Gene has more than one isoform

Supplementary Table 5. Predicted Upstream Regulators

Cytokines	*Symbol*	RefSeq ID	Prediction	7-Day Fold[1] C57	RAGE	CS
Tumor necrosis factor (TNF)						
Deleted in malignant brain tumors	*Dmbt1*	NM_007769	Activated	**3.3**	-1.5	**3.8**
C-type lectin domain family 4, member e	*Clec4e*	NM_019948	Activated	**2.4**	**2.3**	**1.6**
Growth differentiation factor 15	*Gdf15*	NM_011819	Activated	**2.2**	**1.5**	**1.6**
Sequestosome 1	*Sqstm1*	NM_011018*	Activated	**2**	ns	**1.6**
Heme oxygenase 1	*Hmox1*	NM_010442	Activated	**2**	**1.6**	**2.6**
Thioredoxin reductase 1	*Txnrd1*	NM_001042523*	Activated	**1.7**	**1.5**	**1.6**
Nicotinamide phosphoribosyltransferase	*Nampt*	NM_021524	Activated	**1.7**	**1.5**	**1.6**
Immunoresponsive gene 1	*Irg1*	NM_008392	Activated	**1.7**	**1.6**	1.4
Chemokine (C-X-C motif) ligand 2	*Cxcl2*	NM_009140	Activated	**1.7**	**1.7**	**2**
Tumor necrosis factor	*Tnf*	NM_013693*	Activated	**1.6**	1.4	**1.5**
Mesothelin	*Msln*	NM_018857	Activated	**1.6**	-1.4	**1.8**
Heat shock protein 1	*Hspd1*	NM_010477	Activated	**1.6**	ns	1.2
Glutamate-cysteine ligase, modifier subunit	*Gclm*	NM_008129	Activated	**1.6**	**1.5**	**1.7**
Chemokine (C-C motif) ligand 3	*Ccl3*	NM_011337	Activated	**1.6**	1.3	**1.6**
ATP-binding cassette, subfamily C, member 1	*Abcc1*	NM_008576	Activated	**1.6**	**1.6**	**1.5**
Metallothionein 2	*Mt2*	NM_008630	Activated	**1.5**	1.3	**2**
Matrix Gla protein	*Mgp*	NM_008597	Activated	**1.5**	**1.7**	ns
Interleukin 1 family, member 9	*Il1f9*	NM_153511	Activated	**1.5**	**1.6**	ns
Chemokine (C-X-C motif) ligand 3	*Cxcl3*	NM_203320	Activated	**1.5**	1.4	**1.8**
Chemokine (C-C motif) ligand 9	*Ccl9*	NM_011338	Activated	**1.5**	ns	ns
Aldehyde dehydrogenase 2	*Aldh2*	NM_009656	Activated	**1.5**	1.4	1.3
Aldo-keto reductase family 1, member B8	*Akr1b8*	NM_008012	Activated	**1.5**	ns	**1.8**
Chemokine (C-X-C motif) ligand 1	*Cxcl1*	NM_008176	Activated	1.4	ns	**1.6**
Solute carrier family 22, member 4	*Slc22a4*	NM_019687	Activated	1.3	1.3	**1.5**
Complement component 5a receptor 1	*C5ar1*	NM_007577*	Activated	-1.2	ns	**-1.7**
Fatty acid binding protein 5	*Fabp5*	NM_010634*	Activated	-1.4	1.5	**-1.6**
Vasohibin 1	*Vash1*	NM_177354	Activated	**-1.5**	-1.3	ns
Class II transactivator	*Ciita*	NM_001243760*	Activated	**-1.6**	ns	ns
Chemokine (C-C motif) receptor 5	*Ccr5*	NM_009917	Activated	**-1.6**	**-1.8**	-1.3
Fatty acid binding protein 1	*Fabp1*	NM_0179399	Activated	**-1.7**	ns	-1.3

Histocompatability 2, T region locus 23	*H2-T23*	NM_010398	Activated	ns	ns	**2.2**
Cholesterol 25-hydroxylase	*Ch25h*	NM_009890	Activated	ns	ns	**1.8**
Argininosuccinate synthetase 1	*Ass1*	NM_007494	Activated	ns	ns	**1.6**
Secreted acidic cysteine rich glycoprotein	*Sparc*	NM_009242*	Activated	ns	ns	**-1.9**
Surfactant associated protein B	*Sftpb*	NM_147779*	Activated	ns	ns	**-2**
Heat shock protein 1B	*Hspa1b*	NM_010478	Affected	**3.2**	**1.6**	**3.4**
Polo-like kinase 2	*Plk2*	NM_152804	Affected	**1.6**	1.4	1.4
Metallothionein 1	*Mt1*	NM_013602	Affected	1.4	1.3	**1.7**
NAD(P)H dehydrogenase, quinone 1	*Nqo1*	NM_008706	Affected	1.4	1.4	**1.5**
Cardiotrophin-like cytokine factor 1	*Clcf1*	NM_019952	Affected	**-1.6**	ns	ns
Transferrin receptor	*Tfrc*	NM_011638	Inhibited	ns	1.3	**1.5**
Heparin-binding EGF-like growth factor	*Hbegf*	NM_010415	Inhibited	ns	ns	**-1.5**
Eph receptor A2	*Epha2*	NM_010139	Inhibited	ns	-1.3	**-1.5**
Leucine-rich alpha-2-glycoprotein 1	*Lrg1*	NM_029796	Inhibited	ns	ns	**-1.5**
Clusterin	*Clu*	NM_013492	Inhibited	ns	**-1.5**	**-1.7**
Laminin, gamma 2	*Lamc2*	NM_008485	Inhibited	ns	ns	**-1.8**
Epithelial membrane protein 2	*Emp2*	NM_007929	Inhibited	ns	ns	**-1.8**
Heat shock protein 8	*Hspa8*	NM031165	Inhibited	**2.3**	ns	1.3
Period circadian clock 2	*Per2*	NM_011066	Inhibited	**2.1**	**3**	-1.5
Heat shock protein 90, alpha, class B member 1	*Hsp90ab1*	NM_008302	Inhibited	**1.7**	1.4	ns
Protein disulfide isomerase associated 4	*Pdia4*	NM_009787	Inhibited	**1.6**	ns	ns
Pyruvate carboxylase	*Pcx*	NM_001162946*	Inhibited	**1.6**	**1.5**	ns
Calreticulin	*Calr*	NM_007591	Inhibited	**1.5**	ns	ns
Chemokine (C-C motif) receptor 7	*Ccr7*	NM_007719	Inhibited	**-1.5**	**-1.7**	1.4
Dual specificity phosphatase 1	*Dusp1*	NM_013642	Inhibited	**-1.7**	ns	ns
Interferon gamma (IFNG)						
Heat shock protein 1B	*Hspa1b*	NM_010478	Activated	**3.2**	**1.6**	**3.4**
Solute carrier family 7, member 11	*Slc7a11*	NM_011990	Activated	**2.6**	**2.8**	**2.3**
C-type lectin domain family 4, member e	*Clec4e*	NM_019948	Activated	**2.4**	**2.3**	**1.6**
Growth differentiation factor 15	*Gdf15*	NM_011819	Activated	**2.2**	**1.5**	**1.6**
Heme oxygenase 1	*Hmox1*	NM_010442	Activated	**2**	**1.6**	**2.6**
Sequestosome 1	*Sqstm1*	NM_011018*	Activated	**2**	ns	**1.6**
Heat shock protein 90, alpha, class A member 1	*Hsp90aa1*	NM_010480	Activated	**1.9**	1.3	1.3

WAP four-disulfide core domain 18	*Wfdc18*	NM_007969	Activated	**1.9**	ns	**1.8**
Solute carrier family 11, member 1	*Slc11a1*	NM_013612	Activated	**1.8**	**2**	**1.7**
Cd63 antigen	*Cd63*	NM_001042580*	Activated	**1.7**	1.3	1.3
Chemokine (C-X-C motif) ligand 2	*Cxcl2*	NM_009140	Activated	**1.7**	**1.7**	**2**
Heat shock protein 90, alpha, class B member 1	*Hsp90ab1*	NM_008302	Activated	**1.7**	1.4	ns
Immunoresponsive gene 1	*Irg1*	NM_008392	Activated	**1.7**	**1.6**	1.4
Nicotinamide phosphoribosyltransferase	*Nampt*	NM_021524	Activated	**1.7**	**1.5**	**1.6**
Chemokine (C-C motif) ligand 3	*Ccl3*	NM_011337	Activated	**1.6**	1.3	**1.6**
Heat shock protein 1	*Hspd1*	NM_010477	Activated	**1.6**	ns	1.2
Tumor necrosis factor	*Tnf*	NM_013693*	Activated	**1.6**	1.4	**1.5**
Cathepsin K	*Ctsk*	NM_007802	Activated	**1.5**	ns	**1.9**
Chemokine (C-X-C motif) ligand 3	*Cxcl3*	NM_203320	Activated	**1.5**	1.4	**1.8**
Interleukin 1 family, member 9	*Il1f9*	NM_153511	Activated	**1.5**	**1.6**	ns
Chemokine (C-X-C motif) ligand 1	*Cxcl1*	NM_008176	Activated	1.4	ns	**1.6**
Metallothionein 1	*Mt1*	NM_013602	Activated	1.4	1.3	**1.7**
Hydroxysteroid (17-beta) dehydrogenase 4	*Hsd17b4*	NM_008292	Activated	1.3	ns	**1.5**
Dual specificity phosphatase 1	*Dusp1*	NM_013642	Activated	**-1.7**	ns	ns
Argininosuccinate synthetase 1	*Ass1*	NM_007494	Activated	ns	ns	**1.6**
Cholesterol 25-hydroxylase	*Ch25h*	NM_009890	Activated	ns	ns	**1.8**
Histocompatability 2, M region locus 3	*H2-M3*	NM_013819	Activated	ns	ns	**1.6**
Histocompatability 2, T region locus 23	*H2-T23*	NM_010398	Activated	ns	ns	**2.2**
High mobility group AT-hook 1	*Hmga1*	NM_016660*	Activated	ns	ns	**1.9**
Transferrin receptor	*Tfrc*	NM_011638	Activated	ns	1.3	**1.5**
D site albumin promoter binding protein	*Dbp*	NM_016974	Affected	**3.3**	**3.4**	ns
Nuclear receptor subfamily 1, group D, member 1	*Nr1d1*	NM_145434	Affected	**2.6**	**2.2**	1.3
G protein-coupled receptor 56	*Gpr56*	NM_001198894*	Affected	**2**	-1.3	**-1.8**
Phospholipase A2, group VII	*Pla2g7*	NM_013737	Affected	**1.7**	1.4	**-2.3**
Fibroblast growth factor 1	*Fgf1*	NM_010197	Affected	**-1.6**	ns	ns
Heparin-binding EGF-like growth factor	*Hbegf*	NM_010415	Inhibited	ns	ns	**-1.5**
Laminin, gamma 2	*Lamc2*	NM_008485	Inhibited	ns	ns	**-1.8**
Heat shock protein 8	*Hspa8*	NM031165	Inhibited	**2.3**	ns	1.3
Secretoglobin, family 3A, member 1	*Scgb3a1*	NM_054037*	Inhibited	**2.3**	ns	**1.8**
NAD(P)H dehydrogenase, quinone 1	*Nqo1*	NM_008706	Inhibited	1.4	1.4	**1.5**
Chloride intracellular channel 5	*Clic5*	NM_172621	Inhibited	1.2	ns	**-1.7**

Complement component 5a receptor 1	*C5ar1*	NM_007577*	Inhibited	-1.2	ns	**-1.7**
Small proline-rich protein 1A	*Sprr1a*	NM_009264	Inhibited	-1.2	**-1.5**	**-1.5**
Complement component 4B	*C4b*	NM_009780	Inhibited	-1.3	ns	**-1.9**
Schlafen 4	*Slfn4*	NM_011410	Inhibited	**-1.5**	ns	ns
Chemokine (C-C motif) receptor 5	*Ccr5*	NM_009917	Inhibited	**-1.6**	**-1.8**	-1.3
Class II transactivator	*Ciita*	NM_001243760*	Inhibited	**-1.6**	ns	ns
Cold inducible RNA binding protein	*Cirbp*	NM_007705	Inhibited	**-1.6**	ns	ns
GTPase, very large interferon inducible 1	*Gvin1*	NM_029000*	Inhibited	**-1.7**	1.3	-1.4
CD74 antigen	*Cd74*	NM_001042605*	Inhibited	**-2**	**-1.8**	1.4
Histocompatability 2, class II antigen A, beta 1	*H2-Ab1*	NM_207105	Inhibited	**-2.1**	**-1.6**	1.3
Histocompatability 2, class II antigen A, alpha	*H2-Aa*	NM_010378	Inhibited	**-2.2**	**-1.7**	1.4
Histocompatability 2, class II antigen E, beta	*H2-Eb1*	NM_010382	Inhibited	**-2.3**	**-1.8**	1.4
Hemoglobin, beta adult minor chain	*Hbb-b2*	NM_016956	Inhibited	**-2.9**	**-1.6**	-1.2
<u>Interleukin 1, beta (IL1B)</u>						
Heat shock protein 1B	*Hspa1b*	NM_010478	Activated	**3.2**	**1.6**	**3.4**
Growth differentiation factor 15	*Gdf15*	NM_011819	Activated	**2.2**	**1.5**	**1.6**
Heme oxygenase 1	*Hmox1*	NM_010442	Activated	**2**	**1.6**	**2.6**
Nicotinamide phosphoribosyltransferase	*Nampt*	NM_021524	Activated	**1.7**	**1.5**	**1.6**
Chemokine (C-X-C motif) ligand 2	*Cxcl2*	NM_009140	Activated	**1.7**	**1.7**	**2**
Chemokine (C-C motif) ligand 3	*Ccl3*	NM_011337	Activated	**1.6**	1.3	**1.6**
Tumor necrosis factor	*Tnf*	NM_013693*	Activated	**1.6**	1.4	**1.5**
Chemokine (C-X-C motif) ligand 3	*Cxcl3*	NM_203320	Activated	**1.5**	1.4	**1.8**
Chemokine (C-C motif) ligand 9	*Ccl9*	NM_011338	Activated	**1.5**	ns	ns
Metallothionein 2	*Mt2*	NM_008630	Activated	**1.5**	1.3	**2**
Metallothionein 1	*Mt1*	NM_013602	Activated	1.4	1.3	**1.7**
Chemokine (C-X-C motif) ligand 1	*Cxcl1*	NM_008176	Activated	1.4	ns	**1.6**
Endothelial PAS domain protein 1	*Epas1*	NM_010137	Activated	1.3	ns	**2.1**
Solute carrier family 22, member 4	*Slc22a4*	NM_019687	Activated	1.3	1.3	**1.5**
Fatty acid binding protein 5	*Fabp5*	NM_010634*	Activated	-1.4	1.5	**-1.6**
Chemokine (C-C motif) receptor 5	*Ccr5*	NM_009917	Activated	**-1.6**	**-1.8**	-1.3
Class II transactivator	*Ciita*	NM_001243760*	Activated	**-1.6**	ns	ns
Fatty acid binding protein 1	*Fabp1*	NM_0179399	Activated	**-1.7**	ns	-1.3
Histocompatability 2, T region locus 23	*H2-T23*	NM_010398	Activated	ns	ns	**2.2**

High mobility group AT-hook 1	*Hmga1*	NM_016660*	Activated	ns	ns	**1.9**
Argininosuccinate synthetase 1	*Ass1*	NM_007494	Activated	ns	ns	**1.6**
Secreted acidic cysteine rich glycoprotein	*Sparc*	NM_009242*	Activated	ns	ns	**-1.9**
D site albumin promoter binding protein	*Dbp*	NM_016974	Affected	**3.3**	**3.4**	ns
Nuclear receptor subfamily 1, group D, member 1	*Nr1d1*	NM_145434	Affected	**2.6**	**2.2**	1.3
Immunoresponsive gene 1	*Irg1*	NM_008392	Affected	**1.7**	**1.6**	1.4
CD74 antigen	*Cd74*	NM_001042605*	Affected	**-2**	**-1.8**	1.4
Heparin-binding EGF-like growth factor	*Hbegf*	NM_010415	Inhibited	ns	ns	**-1.5**
Laminin, gamma 2	*Lamc2*	NM_008485	Inhibited	ns	ns	**-1.8**
High mobility group AT-hook 1, related sequence 1	*Hmga1-rs1*	NM_001166476*	Inhibited	ns	ns	**-2**
NAD(P)H dehydrogenase, quinone 1	*Nqo1*	NM_008706	Inhibited	1.4	1.4	**1.5**
Actin, alpha2, smooth muscle	*Acta2*	NM_007392	Inhibited	1.4	ns	**1.5**
Surfactant associated protein A1	*Sftpa1*	NM_023134	Inhibited	1.3	ns	**-1.8**
Chemokine (C-C motif) receptor 7	*Ccr7*	NM_007719	Inhibited	**-1.5**	**-1.7**	1.4
Dual specificity phosphatase 1	*Dusp1*	NM_013642	Inhibited	**-1.7**	ns	ns
S100 calcium binding protein A6	*S100a6*	NM_011313	Inhibited	**-1.7**	**-1.7**	-1.2
Nuclear factor, interleukin 3, regulated	*Nfil3*	NM_017373	Inhibited	**-2**	**-1.7**	-1.3
Colony stimulating factor 2 (CSF2)						
Heat shock 105kDa/110kDa protein 1	*Hsph1*	NM_013559	Activated	**2.7**	1.3	**1.6**
Growth differentiation factor 15	*Gdf15*	NM_011819	Activated	**2.2**	**1.5**	**1.6**
Macrophage receptor with collagenous structure	*Marco*	NM_010766	Activated	**2**	**1.9**	**2.3**
Regenerating islet-derived 3 gamma	*Reg3g*	NM_011260	Activated	**1.9**	ns	**1.5**
Cd63 antigen	*Cd63*	NM_001042580*	Activated	**1.7**	1.3	1.3
Chemokine (C-C motif) ligand 3	*Ccl3*	NM_011337	Activated	**1.6**	1.3	**1.6**
Glutamate-cysteine ligase, modifier subunit	*Gclm*	NM_008129	Activated	**1.6**	**1.5**	**1.7**
Tumor necrosis factor	*Tnf*	NM_013693*	Activated	**1.6**	1.4	**1.5**
Chemokine (C-X-C motif) ligand 3	*Cxcl3*	NM_203320	Activated	**1.5**	1.4	**1.8**
Chemokine (C-C motif) receptor 5	*Ccr5*	NM_009917	Activated	**-1.6**	**-1.8**	-1.3
Sequestosome 1	*Sqstm1*	NM_011018*	Affected	**2**	ns	**1.6**
Heat shock protein 1	*Hspd1*	NM_010477	Affected	**1.6**	ns	1.2
Complement component 5a receptor 1	*C5ar1*	NM_007577*	Affected	-1.2	ns	**-1.7**
Cardiotrophin-like cytokine factor 1	*Clcf1*	NM_019952	Affected	**-1.6**	ns	ns
Heparin-binding EGF-like growth factor	*Hbegf*	NM_010415	Inhibited	ns	ns	**-1.5**

Complement component 4B	*C4b*	NM_009780	Inhibited	-1.3	ns	**-1.9**
Chemokine (C-C motif) receptor 7	*Ccr7*	NM_007719	Inhibited	**-1.5**	**-1.7**	1.4
Class II transactivator	*Ciita*	NM_001243760*	Inhibited	**-1.6**	ns	ns
CD74 antigen	*Cd74*	NM_001042605*	Inhibited	**-2**	**-1.8**	1.4
Histocompatability 2, class II antigen A, beta 1	*H2-Ab1*	NM_207105	Inhibited	**-2.1**	**-1.6**	1.3
Hemoglobin, beta adult major chain	*Hbb-b1*	NM_001278161	Inhibited	**-5.1**	**-2.7**	-1.1
Complement compnent 5 (C5)						
Growth differentiation factor 15	*Gdf15*	NM_011819	Activated	**2.2**	**1.5**	**1.6**
Chemokine (C-X-C motif) ligand 2	*Cxcl2*	NM_009140	Activated	**1.7**	**1.7**	**2**
Cd63 antigen	*Cd63*	NM_001042580*	Activated	**1.7**	1.3	1.3
Tumor necrosis factor	*Tnf*	NM_013693*	Activated	**1.6**	1.4	**1.5**
Chemokine (C-C motif) ligand 3	*Ccl3*	NM_011337	Activated	**1.6**	1.3	**1.6**
Chemokine (C-X-C motif) ligand 3	*Cxcl3*	NM_203320	Activated	**1.5**	1.4	**1.8**
Chemokine (C-X-C motif) ligand 1	*Cxcl1*	NM_008176	Activated	1.4	ns	**1.6**
Complement component 5a receptor 1	*C5ar1*	NM_007577*	Activated	-1.2	ns	**-1.7**
Chemokine (C-C motif) ligand 2 (CCL2)						
Chemokine (C-X-C motif) ligand 2	*Cxcl2*	NM_009140	Activated	**1.7**	**1.7**	**2**
Tumor necrosis factor	*Tnf*	NM_013693*	Activated	**1.6**	1.4	**1.5**
Chemokine (C-C motif) ligand 3	*Ccl3*	NM_011337	Activated	**1.6**	1.3	**1.6**
Chemokine (C-C motif) ligand 9	*Ccl9*	NM_011338	Activated	**1.5**	ns	ns
Cathepsin K	*Ctsk*	NM_007802	Activated	**1.5**	ns	**1.9**
Chemokine (C-X-C motif) ligand 1	*Cxcl1*	NM_008176	Activated	1.4	ns	**1.6**

1 - NOTES
C57 = Smoked C57 mice compared to control C57 mice
RAGE = Smoked RAGE mice compared to control RAGE mice
CS = C57 smoked mice compared to RAGE smoked mice
Red font = statistically significant (Fold ≥ 1.5 and FDR < 0.05)
ns = not significant (FDR > 0.05)
* Gene has more than one isoform
